# Liprin-α proteins are master regulators of human presynapse assembly

**DOI:** 10.1038/s41593-024-01592-9

**Published:** 2024-03-12

**Authors:** Berta Marcó de la Cruz, Joaquín Campos, Angela Molinaro, Xingqiao Xie, Gaowei Jin, Zhiyi Wei, Claudio Acuna, Fredrik H. Sterky

**Affiliations:** 1 Department of Laboratory Medicine, Institute for Biomedicine, Sahlgrenska Academy, University of Gothenburg, Gothenburg, Sweden; 2 Wallenberg Centre for Molecular and Translational Medicine, University of Gothenburg, Gothenburg, Sweden; 3 Chica and Heinz Schaller Foundation, Institute of Anatomy and Cell Biology, Heidelberg University, Heidelberg, Germany; 4 School of Life Sciences, Southern University of Science and Technology, Shenzhen, China; 5 Brain Research Center, Southern University of Science and Technology, Shenzhen, China; 6 Shenzhen Key Laboratory of Biomolecular Assembling and Regulation, Shenzhen, China; 7 Department of Clinical Chemistry, Sahlgrenska University Hospital, Gothenburg, Sweden

**Keywords:** Molecular neuroscience, Cellular neuroscience, Synaptic development

## Abstract

The formation of mammalian synapses entails the precise alignment of presynaptic release sites with postsynaptic receptors but how nascent cell–cell contacts translate into assembly of presynaptic specializations remains unclear. Guided by pioneering work in invertebrates, we hypothesized that in mammalian synapses, liprin-α proteins directly link *trans*-synaptic initial contacts to downstream steps. Here we show that, in human neurons lacking all four liprin-α isoforms, nascent synaptic contacts are formed but recruitment of active zone components and accumulation of synaptic vesicles is blocked, resulting in ‘empty’ boutons and loss of synaptic transmission. Interactions with presynaptic cell adhesion molecules of either the LAR-RPTP family or neurexins via CASK are required to localize liprin-α to nascent synaptic sites. Liprin-α subsequently recruits presynaptic components via a direct interaction with ELKS proteins. Thus, assembly of human presynaptic terminals is governed by a hierarchical sequence of events in which the recruitment of liprin-α proteins by presynaptic cell adhesion molecules is a critical initial step.

## Abstract (web summary)

This paper identifies the evolutionarily conserved liprin-α protein family as key mediators of presynaptic assembly in human neurons. Their recruitment to sites formed by contacting neurons is the critical initial step that triggers presynaptic differentiation.

## Main

The brain exerts its functions through the propagation and processing of signals across synapses, which define the neural connectivity and constitute its minimal functional units. The establishment of synaptic connections peaks during development but continues throughout life; recent studies in mice suggest that a substantial fraction of synapses are continuously turned over also in the adult brain^1,2^. The resulting circuitry is refined by neuronal activity, but morphologically normal synapses form also in absence of synaptic transmission, emphasizing underlying cell biological mechanisms^3–5^.

On the presynaptic side, synapse formation entails assembly of the ‘active zone’, an electron-dense region adjacent to the plasma membrane defined as the site of synaptic vesicles exocytosis. The active zone is formed by a conserved set of intracellular scaffolding proteins, namely members of the RIM, RIM-BP, Munc13, bassoon/piccolo, ELKS and liprin-α protein families, which together form a protein interaction network that organizes the essential presynaptic components: the synaptic vesicle pool, molecular machinery for synaptic vesicle exocytosis and voltage-gated calcium channels^6–8^. The synaptic vesicle release sites precisely align with nanometer precision to receptors on the postsynaptic membrane^9^, but how the pre- and postsynaptic structures are coordinately assembled is not well understood^5,10^. A plausible mechanism involves *trans*-synaptic interactions between distinct pairs of synaptic cell adhesion molecules (CAMs), known to influence the molecular architecture and properties of specific synapses^11–13^. Despite numerous studies, major questions remain concerning the signal(s) that initiate active zone assembly; for example, do specific presynaptic CAMs have an instructive role in presynapse formation? If so, what intracellular effectors mediate this signal?

Major presynaptic CAMs include neurexins (reviewed in ref. ^14^) or the leukocyte antigen-related receptor protein tyrosine phosphatase (LAR-RPTPs, reviewed in refs. ^15,16^). Early studies demonstrated that their artificial clustering on axonal membranes induced presynapse assembly^17,18^, suggesting an instructive role. However, loss-of-function studies in mice have failed to support this hypothesis as mice lacking all LAR-RPTPs^19,20^ or all major neurexin isoforms^21–23^ show largely normal numbers of synapses, albeit with altered functional properties. Moreover, knockouts of active zone components generally display impaired neurotransmitter release, but with surprisingly subtle defects in presynapse number or ultrastructure (for example, see ref. ^24^). Altogether, these studies have led to the view of the active zone as a resilient structure without a single ‘master organizer’ governing its assembly^7,25^.

The liprin-α proteins represent prime candidates for organizing presynaptic assembly of mammalian synapses. Liprin-α were first identified as intracellular ligands of LAR-RPTPs and enriched at focal adhesions in nonneuronal cells^26,27^. Subsequent pioneering genetic screens in *Caenorhabditis elegans* identified the liprin-α ortholog SYD-2 as a central player in the differentiation of presynaptic terminals^28^. Further elegant experiments in the *C.* *elegans* hermaphrodite-specific neuron (HSN) synapse found SYD-2/liprin-α to act downstream of the presynaptic adhesion receptor SYG-1 (ref. ^29^) and upstream of active zone assembly and recruitment of synaptic vesicles^30,31^. This function in presynapse formation involves ELKS and requires both liprin-α and ELKS to undergo liquid–liquid phase separation (LLPS)^30,32^. In mammals, liprin-α proteins are encoded by four genes (*PPFIA1–4*, encoding liprin-α1–4), displaying either broad (*PPFIA1*) or nervous system-enriched (*PPFIA2–4*) expression^33^. All liprin-α isoforms share the same domain architecture, with N-terminal coiled-coil regions, a largely unstructured central region and three C-terminal sterile alpha motif (SAM) domains^6,26,34^. The coiled-coil regions mediate homodimerization and interactions with active zone proteins RIM and ELKS^6,35,36^, while the SAM domains bind the intracellular domain of LAR-RPTPs^26,37,38^, suggesting that liprin-α can act downstream of LAR-RPTP(s) or its corresponding ortholog. Mammalian liprin-α isoforms that contain a splice insertion between the first two SAM domains can additionally bind to the scaffolding protein CASK^39,40^, thereby indirectly linking liprin-α to presynaptic CAMs of neurexin^39^, SynCAM^41^ and syndecan^42^ families. While this suggests that liprin-α proteins play a role in the formation of presynaptic specializations also in mammals, mouse knockouts for liprin-α3 (ref. ^43^) or combinations of the two major isoforms in the nervous system, liprin-α2 and liprin-α3 (ref. ^44^), show relatively subtle alterations in active zone composition and sizes of synaptic vesicle pools.

In this Article, we test the hypothesis that liprin-α proteins are central hubs for presynapse formation in mammals by linking presynaptic CAMs to active zone assembly and synaptic vesicle recruitment. Using human neurons knockout for all four liprin-α genes (*PPFIA1–4*), we reveal a highly redundant mechanism: liprin-α proteins are essential for the formation of structural and functional presynaptic terminals, and their recruitment by any of multiple presynaptic CAMs is a necessary initiating signal.

## Results

### Loss of liprin-α1–4 mislocalizes presynaptic components

**Fig. 1:**
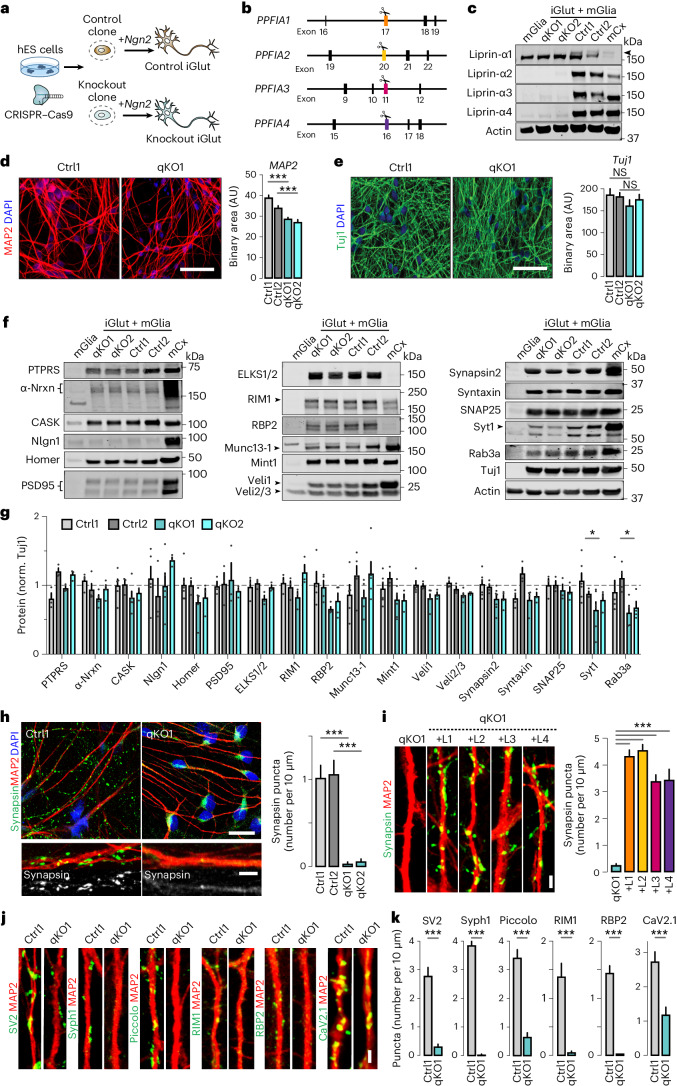
Deletion of all liprin-α proteins in human neurons causes mislocalization of presynaptic proteins. **a**, The experimental workflow. CRISPR–Cas9 was used to genetically delete all liprin-α proteins from hES cells, subsequently differentiated into iGluts by expression of *Ngn2*. **b**, A schematic of *PPFIA1*, *PPFIA2*, *PPFIA3* and *PPFIA4* genes (encoding liprin-α1, -α2, -α3 and -α4, respectively) with the targeted exons indicated. **c**, Immunoblots for liprin-α1, -α2, -α3 and -α4 of qKO clones and their isogenic controls. Mouse cortex and glia samples were included for reference. The arrowhead indicates neuronal liprin-α1. **d**,**e**, Dendritic (**d**) and axonal (**e**) densities in liprin-α control (Ctrl1 and Ctrl2) and knockout (qKO1 and qKO2) neurons, quantified via immunostaining for MAP2 (**d**) or Tuj1 (**e**). Left, representative images. Scale bar, 50 μm. Right, summary plots. Number of fields/batches, MAP2: Ctrl1, 84/3; Ctrl2, 94/3; qKO1, 94/3; qKO2, 80/3 and Tuj1: Ctrl1, 27/3; Ctrl2, 18/3; qKO1, 20/3; qKO2, 21/3. **f**,**g**, Western blots of synaptic proteins in liprin-α knockout (qKO) or control (Ctrl) iGluts, mouse glia (mGlia) and mouse cortex tissue (mCx). Representative blots (**f**) and summary graphs (**g**) of quantifications normalized to Tuj1 (norm. Tuj1). Dashed line indicates the average value of controls. Syt1, synaptotagmin-1; RBP2, RIM-BP2. Number of cells/batches: *n* = 3–7 (Supplementary Table 1). **h**, Synapsin distribution in liprin-α Ctrl and qKO human iGluts stained for MAP2 (red), synapsin (green) and 4,6-diamidino-2-phenylindole (DAPI) (blue). Left, representative images at low and high magnification, respectively. Right, a summary plot of synapsin puncta density. Number of fields/batches: Ctrl1, 24/2; Ctrl2, 22/2; qKO1, 30/2; qKO2, 28/2. Scale bars, 20 μm (overviews) and 5 μm (inserts). **i**, Rescue of synapsin puncta in liprin-α knockout neurons. Left, representative images of liprin-α knockout (qKO) iGluts, and qKO rescued with liprin-α1 (+L1), liprin-α2 (+L2), liprin-α3 (+L3) and liprin-α4 (+L4). Scale bar, 2 μm. Right, summary graphs of synapsin puncta densities. Number of cells/batches: qKO1, 57/4; qKO1 + L1, 62/4; qKO1 + L2, 66/4; qKO1 + L3, 64/4, qKO1 + L4, 32/2. **j**,**k**, Distribution of synaptic vesicle glycoproteins 2 (SV2), Syph1 (synaptophysin-1), piccolo, RIM1, RIM-BP2 and CaV2.1 in liprin-α control (Ctrl) and knockout (qKO) neurons. Representative images (**j**) of the indicated protein (green) and MAP2 (red) and summary plots of puncta densities (**k**). Scale bar, 2 μm. Number of fields/batches: Ctrl1, 28–67/2–3 and qKO1, 20–54/2–3 (Supplementary Table 1). Data are represented as means ± s.e.m. NS, not significant; *P < 0.05; and ***P < 0.001. Source data

**Extended Data Fig. 1:**
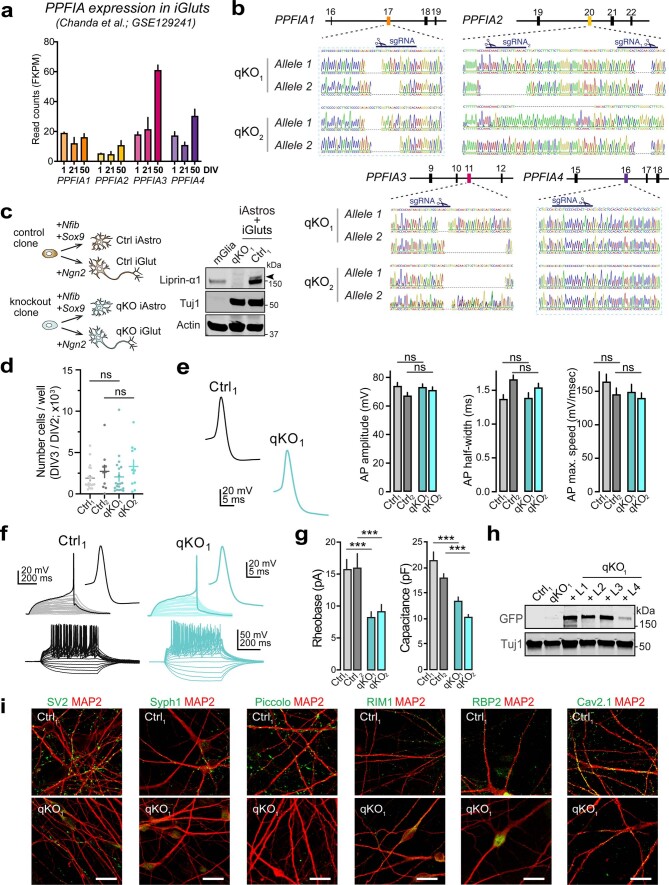
Liprin-α expression, validation of Liprin-α knockouts, and basic physiological and morphological characterization of qKO neurons (related to Figure 1). (**a**) Expression of *PPFIA1-4* in *Ngn2*-induced iGluts at the indicated day *in vitro* (DIV). Data from GSE129241 (ref. ^90^). (**b**) Sanger sequencing traces across gene-edited exons of *PPFIA1-4*. The mutant clones share the same mutations in *PPFIA1* and *PPFIA4*. (**c**) Experimental outline (left) and western blots (right) of experiment to confirm removal of liprin-α1 in human *Ngn2*-induced iGluts by co-culture with human induced astrocytes^47^. Arrowhead indicates the liprin-α1 isoform migrating at a higher apparent molecular weight. (**d**) Conversion rates for iGluts derived from control (Ctrl1 and Ctrl2) and mutant (qKO1 and qKO2). Number of cells/well were counted upon replating at DIV3 (after Puromycin selection) and normalized to the number of ES-cells seeded at DIV2. Number of batches: Ctrl1,20; Ctrl2,10; qKO1,20; qKO2,11. (**e**) Action potential properties of iGluts derived from control (Ctrl1 and Ctrl2) and mutant (qKO1 and qKO2) clones. Left to right: amplitude, half-width, and max. speed. Number of cells/batches: Ctrl1,24/2; Ctrl2,22/2; qKO1,23/2; qKO2,23/2. (**f**) Top. Estimation of minimal current required to trigger spikes (Rheobase). Stimulus: 500 ms, +1 pA steps. Bottom. Voltage responses to current injections of increasing amplitude (500 ms, 5 pA steps from -25 to +75 pA). (**g**) Summary plots of rheobase and capacitance in induced neurons derived from two control (Ctrl1, Ctrl2) and two knockout (qKO1, qKO2) hESC lines. Number of fields/batches: Capacitance; Ctrl1, 45/3; Ctrl2, 30/3; qKO1, 40/3; qKO2, 31/3. Rheobase; Ctrl1, 40/3; Ctrl2, 31/3; qKO1, 37/3; qKO2, 40/3. (**h**) Western blot of Liprin-α protein expression in rescue experiments (Figs. 1i and 3g, h). (**i**) Low-magnification images of Ctrl and qKO neurons immunolabeled for SV2 (synaptic vesicle glycoprotein 2), Sph1 (synaptophysin-1), Piccolo, RIM1, RBP2 (RIM-BP2) and CaV2.1, shown in Fig. 1j. Scale bars, 20 μm. Data represented as means ± SEM; ns, non-significant; *** p < 0.001. Source data

To assess the role of liprin-α proteins (collectively referred to here as liprin-α) in human presynapse assembly, we deleted all liprin-α in human embryonic stem cells (hES cells). We chose this model system for relevance to human disease, genetic access and ability to convert into different types of synaptically active induced neurons rapidly and efficiently by means of forced expression of defined transcription factors^45–47^. Induced human glutamatergic neurons (iGluts) express all four liprin-α genes (*PPFIA1–4*) (ref. ^48^). Expression of *PPFIA2–4* increases during differentiation while *PPFIA1* remains stable (Extended Data Fig. 1a), consistent with the expression of liprin-α across mouse tissues^33^. We introduced disrupting insertions/deletions (indels) in ubiquitous liprin-α exons using clustered regularly interspaced short palindromic repeats–associated protein 9 (CRISPR–Cas9) editing (Fig. 1a,b). Following sequential rounds of gene editing, clonal isolation and analysis, we obtained cells carrying frame-shifting homozygous or compound heterozygous indels in *PPFIA1–4*, as confirmed by fragment analysis and Sanger sequencing (Extended Data Fig. 1b). Clones subjected to similar treatment but without detected mutations were used as isogenic controls. Removal of liprin-α in quadruple knockout (qKO) iGluts was confirmed by western blot analysis (Fig. 1c). Expression of liprin-α1 in mouse glial cells^33^ cocultured to support the induced neurons partially masked the larger neuronal isoform. We therefore confirmed complete removal of liprin-α1 in a separate experiment (Extended Data Fig. 1c), replacing the mouse glia cells with lineage-converted human induced astrocytes^47^ derived from the same clone.

We next differentiated control (Ctrl1 and Ctrl2) and mutant (qKO1 and qKO2) clones into iGluts. Liprin-α qKO lines converted into induced neurons at rates similar to control lines (Extended Data Fig. 1d). Neurons from knockout lines showed an ~20% reduction in the total somatodendritic area labeled by MAP2 (Fig. 1d), while the total area occupied by β3-tubulin (Tuj1)-labeled axons was similar (Fig. 1e). Patch-clamp electrophysiology revealed that all cells, regardless of genotype, fired action potentials with normal amplitudes and kinetics (Extended Data Fig. 1e,f). However, mutant cells displayed ~35–40% reductions in rheobase and capacitance (Extended Data Fig. 1g), respectively, consistent with the observed changes in MAP2 signals. We performed western blot analysis to determine whether liprin-α removal impacted pre- and postsynaptic protein levels in mutant lines. Of all proteins tested, small but significant reductions were only found in levels of the synaptic vesicle-related proteins synaptotagmin-1 and Rab3A (Fig. 1f,g). Next, we addressed whether removal of liprin-α affected formation of presynaptic specializations by staining human iGluts for synapsin (Fig. 1h). Remarkably, while control neurons displayed typical synaptic punctate signals in close proximity to postsynaptic MAP2-labeled dendrites, mutant neurons completely lacked corresponding punctate synapsin signal, which instead accumulated ectopically and diffusely in cell bodies and axons, similar to the pattern observed in immature neurons before synapse formation^49^. Re-expression of either liprin-α1–4 isoform by lentiviral transduction readily rescued this phenotype (Fig. 1i and Extended Data Fig. 1h). As liprin-α3 is particularly enriched in presynaptic termini^43^, highly expressed in iGluts (Extended Data Fig. 1a) and relevant to human disease^50^, we primarily used this isoform for subsequent rescue experiments. Last, we addressed the subcellular distribution of additional presynaptic components. We labeled iGluts for the integral synaptic vesicle proteins SV2 and synaptophysin-1; active zone components piccolo, RIM1 and RIM-BP2; and the presynaptic calcium channel CaV2.1 (Fig. 1j and Extended Data Fig. 1i). For all the presynaptic markers tested, liprin-α removal resulted in strong reductions of the typical punctate accumulations observed in controls (Fig. 1k).

### Liprin-α deletion yields ‘empty’ boutons

**Fig. 2:**
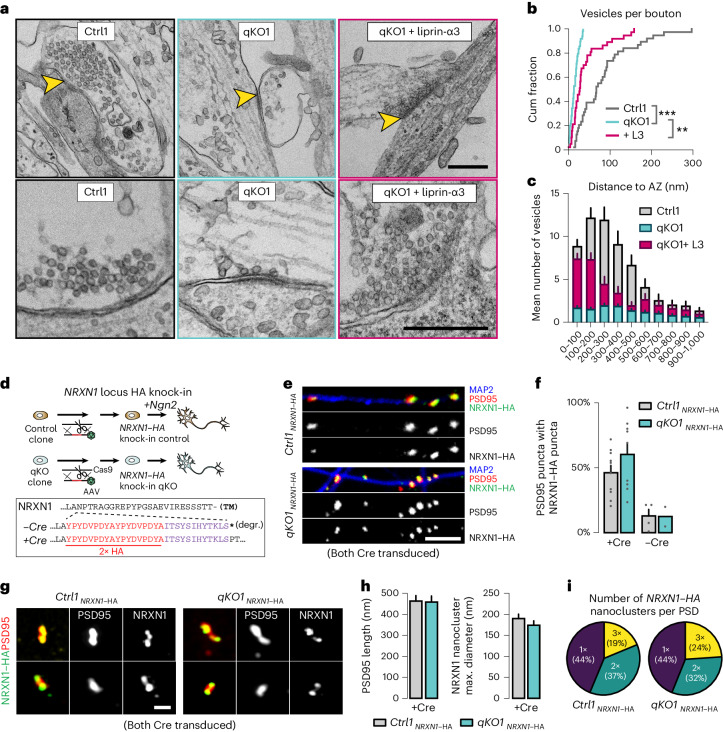
Liprin-α removal blocks assembly of presynaptic terminals. **a**, Transmission electron micrographs of synaptic structures in liprin-α control (Ctrl, left), knockout (qKO, middle) and knockout neurons rescued with liprin-α3 (qKO + liprin-α3, right). Top, low-magnification images with yellow arrowheads highlighting synapse-like structures. Bottom, higher-magnification images highlighting vesicle distribution and abundance. Scale bar, 500 nm. **b**,**c**, Quantitative analysis of the fine structure of synapses (Extended Data Fig. 2a–c). Cumulative (cum) distribution plots of the total number of vesicles per bouton (**b**). Summary plot of the distribution of synaptic vesicles as a function of their distance to the active zone (defined as the presynaptic area opposed to electron-dense postsynaptic structures) (**c**). **d**, Top, the strategy used to introduce an HA tag into the *NRXN1* locus of control and liprin-α qKO hES cells using CRISPR–Cas9 and AAV-mediated homology-directed repair. Insert, the resulting protein sequences of the neurexin-1 juxtamembranous region^54^. Dashed line indicates inserted residues. TM, transmembrane; *, stop codon; degr., degraded. **e**, Representative confocal images of pre-to-postsynaptic appositions between endogenous presynaptic NRXN1 (HA, green) and postsynaptic PSD95 (red) in liprin-α Ctrl and qKO neurons. Dendrites were stained with antibodies against MAP2 (blue). Scale bar, 5 μm. **f**, A summary of the proportion of PSD95 puncta with NRXN1 appositions. Expression of the Cre recombinase is necessary to activate neurexin-1–HA expression; nontransduced neurons that instead express a rapidly degraded truncated form were used as negative controls. Number of coverslips/batches: Ctrl1–*NRXN1–HA[+Cre]*, 10/2; qKO1*–NRXN1–HA[+Cre]*, 9/2; Ctrl1*–NRXN1–HA[*−*Cre]*, 4; and qKO1*–NRXN1–HA[*−*Cre]*, 2/2. **g**, SR-SIM micrographs of endogenous presynaptic NRXN1–HA (green) and postsynaptic PSD95 (red) in liprin-α Ctrl and qKO neurons. Scale bar, 500 nm. **h**, Summary plots of the PSD95 length (left) and NRXN1 nanocluster size (right), defined by the maximum diameter. Number of boutons/batches: Ctrl1 Nrxn–HA + Cre, 32/1 and qKO1 Nrxn–HA + Cre, 25/1. **i**, Quantification of the number of NRXN1 nanoclusters per PSD. Only PSD95-labeled structures with at least one NRXN1 nanocluster were included in the analysis. Data are represented as means ± s.e.m. **P < 0.01 and ***P < 0.001. Source data

**Extended Data Fig. 2:**
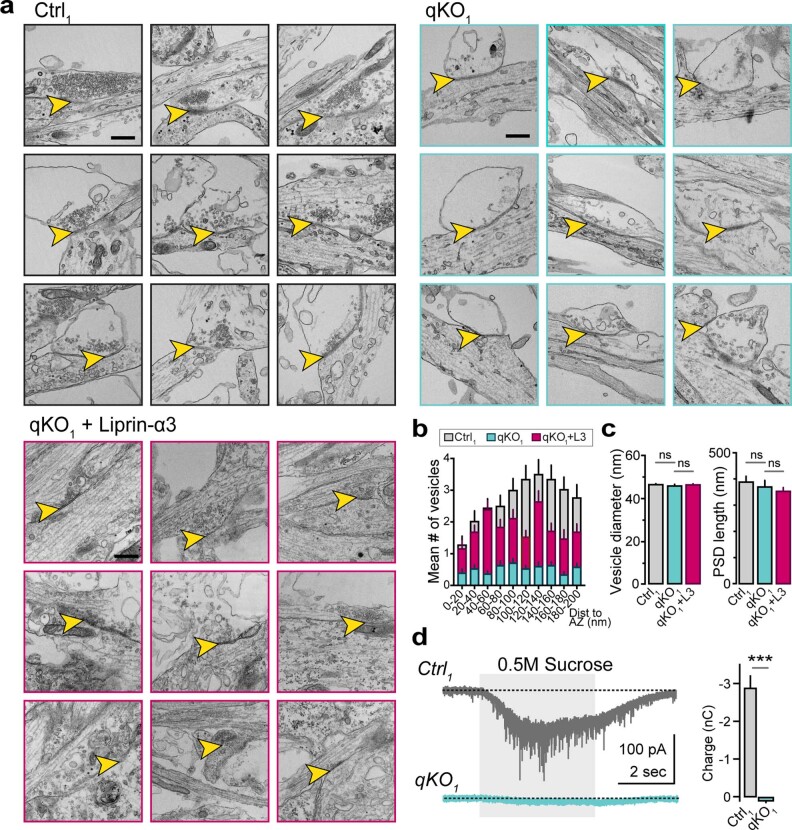
Impact of Liprin-α deletion on synaptic protein recruitment, vesicle recruitment and vesicle pool size (related to Fig. 2). (**a**) Additional electron micrographs showing the fine structure of synapses in control (Ctrl), knockout (qKO), and knockout expressing liprin-α3 (qKO+liprin-α3). (**b**) Summary of distribution of synaptic vesicles in close proximity to the presynaptic active zones in Ctrl, qKO, and qKO rescued with liprin-α3 (qKO + liprin-α3) synapses. Scale bar, 500 nm. (**c**) Summary plots of the vesicle diameter (left) and the postsynaptic density (PSD) length (right) measurements assessed by electron microscopy. Number of vesicles/batches: Ctrl1, 3123/2; qKO, 732/2; qKO + Liprin-α3, 1460/1. Number of PSDs/batches: Ctrl1, 49/2; qKO, 53/2; qKO + Liprin-α3, 41/1. (**d**) Hyperosmotic sucrose response in Ctrl (gray) and qKO (light blue) neurons. Left. Representative responses with duration of sucrose application highlighted (light gray background). Right. Summary plots of the total charge transfer. Number of experiments/batches: Ctrl1, 47/3; qKO1, 59/3. Data in summary plots is represented as means ± SEM; *** p < 0.001.

The removal of liprin-α thus has limited impact on the basic morphology, basic physiology or protein composition of human neurons, but leads to a dramatic change in the subcellular distribution of presynaptic components. To directly assess the impact of liprin-α deletion on presynaptic ultrastructure, we performed transmission electron microscopy (EM) of glutaraldehyde-fixed iGluts. Asymmetric synapses with postsynaptic densities (PSDs) and presynaptic pools of vesicles, observed in control neurons (Fig. 2a and Extended Data Fig. 2a) could not be identified in liprin-α knockouts. Instead, we commonly observed cell–cell junctions with structures resembling PSDs but largely devoid of synaptic vesicles, resembling ‘empty’ boutons (Fig. 2a and Extended Data Fig. 2a). Quantifications confirmed a dramatic decrease in the number of vesicles within such presumed boutons, which was restored upon re-expression of liprin-α3 (Fig. 2a,b). The few vesicles observed in knockout neurons were uniformly distributed without enrichment near presumed active zones opposing PSDs (Fig. 2c and Extended Data Fig. 2b). Notably, re-expression of liprin-α3 predominantly rescued synaptic vesicles in close vicinity to the synaptic cleft (Fig. 2a,c and Extended Data Fig. 2b), consistent with its described subsynaptic localization^43^. The diameter of quantified vesicles or length of the PSDs did not change (Extended Data Fig. 2c). As a functional test of the synaptic vesicle pool, we challenged control and mutant synapses with hypertonic sucrose (0.5 M), known to cause exocytosis of all primed synaptic vesicles at the active zone. Sucrose-evoked responses were robust in control neurons but ablated in knockout neurons (Extended Data Fig. 2d).

**Extended Data Fig. 3:**
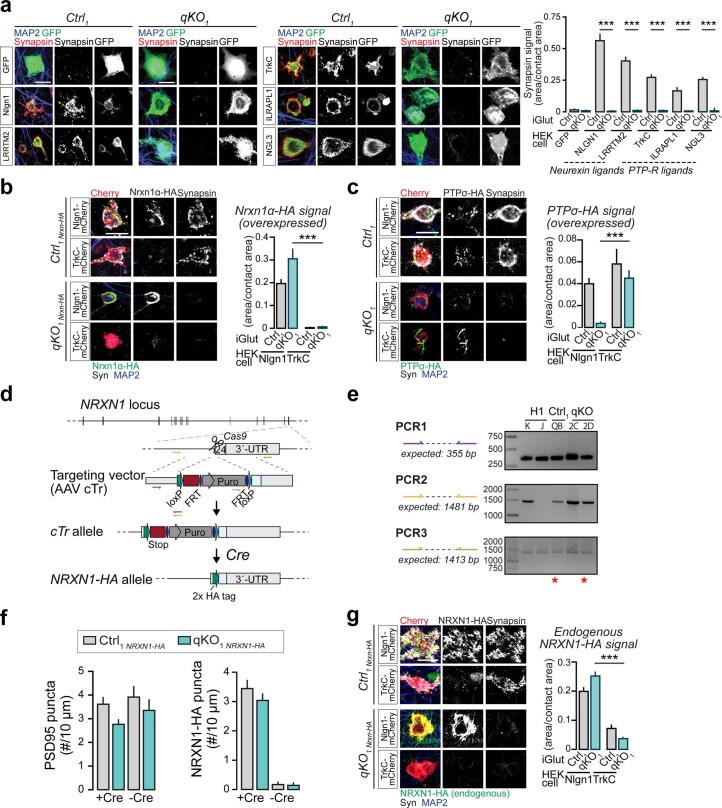
Analysis of presynaptic CAM pathways by heterologous synapses, insertion of an HA-tag in *NRXN1* by homologous recombination and analysis of endogenous NRXN1 distribution (related to Fig. 2). (**a**) Analysis of heterologous synapse formation by different pathways. Left. Representative images of HEK293 cells expressing the indicated GFP-tagged CAMs and co-cultured with control (Ctrl) or knockout (qKO) iGluts. Right. Quantitative analysis of synapsin recruitment to HEK293 cell areas. Number of cells/batches: 44-130/1 (see Supplementary Table 1). All conditions were repeated at least once with similar results. (**b**) Recruitment of overexpressed Nrxn1α-HA onto Nlgn1- or TrkC-expressing HEK293 cells. Left. Representative images. Right. Quantitative analysis of HA-recruitment to HEK293 cell areas. Number of cells/batches: 23-164/2-4 (see Supplementary Table 1). (**c**) Recruitment of overexpressed HA-tagged PTPσ onto Nlgn1- or TrkC-expressing HEK293 cells. Left. Representative images. Right. Quantitative analysis of Ctrl and qKO HA signal area onto HEK293 cells. Intracellular CASK–liprin-α interactions^74^ may explain the partial recruitment of PTPσ-HA by Nlgn1 in control cells. Number of cells/batches: 80-161/3-4 (see Supplementary Table 1). (**d**) Schematic representation of the targeting strategy to insert an HA-tag in the last exon of the *NRXN1* gene. Homologous recombination yields a conditional truncation allele (cTr) producing soluble and rapidly degraded^53^ HA-tagged Neurexin-1. Cre- recombination restores expression of the wildtype, HA-tagged protein. (**e**) PCRs to confirm correct *NRXN1* targeting in control and knockout clones, respectively. Color-coded primer pairs are shown in (**d**). Wildtype H1 cells were included as positive control for gene editing. Clones selected for further experiments marked by asterisks. (**f**) Quantification of PSD95 (left) and NRXN1-HA puncta densities (right) in Ctrl and qKO clones knock-in for NRXN1-HA (related to Fig. 2f). Number of dendrites/batches: 86-281/2 (see Supplementary Table 1). (**g**) Recruitment of endogenous HA-tagged NRXN1 onto Nlgn1- or TrkC-expressing HEK293 cells. Left. Representative images. Right. Quantitative analysis of Ctrl and qKO HA signal area onto HEK293 cells. Number of cells/batches: 90-239/2-3 (see Supplementary Table S1). Data in summary plots is represented as means ± SEM; *** p < 0.001. Source data

The above observations suggested to us that nascent synaptic contacts, possibly involving presynaptic CAMs, indeed form also in absence of liprin-α, but that presynapse assembly may be blocked at a specific downstream step. To test this, we initially used an artificial synapse formation assay to induce formation of presynaptic specializations by defined pathways. We exposed iGluts to neuroligin-1 (Nlgn1) and LRRTM2 to recruit neurexins, or TrkC and ILRAPL1 or NGL3 to recruit the LAR-RPTPs PTPσ and PTPδ, or LAR and PTPσ (refs. ^12,16,51^), respectively. All pathways induced presynaptic synapsin-immunoreactive accumulations in control neurons, while none caused synapsin recruitment in mutant neurons (Extended Data Fig. 3a), pointing to a universal effect downstream of presynaptic CAMs. To test whether artificial pre–post appositions were even formed in absence of liprin-α, we expressed HA-tagged versions of the presynaptic CAMs neurexin-1α or PTPσ in neurons by lentiviral transduction, and induced artificial presynapse formation by coculture with HEK293 cells expressing either postsynaptic neuroligin-1 (Nlgn1, a ligand for neurexins) or TrkC (a ligand for PTPσ; ref. ^52^). In both control and knockout neurons, presynaptic neurexin and PTPσ (Extended Data Fig. 3b,c) were recruited to axonal contacts with cells expressing the matching postsynaptic receptor, indicating that these artificial cell–cell junctions still form in absence of liprin-α.

To assess quantitatively whether liprin-α deletion impacts nascent synaptic contacts formed between iGluts, we analyzed the abundance and size of presynaptic neurexin-1-containing puncta in close apposition to postsynaptic PSD95-containing profiles. To avoid possible artifacts induced by overexpression, and given that antibodies against neurexins do not work well for immunocytochemistry^53^, we utilized a previously validated targeting vector^54^ to knock-in a hemagglutinin (HA) tag in the human *NRXN1* locus of control and qKO clones (Fig. 2d and Extended Data Fig. 3d). Following clonal selection and verification of correct targeting by PCR, we generated *NRXN1*–HA knock-in control and liprin-α qKO iGluts that were *Cre*-transduced to restore *NRXN1* expression (Extended Data Fig. 3e,f). We studied appositions between putative pre- and postsynaptic structures by immunolabeling for HA and PSD95, respectively. Quantification of NRXN1–HA signals in close proximity to PSD95 revealed a high degree of colocalization in both control and qKO neurons, demonstrating that nascent pre–post junctions are formed also in absence of liprin-α (Fig. 2e,f and Extended Data Fig. 3g), consistent with our EM observations. We next used super-resolution structured illumination microscopy (SR-SIM) to study the effect of neurexins organization into synaptic nanoclusters^53^ (Fig. 2g). The number of observed neurexin nanoclusters, their size and distribution across similarly sized PSDs did not change upon removal of liprin-α (Fig. 2h,i). Thus, the mislocalization of synaptic proteins in liprin-α mutants is not caused by a failure to establish pre–post contacts, but due to a block of a downstream step.

### Deletion of liprin-α blocks synaptic transmission

**Fig. 3:**
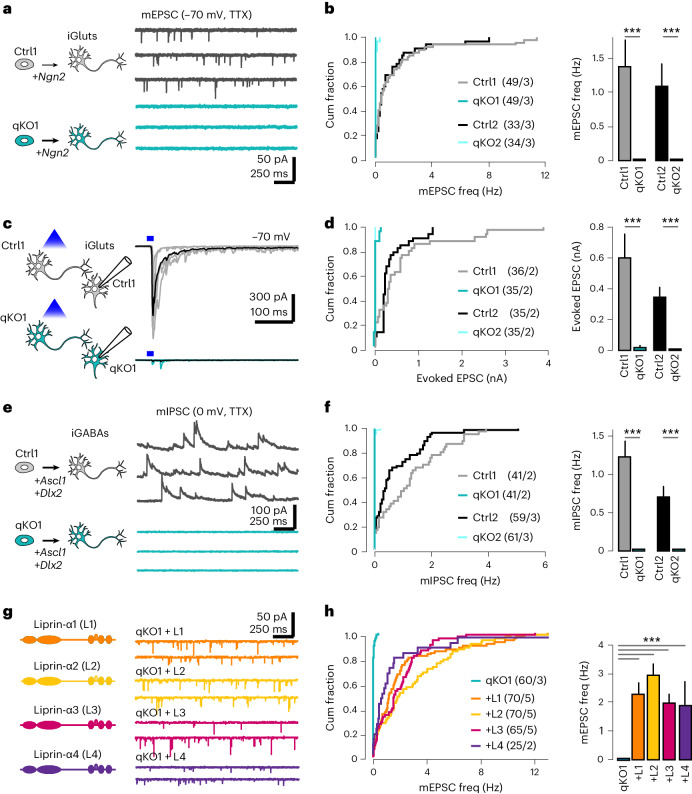
Liprin-α is essential for synaptic transmission. **a**, The effect of liprin-α deletion on spontaneous glutamatergic transmission. Left, control (Ctrl) and knockout (qKO) glutamatergic neurons (iGluts) were derived by forced expression of *Ngn2*. Right, representative recordings of mEPSCs in Ctrl and qKO neurons. **b**, Summary graphs showing the impact of liprin-α removal on the frequency (freq) of mEPSCs. **c**, The impact of liprin-α deletion on evoked glutamatergic synaptic transmission. Left, a schematic of experimental configuration (for details, see Extended Data Fig. 1d). Right, representative recordings of evoked EPSCs in control and knockout neurons. **d**, Summary graphs of evoked glutamatergic transmission in Ctrl and qKO neurons. **e**, The effect of liprin-α deletion on spontaneous GABAergic transmission. Left, Ctrl and qKO GABAergic neurons (iGABAs) were derived by forced expression of Ascl1/Dlx2. Right, representative recordings of mIPSCs in control and knockout neurons. **f**, Summary graphs showing the impact of liprin-α removal on the frequency of mIPSCs. **g**, Rescue experiments in liprin-α knockout neurons. Left, a schematic of liprin-α1, -α2, -α3 and -α4. Right, representative recordings of mEPSCs upon rescue with different liprin-α constructs. **h**, Summary graphs showing mEPSC frequency in knockout neurons, and in qKO neurons expressing liprin-α1, -α2, -α3 and -α4. Data are represented as means ± s.e.m., with the number of cells/batches analyzed indicated in the figures. ***P < 0.001. Source data

**Extended Data Fig. 4:**
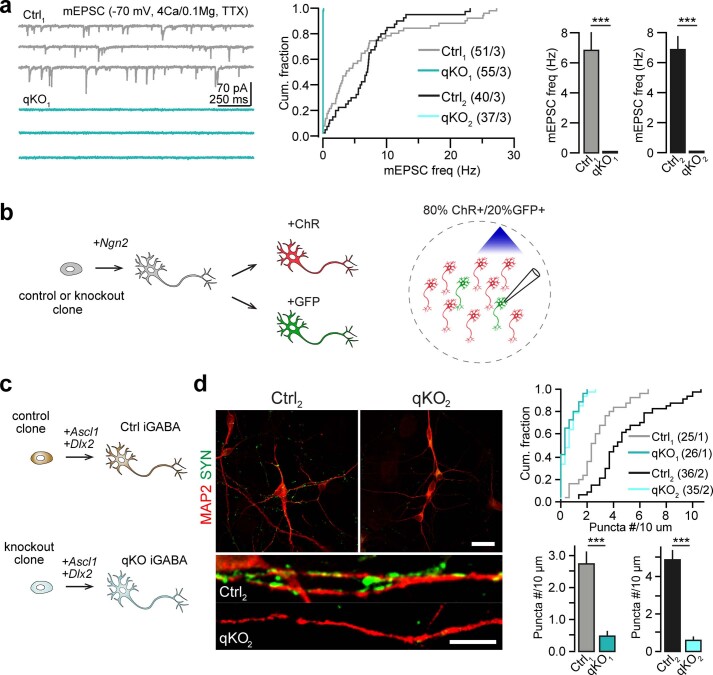
Impact of Liprin-α deletion on excitatory and inhibitory transmission (related to Fig. 3). (**a**) Impact of Liprin-α deletion on miniature release, measured with high (4 mM) extracellular calcium concentration. Left. Representative traces. Middle. cumulative distribution of mEPSC frequencies in iGluts derived from control (Ctrl1 and Ctrl2), and mutant (qKO1 and qKO2) clones, respectively. Right. summary plots of EPSC frequency averages. Number of cells/batches analyzed: indicated in the figure panel in the middle. (**b**) Schematic representation of approach used to record channelrhodopsin-assisted evoked synaptic transmission in Ctrl and qKO mutant iGluts. (**c**) Schematic showing the approach used to derive control or knockout induced GABA neurons (iGABAs). (**d**) Synapsin distribution in Liprin-α Ctrl and qKO human iGABAs stained with antibodies against Synapsin (green) and MAP2 (red). Left, top. Representative images at low magnification. Left, bottom. Higher magnification images. Right. Cumulative distribution plots (top) and summary graphs (bottom) of Synapsin puncta density in iGABAs derived from control (Ctrl1 and Ctrl2) and mutant (qKO1 and qKO2) clones. Number of cells/batches analyzed indicated in the figure. Scale bar, 20 μm (top), 5 μm (lower). Data in summary plots is represented as means ± SEM; *** p < 0.001. Source data

We addressed the functional consequences on synaptic transmission by patch-clamp recordings. First, we performed measurements of miniature spontaneous excitatory currents (mEPSCs, in presence of 0.5 μM tetrodotoxin (TTX)). Remarkably, with near-physiological concentrations of external calcium and magnesium (2 mM Ca^2+^/1 mM Mg^2+^), the frequency of mEPSCs in qKO neurons was reduced by >97% compared with control neurons (Fig. 3a,b). We repeated these experiments with 4 mM Ca^2+^/0.1 mM Mg^2+^ in the external solution, which increased the mEPSC frequency ~twofold in control neurons but remained ~0 Hz in qKO cells (Extended Data Fig. 4a). Next, we measured the impact of liprin-α deletion on evoked synaptic transmission using a channelrhodopsin-assisted approach (Extended Data Fig. 4b) and found spike-mediated neurotransmitter release to be abolished in qKO iGluts (Fig. 3c,d). To corroborate these findings, we determined whether lack of liprin-α similarly impacted inhibitory synapses. Hence, we differentiated control and mutant clones into induced GABAergic cells (iGABAs) by forced expression of *Ascl1* and *Dlx2* (ref. ^46^) (Extended Data Fig. 4c), and measured miniature inhibitory postsynaptic currents (mIPSCs, in the presence of 0.5 μM TTX). Similar to mEPSCs, the frequency of mIPSCs was reduced by >95% in qKO synapses compared with wild-type controls (Fig. 3e,f). Consistently, normal synapsin puncta were also completely absent in qKO iGABA cultures (Extended Data Fig. 4d). Last, we tested whether re-expression of liprin-α by lentiviral transduction could rescue defects in synaptic transmission. Re-expression of any liprin-α1–4 isoform readily rescued mEPSC frequencies (Fig. 3g,h), highlighting the high degree of functional redundancy between the different isoforms.

### Preserved axonal transport of presynaptic components

**Fig. 4:**
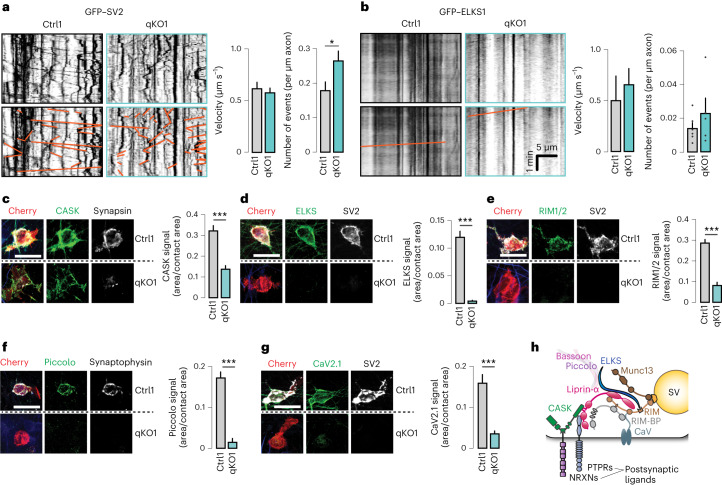
Removal of liprin-α does not affect axonal transport but prevents accumulation of presynaptic proteins at nascent contacts. **a**, The transport of synaptic vesicle cargo in liprin-α control (Ctrl) and mutant (qKO) neurons. Left, representative kymographs depicting GFP-tagged SV2 (GFP–SV2) movements (highlighted in orange in lower images) along axons. Scale as in **b**. Right, summary plots for the velocity and frequency of transport events. Number of fields(particles)/batches: Ctrl1, 13(310)/2 and qKO1, 14(501)/2. **b**, The transport of synaptic vesicle cargo in liprin-α Ctrl and qKO synapses. Left, representative kymographs depicting GFP-tagged ELKS (GFP–ELKS) movements (highlighted in orange in lower images) along axons. Right, summary plots for the velocity and frequency of transport events. Number of fields(particles)/batches: Ctrl1, 10(22)/3 and qKO1, 12(22)/3. **c**–**g**, The recruitment of CASK (**c**), ELKS (**d**), RIM (**e**), piccolo (**f**) and CaV2.1 (**g**) to HEK293 cells expressing Nlgn1. Left, representative images. Right, summary statistics. Number of cells/batches: 79–158/3–4 (Supplementary Table 1). **h**, A schematic model of a presynaptic terminal highlighting the main active zone components, calcium channels and key presynaptic CAMs, based on known protein–protein interactions^6,34^. SV, synaptic vesicle. Scale bars, 20 μm. Data are represented as means ± s.e.m. *P < 0.05 and ***P < 0.001. Source data

The observed morphological and functional defects but near-normal expression of active zone components and synaptic vesicles suggested a failure to recruit these components to nascent synapses. Liprin-α proteins can directly bind to the neuronal kinesin motor KIF1A^55–57^ that mediates axonal transport of synaptic vesicles precursors and dense core vesicles^56,58–60^, suggesting that the mislocalization of presynaptic components may be explained by impaired axonal transport. To test this, we directly assessed axonal transport of synaptic vesicles in iGluts by expression of green fluorescent protein (GFP)-tagged SV2 (GFP–SV2). We performed time-lapse confocal microscopy at day in vitro (DIV)13, a time point when functional synapses are beginning to form^45^, and analyzed moving particles in resulting kymographs (Fig. 4a, left). We observed a higher total number of moving GFP–SV2 particles in liprin-α knockout than control cells, with no difference in the average velocity of movements (Fig. 4a, right). Next, we assessed the axonal transport of piccolo–bassoon transport vesicles by transfecting iGluts with GFP-tagged ELKS1, known to be transported by these vesicles^25,61^. Axonal piccolo–bassoon transport vesicle movements were rare events^25^, but could be observed in both control and liprin-α qKO cells, with a trend toward an increased number of moving GFP–ELKS1 particles in liprin-α qKO cells, with similar velocities (Fig. 4b). Thus, the mislocalization of presynaptic components is not caused by impaired anterograde transport but suggests a failure to recruit those components to nascent nerve terminals.

### Liprin-α proteins are essential for active zone assembly

**Extended Data Fig. 5:**
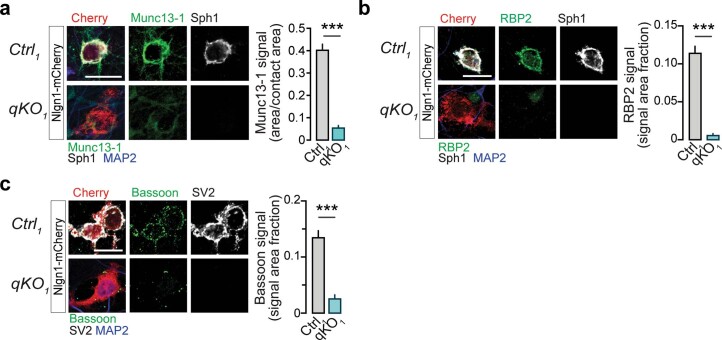
Recruitment of active zone components to heterologous synapses (related to Fig. 4). (**a**) Recruitment of Munc13-1 to HEK293 cells expressing Nlgn1. Left. Representative images. Right. Summary statistics. Sph1, synaptophysin-1. Number of cells/batches: Ctrl1, 101/3; qKO1, 80/3. (**b**) Recruitment of RBP2 (RIM-BP2) to HEK293 cells expressing Nlgn1. Left. Representative images. Right. Summary statistics. Sph1, synaptophysin-1. Number of cells/batches: Ctrl1, 78/3; qKO1, 69/3. (**c**) Recruitment of Bassoon to HEK293 cells expressing Nlgn1. Left. Representative images. Right. Summary statistics. Number of cells/batches: Ctrl1,108/3; qKO1, 99/3. Scale bars, 20 μm. Data represented as means ± SEM; *** p < 0.001. Source data

To systematically assess recruitment of presynaptic scaffolds, active zone proteins and calcium channels to defined sites, we again turned to artificial synapse formation assays. First, we assessed recruitment of the scaffolding protein CASK, which binds to both neurexins^39^ and liprin-α^40^, by postsynaptic neuroligin-1. CASK was recruited to contact sites of both control and qKO neurons (Fig. 4c), although the recruitment was reduced in the liprin-α qKO neurons. In contrast, no recruitment of active zone components ELKS (Fig. 4d), Munc13-1 (Extended Data Fig. 5a), RIM (Fig. 4e), RIM-BP (Extended Data Fig. 5b), piccolo (Fig. 4f) or bassoon (Extended Data Fig. 5c) could be detected at Nlgn1-induced contact sites upon liprin-α deletion, demonstrating a complete loss of active zone formation in qKO neurons. Consistent with this idea and the role of the active zone in organizing presynaptic calcium channels, recruitment of CaV2.1 was also blunted in the knockout neurons (Fig. 4g). Taken together, these results support a model in which liprin-α proteins universally govern presynapse formation upstream of both active zone assembly and synaptic vesicle recruitment, but downstream of presynaptic CAMs, in agreement with the known protein–protein interactions of liprin-α (Fig. 4h)^6,34^.

### Liprin-α–ELKS binding is required for presynapse assembly

**Fig. 5:**
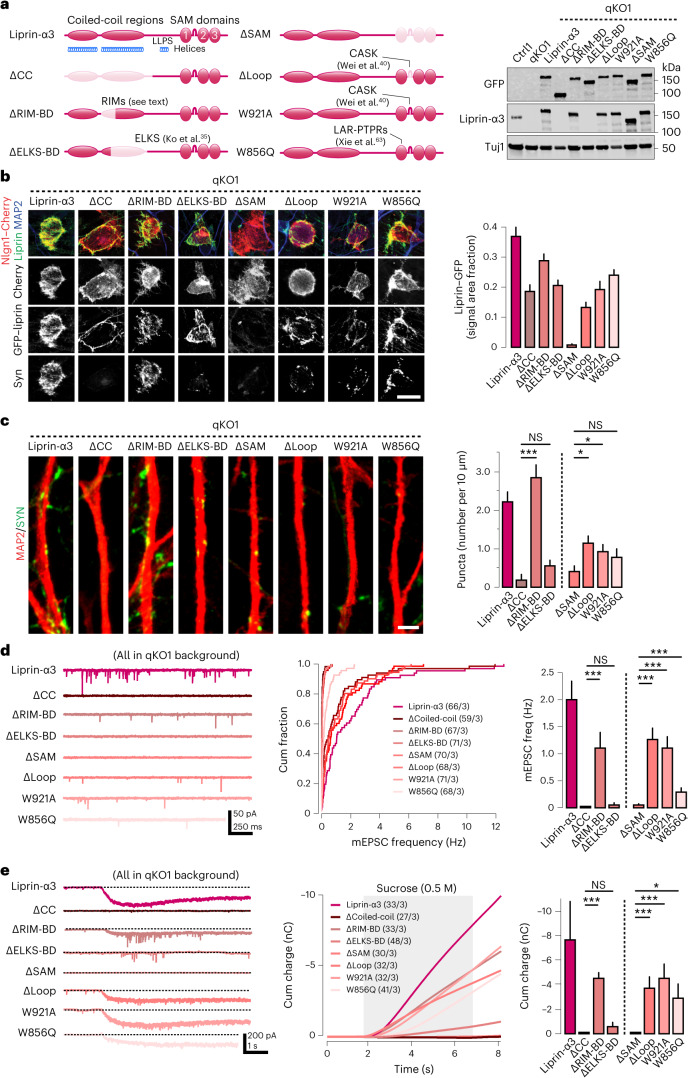
Molecular dissection of liprin-α qKO phenotypes. **a**, Left, schematics of liprin-α3 domain structure and the location and nature of the mutations introduced in different rescue constructs (light red). The name assigned to each rescue construct is shown on the left and references to studies originally describing the mutations are highlighted (see text for details). Right, the expression of all GFP-tagged mutant constructs assessed by western blot, using either anti-GFP or anti-liprin-α3 antibodies. **b**, Recruitment of liprin-α3 mutants to artificial synapses formed onto Nlgn1-expressing HEK cells cocultured with knockout (qKO) iGluts. Left, representative images of the indicated GFP-tagged mutant constructs. Right, summary quantifications of liprin-α3 recruitment. Scale bar, 20 μm. Number of cells/batches: 50–127/1 (Supplementary Table 1). **c**, Rescue of synapsin puncta by different liprin-α mutant constructs expressed in qKO iGluts. Left, representative images of iGluts immunolabeled for MAP2 (red) and synapsin (green). Scale bar, 2 μm. Right, summary plots of synapsin puncta density upon rescue with the different liprin-α mutant constructs. Number of cells/batches: liprin-α3 (WT), 25/2; ΔCC, 22/2; ΔRIM-BD, 25/2; ΔELKS-BD, 29/2; ΔSAM, 24/2; Δloop, 26/2; W921A, 26/2; and W856Q, 30/2. **d**, Rescue of spontaneous glutamatergic transmission by expression of different liprin-α mutant constructs expressed in qKO iGluts. Representative traces (left), cumulative distributions of miniature EPSC frequencies (middle) and summary plots (right). **e**, The effects of liprin-α mutants on hyperosmotic sucrose responses. Representative traces (left), integrated responses (middle) and summary plots (right) of responses in qKO neurons expressing the different liprin-α mutants. Shaded area indicates time of sucrose application. Data are represented as means ± s.e.m. NS, not significant; *P < 0.05; and ***P < 0.001. Source data

To gain insights into what specific functions of liprin-α govern presynapse, we took a molecular replacement approach by generating a battery of liprin-α mutants that disrupt specific domains or interactions (Fig. 5a, left). First, we deleted the entire coiled-coil-enriched N-terminal part of the protein (‘ΔCC’) that mediate homo-oligomerization and interactions with ELKS and RIM proteins^35,36,62^. We also separately deleted smaller segments of the CC2 region: a range of residues (ΔRIM-binding domain, ‘ΔRIM-BD’) found to confer the interaction with RIM proteins (Zhiyi Wei and Gaowei Jin, unpublished), as well as residues (ΔELKS-binding domain, ‘ΔELKS-BD’) required for the interaction with ELKS^35^. Next, we generated a set of mutants in the C-terminal SAM domains: first, a full SAM1/2/3 deletion mutant (‘ΔSAM’), as well as a smaller deletion of the loop insertion between SAM1 and SAM2 (‘ΔLoop’), which mediates interaction with CASK^40^. Moreover, we generated two single amino acid substitutions that have been well characterized by structural studies and direct protein binding analysis: a tryptophan-to-alanine substitution (‘W921A’) that also disrupts binding to CASK^40^ and a tryptophan-to-glutamine substitution (‘W856Q’) blocking the interaction between the SAM123 domains and LAR-RPTPs^63,64^. Western blot analysis confirmed that each mutant was expressed at levels comparable to that of wild-type liprin-α3 (Fig. 5a, right). Next, we assessed whether the liprin-α3 mutants were recruited to presynaptic contact sites using artificial synapses induced by Nlgn1. All mutants except for the ΔSAM were recruited to sites of contact (Fig. 5b).

**Extended Data Fig. 6:**
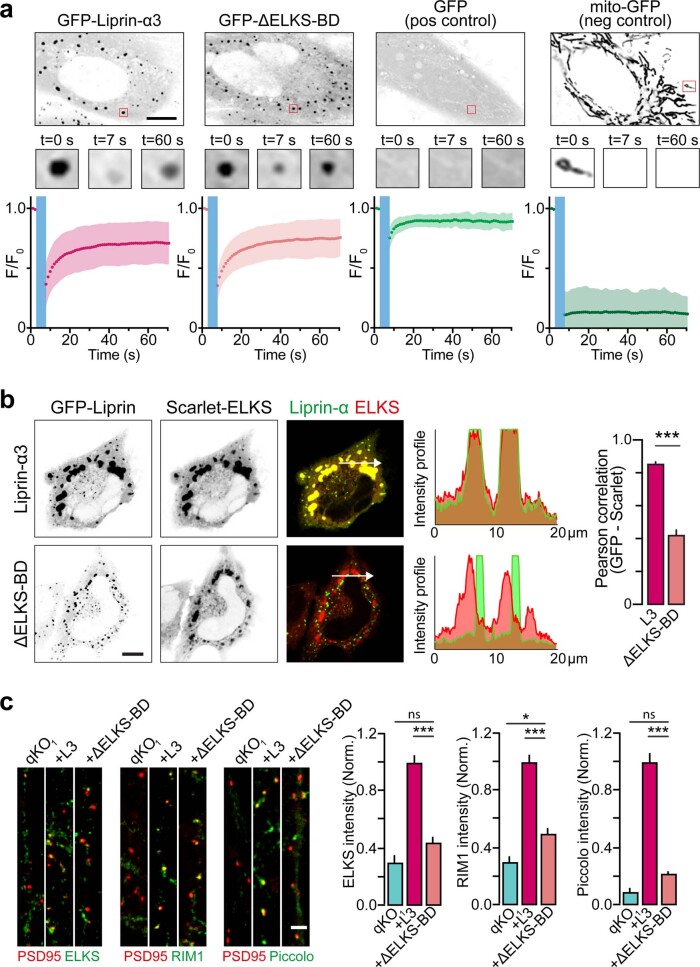
Liprin-α-ELKS interactions promote active zone formation (Related to Fig. 5). (**a**) FRAP-experiments in HeLa cells, performed in presence of PMA (phorbol 12- myristate 13-acetate). Cytosolic regions-of-interests (ROIs, red squares) were photobleached and the fluorescence recovery within each ROI subsequently measured. Cells expressing soluble GFP were included as positive controls for recovery, and cells expressing mitochondrially targeted GFP included as negative controls (by photobleaching isolated mitochondria in their entirety). Traces represent mean ± standard deviation. Number of cells/batches analyzed: GFP-liprin-α3, 30/4; liprin-α3(ΔELKS-BD), 42/4; GFP, 21/2; mito-GFP, 21/2. Scale bar, 10 μm. (**b**) Co-localization between the indicated GFP-tagged liprin-α3 constructs and Scarlet-tagged ELKS in HeLa cells. Left. Representative images, with intensity profiles of green and red fluorescence across the indicated white arrows. Scale bar, 10 μm. Right. Summary graph of green/red colocalization assessed by Pearson correlation coefficient. L3, liprin-α3 (WT). Number of cells/batches analyzed: GFP-liprin-α3, 56/4; liprin-α3(ΔELKS-BD), 46/4. (**c**) Recruitment of active zone proteins ELKS, RIM1, and Piccolo, to presynaptic boutons in qKO iGluts expressing wildtype liprin-α3 (L3) or the ΔELKS-BD mutant. Signal intensity of active zone markers was measured only inside ROIs defined by PSD-95. Representative images (left) and summary plots (right) of fluorescence intensity in qKO neurons expressing different Liprin-α mutants. Scale bar, 2 μm. Number of ROIs/batches: 12-20/1 (see Supplementary Table 1). Data in summary plots is represented as means ± SEM; **p < 0.01, *** p < 0.001. Source data

We then tested the ability of these mutants to rescue synaptic transmission, synapsin puncta signals and sucrose response phenotypes in qKO iGluts (Fig. 5c–e), using wild-type liprin-α3 as reference. We first focused on mutations in the N-terminal coiled-coil regions: rescue with ΔRIM-BD partially restored all phenotypes, whereas full deletion of the coiled-coil region (ΔCC) or removal of the residues that mediate interaction with ELKS failed to rescue any of these phenotypes. Both liprin-α and ELKS can undergo LLPS to support synaptic functions^32,44,62,65^. As the liprin-α region that binds ELKS partially overlaps with that shown to mediate LLPS, we confirmed that the ΔELKS-BD mutant retained its ability to undergo LLPS by fluorescence recovery after photobleaching experiments in Hela cells (Extended Data Fig. 6a). However, when we co-expressed GFP-tagged liprin-α3 together with Scarlet-tagged ELKS, the ΔELKS-BD liprin-α3 mutant accumulated in condensates separate from those formed by ELKS, contrasting the strong colocalization between ELKS and wild-type liprin-α3 (Extended Data Fig. 6b). This finding suggests that presynapse assembly depends on the recruitment of ELKS by liprin-α into shared condensates. As ELKS in such condensates may capture Rab6-containing synaptic vesicles to recruit and organize the presynaptic vesicle pool^66,67^, we tested whether the ELKS interaction specifically prevented synaptic vesicle recruitment, while supporting active zone formation. Hence, we assessed the levels of ELKS, RIM and piccolo at nascent boutons in qKO neurons. Wild-type liprin-α3 rescued the localization of ELKS, RIM and piccolo to boutons in close proximity to PSD95 profiles, but the ΔELKS-BD mutant did not (Extended Data Fig. 6c). Thus, the liprin-α–ELKS interaction is necessary to promote the recruitment of ELKS to nascent boutons, which is essential for both active zone assembly and synaptic vesicle recruitment.

### Presynaptic CAMs initiate presynapse assembly via liprin-α

Next, we studied mutations localized to the SAM domains that mediate direct interactions with presynaptic LAR-RPTPs and indirect interactions with neurexins via CASK. Removal of all liprin-α SAM domains (ΔSAM) did not rescue any phenotype, an expected result given the lack of recruitment of this mutant to artificial presynaptic sites. In contrast, disruption of the liprin-α interaction with CASK, by either the Δloop or W921A mutant, partially rescued synapse numbers, synaptic transmission and sucrose responses (Fig. 5c–e). Similarly, the W856Q mutant blocking interaction with LAR-RPTPs resulted in partial rescues.

**Extended Data Fig. 7:**
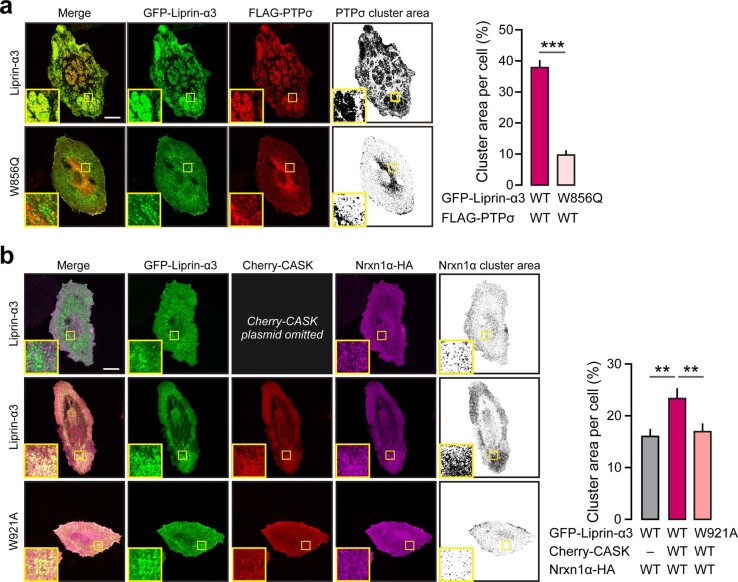
Analysis of Liprin-α-mediated clustering of PTPσ and Neurexin in HeLa cells (related to Fig. 6). (**a**) HeLa cells co-transfected with GFP-tagged Liprin-α3 wildtype or the Liprin-α3 W895Q mutant, and Flag-tagged PTPσ. Left. Representative images with inserts showing enlarged (4x) regions of interest, and quantified (thresholded) signal to the right. Right. Quantification of the PTPσ cluster area per cell. (**b**) HeLa cells co-transfected with HA-tagged Neurexin and GFP-tagged liprin-α3 wildtype or its mutant (W921A) with or without Cherry-tagged CASK. Left. Representative images with inserts showing enlarged (4x) regions of interest and quantified (thresholded) signal to the right. Right. Quantification of the Neurexin cluster area per cell. Scale bars, 20 μm. Data represented as means ± SEM (number of cells/batches: 30/3, per condition). ** p < 0.01, *** p < 0.001. Source data

We hypothesized that these interactions might nevertheless be essential but play a functionally redundant role during initial synapse formation. We first investigated the functional interplay between presynaptic CAMs and liprin-α in nonneuronal cells. Previous studies have shown that liprin-α1 clusters LAR on plasma membranes via a mechanism requiring the N-terminal liprin-α regions^63^. Utilizing the same assay, we first confirmed that liprin-α3 wild type, but not the W856Q mutant, led to clustering of PTPσ (Extended Data Fig. 7a). We next tested whether liprin-α, via CASK, similarly could cluster NRXN1α. Indeed, liprin-α3 together with CASK caused a significant increase in NRXN1α clustering (Extended Data Fig. 7b) compared with the W921A mutant or a condition omitting CASK, although the effect was less pronounced than for PTPσ. No additional synergy was observed upon co-expression of both receptors, suggesting the possibility that each pathway can independently recruit liprin-α to promote bidirectional clustering and presynaptic assembly.

**Fig. 6:**
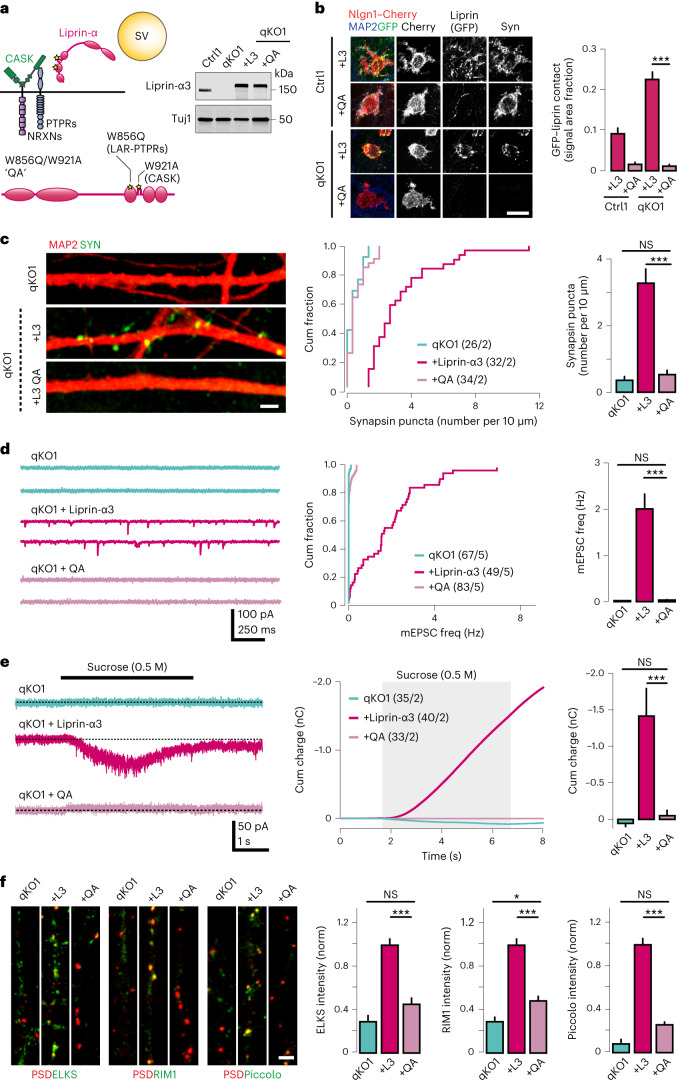
Presynaptic CAMs recruit liprin-α to initiate presynapse assembly. **a**, Left, a schematic of the combined W856Q/W921A mutant, here referred to as ‘QA’, which disrupts the interactions between liprin-α and both LAR-RPTPs and CASK, respectively. Right, an immunoblot demonstrating normal expression of the GFP-tagged QA mutant. **b**, Recruitment of the wild-type liprin-α3 (L3) or the QA mutant to artificial synapses formed onto Nlgn1-expressing HEK cells cocultured with the indicated control (Ctrl) or knockout (qKO) iGluts. Left, representative images of the indicated GFP-tagged mutant constructs. Right, summary quantifications of liprin-α3 recruitment. Scale bar, 20 μm. Number of cells/batches: Ctrl1 + L3, 73/2; Ctrl1 + QA, 188/3; qKO1 + L3, 78/3; and qKO1 + QA, 156/3. **c**, Lack of synapsin puncta rescue by the QA mutant. Left, representative images of qKO iGlut dendrites immunolabeled for MAP2 (red) and synapsin (green). Scale bar, 2 μm. Cumulative distributions (middle) and summary plots (right) of presynapse densities in qKO iGluts expressing the indicated rescue constructs. **d**, Failure of rescue of spontaneous glutamatergic transmission by the QA mutant in qKO iGluts. Representative traces (left), cumulative distributions of miniature EPSC frequencies (middle) and summary plots (right). **e**, Sucrose responses in qKO iGluts expressing wild-type liprin-α3 or the QA mutant. Representative traces (left), integrated responses (middle) and summary plots (right) of responses in qKO neurons expressing the different liprin-α mutants. Shaded area indicates time of sucrose application. **f**, Recruitment of active zone proteins ELKS, RIM1 and piccolo to presynaptic boutons in qKO iGluts expressing wild-type liprin-α3 or the QA mutant. Signal intensity of active zone markers was measured inside ROIs defined by PSD95 and normalized to the wild-type liprin-α3 rescue condition. Representative images (left) and summary plots (right) of fluorescence intensity in qKO neurons expressing different liprin-α mutants. Number of ROIs/batches analyzed: 16–40/2 (Supplementary Table 1). Data are represented as means ± s.e.m. NS, not significant; *P < 0.05; and ***P < 0.001. Source data

To directly test this hypothesis in human neurons, we generated a double mutant construct carrying both W856Q and W921A variants (‘QA’ mutant). We validated the expression of this construct via western blot, and its localization using the artificial synapse assembly assay. Remarkably, although expression of the QA mutant was comparable to that of wild-type liprin-α3 (Fig. 6a), it completely failed to localize to synaptic contact sites (Fig. 6b). We subsequently tested the ability of the QA mutant to rescue synapsin accumulations, synaptic transmission and sucrose responses in qKO iGluts (Fig. 6c–e). We found that the QA mutant failed to rescue synapsin puncta (Fig. 6c), mEPSCs frequency (Fig. 6d) and sucrose responses (Fig. 6e), probably because its mislocalization prevents correct recruitment of synaptic vesicles. Consistent with this, the synapsin signals accumulated ectopically and diffusely in cell bodies and axons upon rescue with the QA mutant. It also failed to recruit ELKS, RIM and piccolo to nascent contact sites (Fig. 6f), indicating a failure not only to recruit synaptic vesicles, but also to induce assembly of presynaptic active zones. Taken together, these results indicate that the interactions between liprin-α and presynaptic CAMs is a critical initial step during synapse assembly, which is necessary to position liprin-α proteins to nascent presynaptic terminals. Synaptically recruited liprin-α then drives active zone formation and recruitment of synaptic vesicles by acting in concert with ELKS.

## Discussion

Here, we find that liprin-α proteins are essential for human presynapse assembly by acting downstream of presynaptic CAMs and thereby serving as their critical effectors. Our work reveals that a fundamental but highly redundant protein interaction network governs presynapse assembly and highlights an instructive role of presynaptic CAMs during early steps of synapse formation. Principal support for these conclusions comes from the following results: (1) genetic removal of all liprin-α isoforms in human neurons impaired presynaptic specializations both morphologically (Figs. 1 and 2) and physiologically (Fig. 3). (2) Normal pre–post contacts containing presynaptic CAMs were formed in absence of liprin-α, but downstream steps were blocked, leading to ‘empty’ boutons (Fig. 2). (3) While presynaptic components were expressed (Fig. 1) and transported (Fig. 4) in the absence of liprin-α, they were massively mislocalized as their recruitment to nascent presynaptic sites was impaired (Figs. 1 and 4). (4) Residues supporting formation of liprin-α–ELKS condensates by means of a direct interaction were required for active zone formation and synaptic vesicle recruitment (Fig. 5). (5) A mutant carrying only two well-characterized single amino acid substitutions that impair binding to both LAR-RPTPs and CASK/neurexin complexes prevented liprin-α recruitment to nascent synaptic sites and bestowed qKO phenotypes.

The substantial morphological and physiological phenotypes we find here contrast many previous genetic studies in mice, *Drosophila* and *C.* *elegans*. In mice, genetic removal of liprin-α2 (ref. ^43^) or combined removal of liprin-α2 and -α3 (ref. ^44^) results in comparatively mild changes in active zone structure and neurotransmitter release, possibly due to functional compensation by other liprin-α family members^7,43^. In agreement with this, we found that re-expression of any liprin-α1–4 could rescue presynapse assembly and neurotransmitter release in qKO neurons (Figs. 1 and 3). In *Drosophila*, genetic removal of Dliprin has been reported to increase^68^ or decrease^69^ the active zone size, and to reduce evoked transmission at the neuromuscular junctions by ~1/3 (refs. ^68,69^). In *C.* *elegans*, removal of liprin-α/SYD-2 seems to cause different phenotypes at different synapses. In GABAergic motor neurons, deletion of liprin-α/SYD-2 causes an increase in the active zone size with no change in the number of synaptic vesicles^28^. At the HSN synapse, liprin-α/SYD-2 deletion instead leads to dramatically reduced synaptic puncta accumulation^30,31^, while synaptic function measured via calcium imaging is reduced by ~50% (ref. ^32^). Last, at the cholinergic motor neuron synapse, removal of liprin-α/SYD-2 causes a decrease in the dense projection size and synaptic vesicle number, and a strong (~70%) reduction in synaptic function^70,71^. Taken together, two conclusions can be drawn from these previous studies. First, that the relative contribution of liprins to the assembly of presynaptic terminals differs between synapses. Second, that other organizers of presynaptic assembly, in addition to liprins, must exist in invertebrates because its genetic removal does not render complete phenotypes in any of these previous studies. In contrast, our study uncovers a uniquely fundamental (‘master’) role of liprin in the assembly of presynaptic terminals in human neurons.

Mechanistically, our results are consistent with a hierarchical model of human synapse assembly, in line with previous work in *C.* *elegans*^29–31^, with liprin-α acting downstream of presynaptic CAMs and upstream of ELKS to support active zone assembly and synaptic vesicle recruitment. In the worm HSN synapse, the adhesion receptor SYG-1 acts upstream of liprin-α/SYD-2. Its subcellular localization, which depends on binding to its postsynaptic ligand SYG-2, determines the intracellular distribution of liprin-α/SYD-2 and sites for presynaptic assembly. In an analogous fashion, synapse formation between human neurons may be initiated by the recruitment of presynaptic CAMs, including LAR-RPTPs, neurexins and/or other CAMs acting via CASK, to nascent contact sites established by neurite guidance cues^5,72^. Subsequent recruitment of liprin-α, by means of direct interactions with LAR-RPTPs and CASK to such sites, orchestrates further assembly of the presynaptic terminal. Notably, we find that a combined point mutant disrupting the interactions with both LAR-RPTPs and CASK fully abolished the ability of liprin-α to rescue presynapse assembly, while each of the mutants separately only displayed partial defects. This finding strongly suggests that presynaptic CAMs play a critical role in the initial steps of synapse formation by positioning liprin-α at the nascent bouton, but that a substantial degree of functional redundancy exists between different pathways. Redundancies between LAR-RPTPs and neurexins may not be surprising given that they can physically interact in *cis* either extracellularly via the heparan sulfate chain on neurexins^73,74^ or intracellularly via CASK–liprin-α interactions^64,75^. Whether this may explain why mice knockout for all LAR-RPTPs^19,20^ or all major neurexin isoforms^22,23^ at most show partial defects in synapse numbers remains unknown, but a recent study indeed demonstrated that combined knockout of all LAR-RPTPs and neurexins (except the NRXN1γ isoform) leads to an ~50% loss of synapse numbers in a cerebellar circuit^76^, supporting this idea. Nevertheless, the defects in synaptic functions observed in these mouse models clearly demonstrates a role for LAR-RPTPs and neurexins in defining functional properties of synapses beyond their formation. A plausible explanation for this apparent discrepancy is that presynaptic CAMs serve multiple roles throughout the lifetime of a synapse: during its initial assembly, recruitment of liprin-α could be mediated by any of multiple receptor pathways in a highly redundant manner. Once a synapse is formed, interactions between presynaptic CAMs and their specific postsynaptic ligands play more specialized roles in sculpting the molecular architecture and synapse-specific properties, probably under the influence of synaptic activity^5,13^.

Downstream of liprin-α recruitment by presynaptic CAMs, ELKS are required for both active zone formation and synaptic vesicles recruitment. This is in line with previous invertebrate studies^30,32,77^ and the ability of ELKS to capture and organize Rab6-coated vesicles^66,67^; but how the two processes interrelate remains incompletely understood. While it may seem intuitive that active zone assembly is a prerequisite for subsequent synaptic vesicle accumulations, the finding of normal vesicle pools in mice with severely impaired active zone assembly^24,78^ suggests that the two processes can be uncoupled. The ability of both ELKS and liprin-α to form phase-separated condensates clearly plays a role^44,62,65^, most notably demonstrated through elegant work in *C.* *elegans*^32^. This work showed not only that residues mediating LLPS in either ELKS or liprin-α are required for the recruitment of both active zone proteins and synaptic vesicles, but also confirmed causality by re-introducing an LLPS motif from an unrelated protein to rescue these defects. Our work confirms and further extends these findings by demonstrating a requirement of ELKS and liprin-α to interact in the same condensates. Interestingly, the ability of liprin-α to undergo LLPS is regulated by phosphorylation^32,44^, providing additional means to regulate presynapse assembly. However, in light of this work, it is somewhat surprising that any liprin-α1–4 isoform can confer similar levels of synaptic rescue in our system, given their strikingly different tendencies to form condensates in nonneuronal cells^44^. More studies will be needed to clarify how LLPS of liprin-α and ELKS contribute to the assembly of mammalian presynaptic terminals in physiological contexts.

Our finding that axonal transport of synaptic components is intact in qKO neurons may appear surprising in light of invertebrate papers describing reduced axonal transport in liprin-α/SYD-2 mutants^57,79,80^, but in line with findings that cargo mobility in cultured mouse neurons is unaffected by combined knockdown of liprin-α2 and -α3 (ref. ^56^). Instead, the identified possible interactions between liprin-α and KIF1A^55,57^ may serve to unload synaptic cargo at sites defined by liprin-α^56^.

Although neurotransmitter release was dramatically reduced (>97%) in qKO neurons, it is not completely eliminated. Close inspection of single cells revealed that 93% of all recorded qKO iGluts completely lacked synaptic activity, while the remaining 7% showed less than 2% residual synaptic activity compared with wild-type controls. These results indicate that liprin-α proteins are not essential for release per se, in line with the EM data demonstrating large but not complete reduction of synaptic vesicles in proximity to the PSD. Whether these vesicles account for the residual release observed in some qKO neurons is unknown, but appears plausible in light of recent evidence suggesting that even some ‘undocked’ synaptic vesicles can be primed and undergo release^24^.

Our work highlights the utility of compound genome editing combined with neuronal differentiation technologies in stem cells to systematically study the structure and function of human synapses within reasonable timescales. At the same time, our approach, like any other, has limitations. One of them might be that our in vitro studies cannot accurately recapitulate spatiotemporal developmental programs that probably play crucial roles during synapse formation in vivo. Moreover, our approach may not fully recapitulate mechanistic differences between synapse subtypes, commonly observed in vivo^11,13^. Future studies ablating liprin-α genes in mice, in different brain areas and at specific synapses, will probably address these issues. Regardless of the outcome of these experiments, future studies may capitalize on the model system presented here, for example, to study how the nanometer alignment of presynaptic release sites and postsynaptic receptors arise^10^. By selectively reconstructing specific synaptic molecular functions, facilitated by genetic manipulations of parental cell lines, the contribution of specific *trans*-synaptic CAM interactions and intracellular lattices formed by liprin-α and its partners to this fine-scale alignment should be assessed. Moreover, how complete liprin-α deletion directly or indirectly may affect postsynaptic functions should also be determined. Finally, as genetic variants in liprin-α3 have recently been identified in patients with developmental delay, intellectual disability, autism and epilepsy^50^, work aiming to assess how these variants impact the structure and function of human synapses to cause disease is warranted.

## Methods

### Cell culture

#### Maintenance of hES cells

Feeder-independent hES cells of line WA09/H9 (RRID: CVCL_9773; hPSCreg: WAe009-A) were obtained from WiCell and cultured on Matrigel-coated (15505739, Corning) dishes in mTeSR Plus medium (100-0276, StemCell Technologies). The medium was changed every other day and the cells passaged using ReLeaSR (05872, StemCell Technologies) every 3–5 days, depending on colony size. All cell cultures were maintained in a humidified incubator with 5% CO_2_ at 37 °C.

#### Maintenance of HEK cells and lentivirus production

Human embryonic kidney cells (HEK293T/17, American Type Culture Collection CRL-11268) were cultured at 37 °C with 5% CO_2_ in Dulbecco’s modified Eagle medium (DMEM)–GlutaMAX medium (31966047, Gibco) supplemented with 10% fetal bovine serum (FBS; F7524, Sigma). Medium was changed every 2 days and cells were split after reaching 70–80% confluence using trypsin-ethylenediaminetetraacetic acid (15400054, Gibco) or TrypLE (12605010, Gibco).

Lentiviruses were produced as described previously^81^, with slight modifications. HEK293 cells were seeded at 60% confluence and incubated 1 h before transfection with fresh medium supplemented with 25 μM chloroquine (C6628, Sigma). Cells were cotransfected using the calcium phosphate method with lentiviral helper plasmids as follows: 3.9 μg of pREV, 8.1 μg of pRRE, 6 μg of pVSVG and 12 μg of lentiviral vector DNA per 75 cm^2^ cell culture area. Medium was replaced again 2–3 h posttransfection. For constructs used on neurons, the medium was replaced with Neurobasal supplemented with 2% B27 (17504044, Gibco), GlutaMAX (35050061, Gibco) and 10 mM HEPES (15630080, Gibco). Lentiviruses were collected from the medium 40 h after transfection, pelleted by centrifugation at 1,500*g* for 10 min at 4 °C, aliquoted and frozen at −80 °C. For constructs used on ES cells to induce differentiation, medium replacement after transfection was done with fresh DMEM medium. Following collection and clearing, as described above, the lentiviral particles were pelleted by high-speed centrifugation (60,000*g* for 1.5 h), resuspended in MEM (51200046, Gibco) with 10 mM HEPES (100 μl per 30 ml of medium), aliquoted and snap frozen in liquid nitrogen.

#### Generation of iGluts

iGluts were generated from control (Ctrl1 and Ctrl2) and mutant (qKO1 and qKO2) ES cell clones according to previously described methods^45^. For each neuronal induction experiment, 250,000 hES cells were detached with Accutase (Gibco), plated on Matrigel-coated wells in mTeSR Plus containing Rho kinase inhibitor (Y27632, 1683, Axon Medchem, or Thiazovivin) and simultaneously transduced with lentiviruses FU–M2rtTA and Tet-O*–Ngn2–*puromycin. One day later (defined as DIV0), the medium was replaced with N2 medium (DMEM/F12; 11330032, Gibco), 1% N2 supplement (17502048, Gibco) 1% nonessential amino acids (11140050, Gibco), laminin (200 ng ml^−1^; 23017015, Thermo Fisher), brain-derived neurotrophic factor (BDNF) (10 ng ml^−1^; 450-02, Peprotech) and NT-3 (10 ng ml^−1^; 450-03, Peprotech) supplemented with doxycycline (2 μg ml^−1^, Alfa Aesar) to induce expression of *Ngn2* and the puromycin resistance cassette. The following day, puromycin (1 mg ml^−1^) was added to the medium. After 48 h of selection, cells were detached with Accutase (A1110501, Gibco) and replated on Matrigel-coated coverslips along with mouse glia (typically at a density of 150,000 iGluts per 24-well) in B27 medium (Neurobasal-A (12349015, Gibco) supplemented with B27 (17504044, Gibco), GlutaMAX (35050061, Gibco) laminin, BDNF and NT-3). Half of the medium was replaced every second day for 8 days, with cytosine arabinoside (ara-C; C6645, Sigma) added to a working concentration of 2 μM to prevent glia overgrowth. Experimental lentiviral constructs (for example, to express liprin-α rescue constructs) were added to the medium on day 4. From DIV10, neuronal growth medium (Neurobasal-A supplemented with B27, GlutaMAX and 5% FBS (SH30071.03HI, Hyclone)) was washed in and used for partial medium replacements every 3–4 days until analysis, typically after 4–6 weeks in culture.

In experiments aiming to assess evoked synaptic transmission (Fig. 3), the protocol for generation of iGluts was slightly different. Specifically, cells from each clone were further separated into two groups. In group 1, cells were infected with pFU–M2rtTA, pTet-O*–Ngn2*–puromycin and with lentiviruses expressing Channelrhodopsin oChiEF fused to tdTomato (termed here ChR–tdTomato)^82^. In group 2, cells were infected with pFU–M2rtTA, pTet-O–*Ngn2*–puromycin and lentiviruses to express nuclear-localized GFP (nGFP). Four days later, cells from groups 1 and 2 were washed three times with phosphate-buffered saline (PBS) to remove any lentivirus trace, detached and mixed at a ratio of 80%/20% (80% with ChR and 20% with nGFP), reseeded on Matrigel-coated coverslips along with mouse glia and cultured as described above. To record evoked synaptic transmission GFP + TdTomato cells were patched in whole-cell voltage clamp configuration and the presynaptic inputs onto patched cells activated with brief (5–10 ms) pulses of blue light (488 nm) using a light-emitting diode.

#### Generation of iGABAs

iGABAs were generated according to published protocols^46^. hES cells were treated with Accutase (Sigma), then plated and immediately infected lenti-rtTA, lenti-Ascl1, and exposed to doxycycline 1 day later to drive expression of Acsl1 and Dlx2. Two days later, puromycin and hygromycin (H3274, Sigma) were added to the medium during 24 h for selection. After four additional days of hygromycin selection, remaining cells were detached with Accutase and replated on Matrigel-coated coverslips along with mouse glia. Half of the medium was then changed every second day for 8 days and 2.5% FBS was added to support astrocyte viability. After DIV10, induced GABAergic neurons were cultured in B27/Neurobasal medium containing GlutaMAX (Gibco), 5% FBS and 10 ng ml^−1^ BDNF until performing analysis.

#### Generation of induced astrocytes

Induced astrocytes were generated following previously published methods^47^. Briefly, control and mutant ES cells were treated with Accutase (Sigma) and then seeded on Matrigel-coated 24-well plates at a density of 90,000 cells per well. Cells were maintained in mTeSR Plus medium supplemented with Y27632 (Axon Medchem). Cells were then transduced with lentiviruses FU–M2rtTA, Tet-O–Sox9–puromycin and Tet-O–Nfib–hygromycin and kept in DMEM/F12 medium containing 10% FBS, 1% N2 supplement and 1% GlutaMAX (expansion medium). One day later, 2.5 μg ml^−1^ of doxycycline (D9891, Sigma) was added to the medium to drive expression of *Sox9* and *NF1B*. Two days later, 1.25 μg ml^−1^ of puromycin and 200 μg ml^−1^ of hygromycin were added to the medium for selection. From day 3 onward, cells were kept in expansion medium, with the gradual addition of fibroblast growth factor (FGF) medium (Neurobasal-A medium supplemented with 2% B27, 1% nonessential amino acids, 1% GlutaMAX (all from Gibco) and 1% FBS (Sigma)), 8 ng ml^−1^ of FGF (100-18, Peprotech), 5 ng ml^−1^ of ciliary neurotrophic factor (450-13, Peprotech) and 10 ng ml^−1^ of BMP4 (120-05, Peprotech), with 2.5 μg ml^−1^ of doxycycline and 200 μg ml^−1^ of hygromycin, until expansion medium was completely replaced with FGF medium (also containing 2.5 μg ml^−1^ of doxycycline). Finally, on day 10, medium was replaced with B27-supplemented final medium (Neurobasal-A medium, 2% B27, 1% GlutaMAX and 5% FBS) containing 2.5 μg ml^−1^ of doxycycline. At day 21, induced astrocytes were detached and seeded along with induced glutamatergic neurons derived from Ctrl or qKO hES cells on Matrigel-coated coverslips.

#### Mouse glia cell isolation

Primary mouse glial cell culture was performed essentially as described previously^83^. Briefly, cortices from 0.5–2.5-day-old wild-type C57BL/6 mice of both sexes, housed under standard conditions in a 12/12 h light–dark cycle with food and water ad libitum, were dissected, pooled and triturated using a fire-polished Pasteur pipette followed by passage through a cell strainer. Cells were plated in flasks (two cortices/1× T75) precoated with poly-l-lysine (5 mg ml^−1^;P1274, Sigma) in DMEM supplemented with 10% FBS (Sigma). Upon reaching confluence, the glial cells were dissociated by trypsinization and reseeded twice to remove potential trace amounts of mouse neurons before the glia cell cultures were used for coculture with induced neurons. Animal procedures were approved by the Swedish Board of Agriculture, the Robert Koch Institute (Germany) and the ‘Regierungsprasidium’ Karlsruhe (Germany).

### Cloning of plasmid constructs

#### Lentiviral rescue constructs

Human full-length complementary DNA clones for *PPFIA1* (HsCD00460680) and *PPFIA3* (HsCD00341187) were obtained from the Harvard PlasmID repository, and *PPFIA2* (HsCD00877565) and *PPFIA4* (HsCD00946340) from DNASU. Full-length cDNA and truncation mutants were PCR-amplified using PrimeSTAR (R010A, Takara) and gel purified using the QIAEX II DNA purification kit (20051, Qiagen). Using the HiFi DNA assembly mix (E2621S, NEB), the amplicons were inserted in a lentiviral vector (‘pFU-’) downstream of the ubiquitin promoter and an N-terminal enhanced (E)GFP fusion (amplified from pEGFP–N1; Clontech). Correct clones were verified by Sanger sequencing and amplified using the Midiprep Plus kit (12945, Qiagen). Point mutants were generated using the QuickChange Site-Directed Mutagenesis kit (210518, Agilent Technologies).

#### Other constructs

All constructs were cloned by Gibson assembly as described above. pFU–Venus–ELKS1 and pFU–mScarlet–ELKS1 were cloned by fusing the cDNA of human ELKS1 (HsCD00860679; DNASU) downstream of mVenus or mScarlet, respectively. pFS–HA–PTPRS was generated from cDNA of human PTPRS (short isoform lacking meA, meB and FN4-7; NM_130853) with an intracellular myc-tag. The HA tag was placed in the N-terminus by replacing the endogenous signal peptide with that of Ig-kappa, followed by an HA tag. pCMV–LRRTM2–GFP was cloned by inserting the cDNA of LRRTM2 (HsCD00419164; PlasmID repository at Harvard Medical School) in the vector pEGFP_N1. pCMV–IL1RAPL1–GFP was cloned by inserting the cDNA of human IL1RAPL1 (HsCD00082647; DNASU) in the vector pEGFP_N1. pCMV–NGL3–GFP was cloned by inserting the cDNA of rat NGL3/Lrrc4b in the vector pEGFP_N1. pCMV–Nlgn1–Cherry and pCMV–TrkC–Cherry were generated using the insert of corresponding GFP-tagged constructs. The Nlgn1 construct contains the rat cDNA lacking the A and B splice inserts. For a summary of plasmids used in this study, see Supplementary Table 2.

### Gene editing of *PPFIA1–4*

#### sgRNA design and cloning

Exons to target were selected on the basis of the following criteria: (1) presence in all transcripts and (2) preferably containing a noninteger number of codons such that its full deletion would be expected to cause a frame shift. The design of single guide (sg)RNA sequences was aided by the CHOPCHOP design tool (v3)^84^. The following sgRNA sequences for *PPFIA1* and *2* were cloned into SpCas9(BB)-2A–GFP (PX458) and sgRNAs for *PPFIA3* and *PPFIA4* in LentiCRISPRv2 plasmids, as described previously^85,86^, with protospacer adjacent motif sequences in bold:

*PPFIA1* (exon 17): 5′-GTGCAGCCGGTCTAACCGAA **GGG**

*PPFIA2* (exon 20_1): 5′-TGTTGGCACTACCAAGCCCG **AGG**

*PPFIA2* (exon 20_2): 5′-TCTTCAATAGGACGTTTGTT **TGG**

*PPFIA3* (exon 11): 5′-TAAGCGGCTGTCCGAGACGG **TGG**

*PPFIA4* (exon 16): 5′-AGCGCGTCCCCACCACTCAG **CGG**

#### Gene editing of hES cells

Two liprin-α genes per electroporation experiment were simultaneously targeted by combining Cas9- and sgRNA-encoding plasmids containing either puromycin resistance (LentiCRISPRv2) or GFP (PX458) as selection markers. Cells at ~80% confluency were treated with 2 μM thiazovivin for 2 h before transfection and detached with Accutase. Per transfection, 500,000 cells were resuspended in solution P3 (V4XP-3032, Lonza), mixed with 1.5 μg of each plasmid and electroporated in a 16-Nucelocuvete strip using the 4D-Nucleofector system (Lonza) set at program CA-137. Immediately after completion of the pulse, cells were resuspended in 100 μl of equilibrated mTeSR Plus with thiazovivin and plated on Matrigel-coated 6-well plates. Cells were lifted 15 h posttransfection and GFP-positive cells sorted using a FACSAria III Flow Cytometer (BD) equipped with an automated cell deposition unit, using a 100 µm nozzle at 20 psi. Around 25,000 cells were sorted in bulk and plated on 2× wells of a 6-well plate. The medium was changed the next day and a 24 h period of puromycin selection (at 1 mg ml^−1^) was started (48 h posttransfection). Colonies were collected 1 week later for screening of mutant clones. A single clone with unambiguous null alleles in *PPFIA1* and *PPFIA4* was isolated and used for further targeting. After the simultaneous editing of *PPFIA2* and *PPFA3*, no clone with bi-allelic disruption of PPFIA2 could be obtained and the cells were thus subjected to a final round of editing with a new sgRNA toward the same PPFIA2 exon. This resulted in the isolation of clones qKO1 and qKO2.

#### Selection and screening of ES cells

Selected ES cells clones were initially screened for indels by PCR (HotStarTaq, Qiagen) followed by fragment analysis (‘IDAA’), essentially as described previously^87^. Selected clones were further analyzed by Sanger sequencing (Eurofins Genomics). For compound heterozygous clones, the PCR product was first cloned using the TOPO-TA kit (Thermo Fisher Scientific), to isolate allelic reads. Sanger traces were analyzed using Geneious Prime software and comparisons with the parental H9 line using the TIDE algorithm (v3.3.0; http://tide.nki.nl)^88^. The following primers were used: (PCR *PPFIA1* flanking exon 17) F: 5′-ATGCCGACCATCAGCGAAG-3′; R: 5′-TCTCTTTCCACTCGTGCTTGG-3′; (PCR *PPFIA2* flanking exon 20) F: 5′-GACTCACACTCTCCCTTCTTCC-3′; R: 5′-GTCTTCGATCCTTCTCAGCTTG-3′; (PCR *PPFIA3* flanking exon 11) F: 5′-GACCTTGCCCGAGATAGAGG-3′; R: 5′-ACCACTGCCAGCCACATAG-3′; (PCR *PPFIA4* flanking exon 16) F: 5′-CGGCATTGAGGGAAGAGTCT-3′; R: 5′-CACTGGGCAGGGTCATGA-3′.

### CRISPR and AAV genome editing

To generate an HA-tagged NRXN1 knock-in line, we used the ‘AAV-cTr’ vector, previously described elsewhere^54^. A simplified protocol was used to produce adeno-associated virus (AAV) particles. HEK293T/17 cells were cotransfected using calcium phosphate with the plasmid and AAV (serotype DJ) helper plasmids. After transfection, the medium was replaced to mTeSR Plus and incubated for 72 h. AAV particles were collected from the cleared conditioned medium supernatant, washed and concentrated using 15 ml Centrifugal Filter Units (Amicon Ultra-4 100 kDa molecular weight cutoff; Merck).

#### CRISPR targeting with RNP complexes

For the formation of ribonucleoprotein complexes (RNP), a synthetic sgRNA targeting the 3′-UTR of *NRXN1* (Integrated DNA Technologies) was incubated with Alt-R S.p. HiFi Cas9 Nuclease V3 (1081060, Integrated DNA Technologies) for 10 min at an equimolar sgRNA:Cas9 ratio in a concentration of 37 μM. The genomic sgRNA target sequence (with protospacer adjacent motif in bold) is 5′-TTGGGTTGGCTATAGAAAAG **AGG**. Briefly, 300,000 cells from control (Ctrl1) and mutant (qKO1) pretreated with thiazovivin were transfected with RNP complexes, as described above, and immediately infected with 4.5 μl of AAV supernatant expressing *NRXN1*–cTR-targeting vector as repair template. Targeted cells were selected with puromycin for 72 h and single-cell sorted by fluorescence-activated cell sorting for isolation of monoclonal lines.

### Transfection of iGlut cells

Transfection of iGluts for analysis of axonal transport was performed at DIV7 by calcium phosphate. Medium was removed and kept aside in a replicate plate at 37 °C. Cells were briefly washed with MEM, and CaPO_4_ precipitates were applied for a 25 min incubation period. Precipitates were prepared as follows: 1 μg of plasmid DNA, 2 μl of 2 M CaCl_2_ and sterile water to a final volume of 15 μl were vortex-dropwise added to 15 μl of 2× HBS buffer pH 7.05 (274 mM NaCl, 1.4 mM Na_2_HPO_4_, 10 mM KCl, 15 mM _D_-glucose and 42 mM HEPES). Crystals were removed by two washes with 1× Hank’s balanced salt solution buffer (without CaCl_2_/MgCl_2_; Gibco) and one wash with MEM (Gibco) before returning the cells to the original conditioned medium.

### Immunocytochemistry and SIM imaging

Cultured iGluts were fixed with prewarmed paraformaldehyde (PFA) solution (4% PFA and 4% sucrose in PBS, pH 7.4) for 15 min at room temperature (RT). Then, cells were washed three times in PBS (10 min each) and permeabilized with 0,1% Triton X-100 in PBS for exactly 10 min at RT. Blocking was performed for 1 h in a blocking buffer (2% goat serum, 1% bovine serum albumin and 0,01% NaN_3_ in PBS). Primary antibodies diluted in the blocking buffer were applied overnight at 4 degrees inside a humid chamber. Cells were then washed three times with PBS and fluorescent-labeled secondary antibodies were incubated for 1 h at RT. Finally, cells were washed three times in PBS and once in double-distilled H_2_O and mounted in microscope slides using ProLong Gold mounting medium (Thermo Fisher Scientific). For PSD95 staining, immunofluorescence was performed with the following modifications: neurons were maintained in culture for 52–55 days and fixed in ice-cold methanol fixing solution (90% methanol, 10% 2-(*N*-morpholino)ethanesulfonic acid buffer: 100 mM 2-(*N*-morpholino)ethanesulfonic acid pH 6.9, 1 mM ethylene glycol tetraacetic acid (EGTA) and 1 mM MgCl_2_) at RT for 5 min. Cells were washed three times in PBS and incubated in blocking–permeabilizing solution (2% goat serum, 1% bovine serum albumin, 0.01% NaN_3_ and 0.1% Triton X-100 in PBS) for 30 min, before proceeding with staining. The following primary antibodies were used (for details, see Supplementary Table 3): MAP2 (Encor, 1:1000), pan-synapsin (Proteogenix, 1:1,000), PSD95 (NeuroMab, 1:100 and Addgene 1:100 for SIM experiments), RIM1/2 (SySy, 1:200), Munc13-1 (SySy, 1:200), SV2 (DSHB, 1:500), bassoon (Sigma, 1:200), RIMBP-2 (SySy, 1:200), synaptophysin-1 (SySy, 1:200), CASK (Neuromab, 1:200), ERC1/2 (SySy, 1:200), piccolo (SySy, 1:200), CaV2.1 (SySy, 1:200), Tuj1 (Biolegend, 1:1,000) and HA (Biolegend, 1:200). For analysis of synaptic markers, cells were imaged using either a Nikon Eclipse Ti2 or a Leica SP8 confocal microscope, in both cases using a 60×/numerical aperture (NA) 1.4 oil immersion objective. Images were acquired, processed and analyzed with the experimenter blinded to the sample genotype/condition using either NIS Elements software or a custom ImageJ macro, respectively.

#### SR-SIM imaging

Images were acquired using an Elyra 7 microscope lattice SIM (Zeiss) with a plan-apochromat 63×/1.4 NA oil objective, controlled via ZEN black (3.0 SR, v16.0.17.306). Each image consisted of three image channels sequentially acquired in the following order: PSD95, MAP2 and neurexin-1–HA, labeled with secondaries 568, 405 and 488, respectively (to avoid photobleaching of 405 channel signal by 488 nm excitation). PSD95 and neurexin-1 channels were coregistered by using a reference sample (multicolor beads of size ~100 nm, subdiffraction-limited registration accuracy). MAP2 channel was excited at 405 nm (2.0%, 30 µW) and emission collected via a dual-color emission filter (BP420-480 + BP495-550, exposure 150 ms), neurexin-1 was excited at 488 nm (2.0%, 80 µW) and emission collected via a dual-color emission filter (BP420-480 + BP495-550, exposure 200 ms) and PSD95 was excited at 561 nm (1.5 %, 63 µW) and emission collected via a dual-color emission filter (BP570-620 + LP655, exposure: 150 ms). To minimize image shifts between channels, a single dichroic filter was used with a quadruple bandpass design (LBF 405/488/561/642). The SIM gratings used were 27.5, 27.5 and 32 µm for 405, 488 and 561 nm excitation, respectively. Lattice SIM three-dimensional processing for each channel independently was done using ZEN Black 3.0 SR software (v.16.0.17.306, Zeiss). *Z*-stack reconstruction and nanocluster analysis was performed using ImageJ/Fiji, with PSD95 and NRXN1 nanocluster size and number quantified using the SynapseEM plugin and MATLAB script.

### Heterologous synapse formation assay and receptor clustering assay

#### Heterologous synapse formation

HEK293T cells were plated at a confluency of 60% and transfected with plasmids expressing fluorescent recombinant postsynaptic receptors (mCherry–Nlgn1, mCherry–TrkC, pVenus–Nlgn1, YFP–TrkC, EGFP–NGL3 and YFP–ILRAPL1) with the calcium phosphate method as described above. pmCherry–N1 or pEGFP–N1 (Clontech) was transfected as negative controls. After 24 h, transfected HEK cells were collected with 0.5 mM ethylenediaminetetraacetic acid (15575-020, Thermo Fisher) in Dulbecco’s phosphate-buffered saline (14190144, Gibco), passed through a 35 μm cell strainer and plated in coculture with iN cells (DIV17) at a density of 20,000 per coverslip (200 cells μl^−1^). After 2 days, 48 h after coculture with neurons, DIV19 (72 h posttransfection), cells were fixed with 4% PFA/4% sucrose solution for 15 min at RT. Immunolabeling of presynaptic components was performed with the following antibodies: rabbit anti-pan-synapsin (E028 or nc30-1, 1:1,000), rabbit anti-piccolo (Sysy, 1:200), rabbit anti-bassoon (Sysy, 1:200), mouse anti-SV2 (DSHB, 1:500) or mouse anti-synaptophysin (Sysy, 1:200). The signal of EGFP–liprin-α proteins was enhanced by an anti-GFP antibody (DSHB, 1:500). Species-specific AlexaFluor 405 (1:1,000), AlexaFluor 488 (1:1,000), AlexaFluor 568 (1:1,000) and AlexaFluor 633 (1:600) secondaries (Supplementary Table 3) were used and samples mounted using Prolong gold (P36930, Thermo Fisher Scientific). Images were collected with a Nikon Eclipse Ti2 confocal microscope using a 40×/NA 1.15 water immersion objective. Quantification of presynaptic specialization analysis was performed by NIS elements AR software (v.5.21.01, Nikon Instruments). Normalized values of recruitment signal were assessed by quantifying the binary area of the markers recruited onto the surface of HEK293T cells per total area of HEK293T cells expressing fluorescent-tagged postsynaptic receptors. Background correction for the 633 channel was employed using a constant for HEK293T artificial synapse formation assays. All images were acquired and analyzed with the experimenter blinded to the sample genotype/condition.

#### Receptor clustering assay

HeLa cells (ATCC-CCL-2) were cultured in DMEM (Corning) supplemented with 10% FBS (Pan Biotech) and 50 U ml^−1^ penicillin and streptomycin. Transfections of indicated plasmids were performed with Lipofectamine 3000 (Thermo Fisher Scientific) according to the manufacturer’s instructions. One day after transfection, the cells were detached by trypsin treatment and subcultured onto ~20 μg ml^−1^ fibronectin (Millipore)-coated coverslips for additional 24 h. After fixation with 4% PFA, the cells were stained with indicated primary antibodies followed by fluorescent dye-conjugated secondary antibodies. Confocal images were acquired with a Nikon A1R confocal microscope. For immunofluorescence, primary antibodies against Flag (Sigma, 1:200 dilution) and HA (Cell Signaling, 1:200) were used. AlexaFluor 594-conjugated anti-mouse IgG or 647-conjugated anti-rabbit IgG was diluted 1:1,000. Images were analyzed using ImageJ.

### Electrophysiological recordings

#### General

On the day of recording, a coverslip containing induced neurons was placed in an RC-27 chamber (Sutter Instruments), mounted under a BX51 upright microscope (Olympus), equipped with differential interference contrast and fluorescent capabilities. Neurons were maintained at 26 ± 1 °C using a dual TC344B temperature control system (Sutter Instruments). Induced neurons were continuously perfused with oxygenated (95% O_2_/5% CO_2_) artificial cerebrospinal fluid solution containing (in mM): 125 NaCl, 2.5 KCl, 0.1 MgCl_2_, 4 CaCl_2_, 25 glucose, 1.25 NaH_2_PO_4_, 0.4 ascorbic acid, 3 myo-inositol, 2 Na-pyruvate and 25 NaHCO_3_, pH 7.4 and 315 mOsm. In a subset of experiments (Extended Data Figs. 1 and 2), the concentration of MgCl_2_ and CaCl_2_ was changed to 1 and 2 mM, respectively. Cells were approached and patched under differential interference contrast using 3.0 ± 0.5 MegaOhm glass pipettes (WPI), pulled with a PC10 puller (Narishige). Depending on the experimental configuration (see below), pipettes were filled with either voltage or current clamp internal solution containing (in mM) voltage clamp: 125 Cs–gluconate, 20 KCl, 4 MgATP, 10 Na–phosphocreatine, 0.3 GTP, 0.5 EGTA, 2 QX314 (HB1030, Hello Bio) and 10 HEPES–NaOH, pH 7.2, and current clamp 125 K-gluconate, 20 KCl, 10 HEPES, 0.5 EGTA, 4 ATP–magnesium, 0.3 GTP–sodium and 10 Na–phosphocreatine, osmolarity 312 mOsmol and pH 7.2, adjusted with KOH. For all experiments, a Multiclamp 700B amplifier (Axon Instruments) controlled by Clampex 10.1 and Digidata 1440 digitizer (Molecular Devices) were used. Detection and analysis of voltage and current clamp recordings were done with Clampfit 10.1 or with custom-written macros in Igor Pro 6.11. Electrophysiological recordings were done and analyzed with the experimenter blinded to the sample genotype/condition.

#### Current clamp recordings

In some experiments shown in Extended Data Fig. 1, whole-cell current clamp recordings from induced glutamatergic neurons were performed. In these experiments ~4 MΩ pipettes were used, and automatic bridge balance was performed after achieving whole-cell current clamp configuration. The membrane potential in all neurons was maintained at approximately −70 mV by injecting the appropriate feedback current into the cells. Current injections <50 pA were considered acceptable and those cells in which higher current injections were required were not included in the analysis.

#### Voltage clamp recordings

In most recordings (Figs. 3, 5 and 6), whole-cell voltage clamp was used. For recordings from induced glutamatergic neurons, membrane voltage was clamped at −70 mV, and miniature excitatory currents (recorded in the presence of 0.5 μM TTX; HB1035, Hello Bio) were detected as downward deflections. For recordings from induced GABAergic cells, membrane voltage was camped at 0 mV and inhibitory currents were recorded as upward deflections.

#### Evoked currents

In these experiments (Fig. 3), we recorded from GFP+/ChR− neurons (see above) in voltage clamp at −70 mV holding potentials, while simultaneously activating presynaptic inputs to recorded neurons with a single, short (5–20 ms) pulse of blue light (488), generated via a CoolLED illumination system (pE-300) controlled by a transistor–transistor logic pulse (Extended Data Fig. 4b).

#### Sucrose responses

In these experiments (Extended Data Fig. 2, and Figs. 5 and 6), cells were maintained at −70 mV holding potentials (voltage clamp configuration) and stimulated 0.5 M sucrose solution for 5 s. Sucrose solution was delivered in the vicinity of recorded cells (20–30 μm away), using a low-resistance glass pipette (1.5 MΩ), connected to a custom pressure device (5 psi).

### Time-lapse microscopy

#### Axonal transport

iN cells were plated on 35 mm four-compartment CellView dishes (627870, Greiner) at a cell density of 80,000 cells cm^−2^ and transfected at DIV6 with plasmids encoding mCherry and SV2–GFP, as described above. Imaging to assess axonal transport of vesicles and active zone components was performed at DIV13 and 19. Image acquisition was performed using a Nikon Eclipse Ti2 confocal equipped with a humidity- and CO_2_-controlled incubation chamber at 37 °C with a 40×/NA 1.15 water immersion objective. Images were obtained in fast-scan mode with an ~30 Hz frame rate for a total of 300 s per field of view. The resulting time-lapse movies were median filtered and background subtracted using the ‘detect local maximum’ function in the NIS elements AR software (v5.21.01, Nikon Instruments). Kymographs were generated and analyzed using the Multi Kymograph plugin of Fiji/ImageJ (v2.3.0/1.53f). Only moving puncta were analyzed and quantified.

#### Fluorescence recovery after photobleaching experiments

HeLa cells were plated on 35 mm four-compartment CellView dishes (Greiner) and transfected with the indicated liprin-α3 constructs using *Trans*IT-X2 (MIR6003, Mirus Bio). The next day, cells were treated with 2 μM phorbol 12-myristate 13-acetate (10 min before onset of imaging) and transferred to a Nikon Eclipse Ti2 confocal equipped with an humidity- and CO_2_-controlled incubation chamber at 28 °C. Images were obtained using a 40×/NA 1.15 water immersion objective at an ~1 Hz frame rate before and after photobleaching a small region of interest (ROI) containing a cytoplasmic condensate using the 405 nm laser at 100% power. The same ROIs were used to measure fluorescence over time, using NIS elements AR software. For colocalization experiments, HeLa cells cotransfected to express mScarlet-fused ELKS and the indicated liprin-α3 constructs were imaged live after treatment with 2 μM phorbol 12-myristate 13-acetate. Intensity profiles across representative images and the green–red Pearson correlation coefficient were analyzed using NIS elements AR software.

### Western blot

Protein samples were extracted from iN cultures at DIV19–22 lysed in radioimmunoprecipitation assay buffer (50 mM Tris pH 8.0, 150 mM NaCl, 0.1% sodium dodecyl sulfate, 0.5% sodium deoxycholate and 1% Triton X-100) supplemented with phenylmethylsulfonyl fluoride (36978, Thermo Fisher) and Complete Proteinase Inhibitor Cocktail (11873580001, Merck) for 20 min. Lysates were centrifuged at 20,000*g* for 10 min at 4 °C and supernatants containing solubilized proteins collected. Protein samples (30 µg each) in Laemmli buffer, reduced with dithiothreitol (0.1 mM, final concentration) were heated to 96 °C for 5 min and separated by sodium dodecyl sulfate–polyacrylamide gel electrophoresis in precast tris-glycine TGX gels (Bio-Rad). Transfer to a nitrocellulose membrane (Amersham) was performed in Towbin transfer buffer (25 mM tris, 0.2 M glycine and 20% methanol). Membranes were blocked with 5% nonfat milk (Aplichem) for 1 h and primary antibodies were incubated overnight at 4 °C. After washing the membranes three times with TBS-T (20 mM tris pH 7.5, 137 mM NaCl and 0.05% Tween-20), species-specific 680RD- or 800CW-conjugated secondary antibodies (LI-COR, all at 1:10,000 dilution) were incubated in 1:1 TBS-T Odyssey Blocking (927-50000, LI-COR) for 1 h. Membranes were imaged using an Odyssey CLx or DLx system (LI-COR). Immunoblotted bands were quantified by densitometry using Image Studio 5.2 software (LI-COR). Loading controls on the same membrane were used to normalize data. For quantitative comparisons, each measurement was normalized to the average value per blot, with the median value of controls set to 1. The following primary antibodies were used (for details, see Supplementary Table 3): liprin-α1, -α2, -α3 or -α4 (all used at 1:200 dilution), PTPRS (MediMabs, 1:1,000), neurexin-1 (Millipore, 1:1,000), RIM1 (SySy, 1:1,000), ELKS1/2 (SySy, 1:1,000), Munc13 (SySy, 1:1,000), RIMBP-2 (SySy, 1:1,000), CASK (Neuromab, 1:1,000), Nlgn1 (Neuromab, 1:500), Homer1 (SySy, 1:1,000) syntaxin-1 (SySy, 1:1,000), synapsin-2 (Sigma, 1:1,000), PSD95 (Thermo Fisher Scientific, 1:500), Veli123 (SySy, 1:1,000), ERC1/2 (SySy, 1:1,000), Mint-1 (SySy, 1:1,000), Rab3a (SySy, 1:1,000), SNAP25 (Sigma, 1:2,000), β-actin (Sigma, 1:1,000), synaptotagmin-1 (SySy, 1:1,000), Tuj1 (BioLegend, 1:5,000) and GFP (Thermo Fisher Scientific, 1:1,000).

### EM

Neurons grown on glass coverslips were fixed in Karnovsky fixative (2.5% glutaraldehyde, 2% formaldehyde and 0.02% NaN_3_ in 0.05 M cacodylate buffer) at 37 °C for 25 min. The samples were subsequently washed five times with 0.1 M cacodylate buffer for a total of 1 h. The fourth change of buffer contained 50 mM glycine (blocking residual aldehydes from fixative). Staining was performed with 1% osmium and 1% potassium ferrocyanide in 0.1 M cacodylate buffer, for 20 min at RT and washed with water three times. Tertiary staining was made with 2% uranyl acetate for 20 min at RT and after three more washes in water, samples were dehydrated with ethanol (4 min each of 30%, 50%, 70%, 85%, 90% and four times with 100% ethanol). Next, samples were infiltrated in Agar 100 resin (AGR1140, Agar Scientific), through a series of increasing concentration of resin (15 min each of 25%, 50% and 75% and three times with 100%). Embedding was performed with resin–benzyldimethylamine (Electron Microscopy Sciences) in BEEM capsules (TAAB Laboratories Equipment Ltd). Samples were polymerized for 48 h at 60 °C, and subsequently sectioned at 70 nm and mounted on noncoated copper grids (mesh size 150). Before imaging, sections were contrasted with Raynold’s lead citrate for 5 min. Images were acquired using a Talos L120C transmission electron microscope (Thermo Scientific). Subsequent image analysis was performed using ImageJ/Fiji (v2.3.0/1.53f).

#### Analysis of synaptic vesicle counts

Synaptic vesicles were defined, for the purpose of this analysis, as all spherical vesicles with a diameter <68 nm within 1,000 nm of a PSD-like structure. The number of total synaptic vesicles per bouton, diameter of synaptic vesicles, PSD length and distance of each synaptic vesicle to the active zone were analyzed using the SynapseEM ImageJ plugin and a MATLAB script, as described in ref. ^89^.

### Data analysis, statistics and reproducibility

Current and voltage clamp recordings were analyzed using Clampfit v10.2 (Molecular Devices) or written macros in Igor Pro v4.07 (WaveMetrics). Confocal images were handled and analyzed using NIS elements AR software (v5.21.01; Nikon Instruments), LASX (Leica) or ImageJ/Fiji (v2.3.0/1.53f) and numerical data processed in Excel (v16; Microsoft). EM and SIM images were analyzed using MATLAB (R2022a; MathWorks). Immunoblot images were handled and analyzed with Image Studio (v5.2; LI-COR). Sequence data were analyzed using Geneious Prime software (BioMatters). Stem cell work was performed in compliance with the German Stem Cell Act approved by the Robert Koch Institute.

Allocation (for example, the distribution of different experimental lentiviruses on separate coverslips, order of analysis and so on) was random. No statistical methods were used to predetermine sample sizes; the number of datapoints and independent repetitions was guided by previous studies ^54,78,81,83^. Results from multiple (typically three) independent experiments were performed, as indicated in figure legends and Supplementary Table 1, and the results were merged. Representative experiments were repeated at least once, except for screening PCRs (Extended Data Fig. 3e), the western blot confirming liprin-α1 deletion in human astrocytes (Extended Data Fig. 1c) and the liprin-α3 rescue condition for EM.

Summary data are shown as means ± s.e.m. Statistical analysis was performed using Prism 9 (GraphPad Software). Datasets were tested for normality (Gaussian distribution) using the D’Agostino Pearson test. For between-group comparisons, unpaired two-tailed *t*-tests were used if data distribution was normal, or two-tailed Mann–Whitney tests for non-Gaussian datasets. For multiple-group comparisons, statistical significance was determined by analysis of variance with Tukey’s or Holm–Šídák’s corrections for multiple comparisons, or Kruskal–Wallis followed by Dunn’s post hoc test for non-Gaussian datasets. ***P < 0.001, **P < 0.01 and *P < 0.05.

### Reporting summary

Further information on research design is available in the Nature Portfolio Reporting Summary linked to this article.

## Online content

Any methods, additional references, Nature Portfolio reporting summaries, source data, extended data, supplementary information, acknowledgements, peer review information; details of author contributions and competing interests; and statements of data and code availability are available at 10.1038/s41593-024-01592-9.

## Supplementary information

Supplementary Information Supplementary Tables 1–3.

Reporting Summary

## Source data

Source Data Fig. 1 Statistical source data.

Source Data Fig. 1 Unprocessed western blots.

Source Data Fig. 2 Statistical source data.

Source Data Fig. 3 Statistical source data.

Source Data Fig. 4 Statistical source data.

Source Data Fig. 5 Statistical source data.

Source Data Fig. 5 Unprocessed western blots.

Source Data Fig. 6 Statistical source data.

Source Data Extended Data Fig. 1 Statistical source data.

Source Data Extended Data Fig. 1 Unprocessed western blots.

Source Daa Extended Data Fig. 2 Statistical source data.

Source Data Extended Data Fig. 3 Statistical source data.

Source Data Extended Data Fig. 4 Statistical source data.

Source Data Extended Data Fig. 5 Statistical source data.

Source Data Extended Data Fig. 6 Statistical source data.

Source Data Extended Data Fig. 7 Statistical source data.

## Data Availability

Numerical source data are provided within this paper. Additional data that support the findings of this study are available from the corresponding authors upon reasonable request. Source data are provided with this paper.
