# Supplementary material for: Liprin-α proteins are master regulators of human presynapse assembly
Source: Nat Neurosci. 2024 Mar 12;27(4):629–42. doi: 10.1038/s41593-024-01592-9 (PMC11001580; doi:10.1038/s41593-024-01592-9)
Supplement: Supplementary file 1 — Supplementary Tables 1–3. [file 41593_2024_1592_MOESM1_ESM.pdf]

nature neuroscience

Article

https://doi.org/10.1038/s41593-024-01592-9

Liprin-α proteins are master regulators of human presynapse assembly

In the format provided by the authors and unedited

Supplementary Table 1 – Summary of statistical results

FIGURE / PANEL	SAMPLE SIZE	STATISTICS	RESULTS
Figure 1d<br>MAP2	$Ctrl_1$ 84 fields/3 batches<br>$Ctrl_2$ 94 fields/3 batches<br>qKO_1 94 fields/3 batches<br>qKO_2 80 fields/3 batches	Unpaired two-tailed Student's t-tests	$Ctrl_1$ vs. qKO1 $p<0.0001$<br>$Ctrl_2$ vs. qKO_2 $p<0.0001$
Figure 1e<br>Tuj1	$Ctrl_1$ 27 fields/3 batches<br>$Ctrl_2$ 17 fields/3 batches<br>qKO_1 20 fields/3 batches<br>qKO_2 21 fields/3 batches	Unpaired two-tailed Student's t-tests	$Ctrl_1$ vs. qKO_1 p= 0.2247, ns<br>$Ctrl_2$ vs. qKO_2 p= 0.6406, ns
Figure 1f<br>Protein levels	$Ctrl_1$ 3-7 batches<br>qKO_1 3-4 batches<br>$Ctrl_2$ 3-7 batches<br>qKO_2 3-4 batches	Two-way ANOVA with Holm-Šídák test for multiple comparisons, comparing $Ctrl_{1+2}$ vs. qKO_{1+2}	PTPRS: n=3+3 vs 3+3. adj. p = 0.9875<br>pan Nrxn: n=4+4 vs. 4+4. adj. p = 0.9001<br>CASK: n=5+3 vs. 5+3. adj. p = 0.8399<br>Nlgn1: n=5+3 vs. 5+3. adj. p = 0.9001<br>Homer: n=6+3 vs. 6+3. adj. p = 0.3618<br>PSD95: n=3+3 vs 3+3. adj. p = >0.9999<br>ERC 1/2: n=3+3 vs 3+3. adj. p = 0.934<br>RIM1: n=3+3 vs 3+3. adj. p = >0.9999<br>RIMBP2: n=4+4 vs. 4+4. adj. p = 0.1334<br>Munc13-1: n=5+5 vs. 5+5. adj. p = >0.9999<br>Mint1: n=6+3 vs. 6+3. adj. p = 0.4069<br>Veli 1: n=5+3 vs. 5+3. adj. p = 0.7908<br>Veli 2/3: n=5+3 vs. 5+3. adj. p = 0.9001<br>Synapsin2: n=7+4 vs. 7+4. adj. p = 0.3845<br>Syntaxin: n=4+3 vs. 4+3. adj. p = 0.8798<br>SNAP25: n=3+3 vs 3+3. adj. p = 0.967<br>Synaptotagmin1: n=6+3 vs. 6+3. adj. p = 0.0444<br>Rab3a: n=4+4 vs. 4+4. adj. p = 0.0179
Figure 1h	$Ctrl_1$ 24 fields/2 batches<br>$Ctrl_2$ 22 fields/2 batches<br>qKO_1 30 fields/2 batches<br>qKO_2 28 fields/2 batches	Two-tailed Mann-Whitney test	$Ctrl_1$ vs. qKO_1 $p < 0.0001$<br>$Ctrl_2$ vs. qKO_2 $p < 0.0001$
Figure 1i	qKO_1 58 cells/4 batches<br>qKO_1 +L1 61 cells/4 batches<br>qKO_1 +L2 66 cells/4 batches<br>qKO_1 +L3 64 cells/4 batches<br>qKO_1 +L4 32 cells/2 batches	Kruskal-Wallis test followed by Dunn's multiple comparisons test	qKO_1 vs. qKO1 + L1, $p < 0.001$<br>qKO_1 vs. qKO1 + L2, $p < 0.001$<br>qKO_1 vs. qKO1 + L3, $p < 0.001$<br>qKO_1 vs. qKO1 + L4, $p < 0.001$
Figure 1j	SV2:<br>$Ctrl_1$ 28 fields/3 batches<br>qKO_1 29 fields/3 batches<br>Synaptophysin-1:<br>$Ctrl_1$ 54 fields/2 batch<br>qKO_1 31 fields/2 batch<br>Piccolo:<br>$Ctrl_1$ 67 fields/2 batches<br>qKO_1 54 fields/2 batches<br>RIM1:<br>$Ctrl_1$ 30 fields/2 batches<br>qKO_1 24 fields/2 batches<br>RBP2:<br>$Ctrl_1$ 32 fields/2 batches<br>qKO_1 20 fields/2 batches<br>CaV2.1:<br>$Ctrl_1$ 31 fields/2 batches<br>qKO_1 26 fields/2 batches	Two-tailed Mann-Whitney tests	SV2 puncta:<br>qKO_1 vs. $Ctrl_1$, $p < 0.0001$<br>Synaptophysin-1 puncta:<br>qKO_1 vs. $Ctrl_1$, $p < 0.0001$<br>Piccolo puncta:<br>qKO_1 vs. $Ctrl_1$, $p < 0.0001$<br>RIM1 puncta:<br>qKO_1 vs. $Ctrl_1$, $p < 0.0001$<br>RBP2 puncta:<br>qKO_1 vs. $Ctrl_1$, $p < 0.0001$<br>CaV2.1 puncta:<br>qKO_1 vs. $Ctrl_1$, $p = 0.0001$
Figure 2b	$Ctrl_1$ 38 boutons (11 grids / 2 batches)<br>qKO_1 49 boutons (11 grids / 2 batches)<br>qKO_1+L3 37 boutons (11 grids /1 batch)	Kruskal-Wallis test followed by Dunn's multiple comparisons test	Vesicles per bouton:<br>qKO_1 vs. $Ctrl_1$ adj. $p < 0.0001$<br>qKO_1 vs. qKO_1+L3 adj. $p = 0.0012$
Figure 2c	Total number of vesicles analyzed:<br>$Ctrl_1$ 3020 vesicles / 50 active zones<br>qKO_1 602 vesicles / 50 active zones<br>qKO_1+L 1338 vesicles / 41 active zones	(no statistical comparisons made)	

Figure 2f	$Ctrl_{1\ Nrxn\text{-}HA}$ +Cre 10 cover slips / 2 batches<br>$qKO_{1\ Nrxn\text{-}HA}$ +Cre 9 cover slips / 2 batches<br>$Ctrl_{1\ Nrxn\text{-}HA}$ no cre virus 4 cover slips / 2 batches<br>$qKO_{1\ Nrxn\text{-}HA}$ no cre virus 2 cover slips / 2 batches	(no statistical comparisons made)	
Figure 2h-i	PSD length:<br>Ctrl1 $_{Nrxn\text{-}HA}$ +Cre 32 boutons /1 batch<br>$qKO_{1\ Nrxn\text{-}HA}$ +Cre 25 boutons/1 batch<br>NRXN puncta size:<br>Ctrl1 $_{Nrxn\text{-}HA}$ +Cre 56 PSDs /1 batch<br>$qKO_{1\ Nrxn\text{-}HA}$ +Cre 41 PSDs /1 batch<br>Nanoclusters per PSD<br>Ctrl1 $_{Nrxn\text{-}HA}$ +Cre 32 boutons /1 batch<br>$qKO_{1\ Nrxn\text{-}HA}$ +Cre 25 boutons/1 batch	(no statistical comparisons made)	
Figure 3b<br>mEPSCs	$Ctrl_1$ 49 cells/3 batches<br>qKO_1 49 cells/3 batches<br>$Ctrl_2$ 33 cells/3 batches<br>qKO_2 34 cells/3 batches	Two-tailed Mann-Whitney test	$Ctrl_1$ vs. qKO_1 $p < 0.0001$<br>$Ctrl_2$ vs. qKO_2 $p < 0.0001$
Figure 3d<br>Evoked EPSCs	$Ctrl_1$ 36 cells/2 batches<br>qKO_1 35 cells/2 batches<br>$Ctrl_1$ 35 cells/2 batches<br>qKO_1 35 cells/2 batches	Two-tailed Mann-Whitney test	$Ctrl_1$ vs. qKO_1 $p < 0.0001$<br>$Ctrl_2$ vs. qKO_2 $p < 0.0001$
Figure 3f<br>mIPSCs	$Ctrl_1$ 41 cells/2 batches<br>qKO_1 41 cells/2 batches<br>$Ctrl_2$ 59 cells/3 batches<br>qKO_2 61 cells/3 batches	Two-tailed Mann-Whitney test	$Ctrl_1$ vs. qKO_1 $p < 0.0001$<br>$Ctrl_2$ vs. qKO_2 $p < 0.0001$
Figure 3h<br>mEPSC Rescues	qKO_1 60 cells/3 batches<br>qKO_1+L1 70 cells/5 batches<br>qKO_1+L2 70 cells/5 batches<br>qKO_1+L3 65 cells/5 batches<br>qKO_1+L4 25 cells/2 batches	One-way ANOVA followed by a Bonferroni test for multiple comparisons	qKO_1 vs. qKO_1 + L1 $p < 0.0001$<br>qKO_1 vs. qKO_1 + L2 $p < 0.0001$<br>qKO_1 vs. qKO_1 + L3 $p < 0.0001$<br>qKO_1 vs. qKO_1 + L4 $p < 0.0001$
Figure 4a<br>SV2 transport	$Ctrl_1$ 14 fields/2 batches<br>qKO_1 13 fields/2 batches	Two-tailed Student's t-tests	Velocity:<br>qKO_1 vs. $Ctrl_1$, $p = 0.54$<br>Movement events:<br>qKO_1 vs. $Ctrl_1$, $p = 0.03$
Figure 4b<br>ELKS transport	$Ctrl_1$ 10 fields/3 batches<br>qKO_1 12 fields/3 batches	Two-tailed Student's t-tests	Velocity:<br>qKO_1 vs. $Ctrl_1$, $p = 0.55$<br>Movement events:<br>qKO_1 vs. $Ctrl_1$, $p = 0.33$
Figure 4c<br>CASK recruitment	$Ctrl_1$ 158 cells /4 batches<br>qKO_1 119 cells /4 batches	Two-tailed Mann-Whitney test	$Ctrl_1$ vs. qKO_1 $p < 0.0001$
Figure 4d<br>ELKS recruitment	$Ctrl_1$ 126 cells/3 batches<br>qKO_1 94 cells/3 batches	Two-tailed Mann-Whitney test	$Ctrl_1$ vs. qKO_1 $p < 0.0001$
Figure 4e<br>RIM recruitment	$Ctrl_1$ 123 cells/3 batches<br>qKO_1 96 cells/3 batches	Two-tailed Mann-Whitney test	$Ctrl_1$ vs. qKO_1 $p < 0.0001$
Figure 4f<br>Piccolo recruitment	$Ctrl_1$ 143 cells/3 batches<br>qKO_1 79 cells/3 batches	Two-tailed Mann-Whitney test	$Ctrl_1$ vs. qKO_1 $p < 0.0001$
Figure 4g<br>CaV2.1 recruitment	$Ctrl_1$ 98 cells/3 batches<br>qKO_1 86 cells/3 batches	Two-tailed Mann-Whitney test	$Ctrl_1$ vs. qKO_1 $p < 0.0001$
Figure 5b<br>Recruitment ASF	qKO_1+L3 75 HEK cells/1 batch<br>qKO_1+L3ΔCC 52 HEK cells /1 batch<br>qKO_1+L3ΔRIMBD 127 HEK cells /1 batch<br>qKO_1+L3ΔELKSBD 97 HEK cells /1 batch<br>qKO_1+L3ΔSAM 50 HEK cells /1 batch<br>qKO_1+L3ΔLoop 67 HEK cells /1 batch<br>qKO_1+L3W921A 64 HEK cells /1 batch<br>qKO_1+L3W856Q 71 HEK cells /1 batch	(no statistical comparisons made)	
Figure 5c<br>Synapsin Puncta	qKO_1 + L3 35 cells/2 batches<br>qKO_1 + L3 ΔCC 22 cells/2 batches<br>qKO_1 + L3 ΔRIMBD 25 cells/2 batches<br>qKO_1 + L3 ΔELKSBD 29 cells/2 batches<br>qKO_1 + L3 ΔSAM 24 cells/2 batches<br>qKO_1 + L3 ΔLoop 26 cells/2 batches<br>qKO_1 + L3 W971A ΔCASK 26 cells/2 batches<br>qKO_1 + L3 W856Q 30 cells/2 batches	Kruskal-Wallis test followed by Dunn's multiple comparisons test	L3 ΔCC vs. L3 ΔRIM-BD, $p < 0.0001$<br>L3 ΔCC vs. L3 ΔELKS-BD, $p=0.1064$<br><br>L3 ΔSAM vs. L3 ΔLoop, $p= 0.0011$<br>L3 ΔSAM vs. L3 W921A, $p= 0.0189$<br>L3 ΔSAM vs. L3 W856Q, $p= 0.3314$
Figure 5d<br>mEPSC	qKO_1 + L3 66 cells/3 batches<br>qKO_1 + L3 ΔCC 59 cells/3 batches<br>qKO_1 + L3 ΔRIMBD 67 cells/3 batches	Kruskal-Wallis test followed by Dunn's	L3 ΔCC vs. L3 ΔRIM-BD, $p < 0.0001$<br>L3 ΔCC vs. L3 ΔELKS-BD, $p=0.1375$

	qKO$_1$ + L3 ΔELKSBD 71 cells/3 batches<br>qKO$_1$ + L3 ΔSAM 70 cells/3 batches<br>qKO$_1$ + L3 ΔLoop 68 cells/3 batches<br>qKO$_1$ + L3 W931A ΔCASK 71 cells/3 batches<br>qKO$_1$ + L3 W856Q 68 cells/3 batches	multiple comparisons test	L3 ΔSAM vs. L3 ΔLoop, p < 0.0001<br>L3 ΔSAM vs. L3 W921A, p < 0.0001<br>L3 ΔSAM vs. L3 W856Q, p < 0.0001
Figure 5e<br>Sucrose	qKO$_1$ + L3 33 cells/3 batches<br>qKO$_1$ + L3 ΔCC 27 cells/3 batches<br>qKO$_1$ + L3 ΔRIMBD 33 cells/3 batches<br>qKO$_1$ + L3 ΔELKSBD 48 cells/3 batches<br>qKO$_1$ + L3 ΔSAM123 30 cells/3 batches<br>qKO$_1$ + L3 ΔLoop 32 cells/3 batches<br>qKO$_1$ + L3 W971A ΔCASK 32 cells/3 batches<br>qKO$_1$ + L3 W856Q 41 cells/3 batches	Kruskal-Wallis test followed by Dunn's multiple comparisons test	L3 ΔCC vs. L3 ΔRIM-BD, p < 0.0001<br>L3 ΔCC vs. L3 ΔELKS-BD, p=0.3405<br><br>L3 ΔSAM vs. L3 ΔLoop, p < 0.0001<br>L3 ΔSAM vs. L3 W921A, p < 0.0001<br>L3 ΔSAM vs. L3 W856Q, p=0.0103
Figure 6b<br>QA recruitment	Ctrl$_1$ + L3 73 HEK cells/2 batches<br>Ctrl$_1$ + QA 188 HEK cells/3 batches<br>qKO$_1$ + L3 78 HEK cells/3 batches<br>qKO$_1$ + QA 156 HEK cells/3 batches	Two-tailed Mann-Whitney test	qKO$_1$ + L3 vs. qKO1 + QA p < 0.0001
Figure 6c<br>Synapsin puncta QA rescue	qKO$_1$ 26 cells/2 batches<br>qKO$_1$ + L3 32 cells/2 batches<br>qKO$_1$ + L3W921A; W856Q 34 cells/2 batches	Kruskal-Wallis test followed by Dunn's multiple comparisons test	qKO1+L3 vs. qKO1+L3 QA, p < 0.0001<br>qKO1 vs. qKO1+L3 QA, p = 0.8373
Figure 6d<br>mEPSCs QA rescue	qKO$_1$ 67 cells/5 batches<br>qKO$_1$ + L3 49 cells/5 batches<br>qKO$_1$ + L3 QA 83 Cells/5 batches	Kruskal-Wallis test followed by Dunn's multiple comparisons test	qKO1+L3 vs. qKO1+L3 QA, p < 0.0001<br>qKO1 vs. qKO1+L3 QA, p = 0.107
Figure 6e<br>Sucrose QA mutant	qKO$_1$ 35 cells/2 batches<br>qKO$_1$ + L3 40 cells/2 batches<br>qKO$_1$ + L3 QA 33 cells/2 batches	Kruskal-Wallis test followed by Dunn's multiple comparisons test	qKO1+L3 vs. qKO1+L3 QA, p = 0.0002<br>qKO1 vs. qKO1+L3 QA, p > 0.9999
Figure 6f<br>Active zone markers	ELKS signal:<br>qKO$_1$ 26 ROIs/2 batches<br>qKO$_1$ + L3 33 ROIs/2 batches<br>qKO$_1$ + L3 QA 16 ROIs/2 batches<br>RIM1 signal:<br>qKO$_1$ 26 ROIs/2 batches<br>qKO$_1$ + L3 40 ROIs/2 batches<br>qKO$_1$ + L3 QA 34 ROIs/2 batches<br>Piccolo signal:<br>qKO$_1$ 22 ROIs/2 batches<br>qKO$_1$ + L3 29 ROIs/2 batches<br>qKO$_1$ + L3 QA 22 ROIs/2 batches	Kruskal-Wallis test followed by Dunn's multiple comparisons test	ELKS signal:<br>qKO$_1$ vs. qKO$_1$+L3 QA, p =0.5314<br>qKO$_1$+L3 vs. qKO$_1$+L3 QA, p< 0.0001<br><br>RIM1 signal:<br>qKO$_1$ vs. qKO$_1$+L3 QA, p =0.0432<br>qKO$_1$+L3 vs. qKO$_1$+L3 QA, p< 0.0001<br><br>Piccolo signal:<br>qKO$_1$ vs. qKO$_1$+L3 QA, p =0.0896<br>qKO$_1$+L3 vs. qKO$_1$+L3 QA, p< 0.0001
Ext Data Figure 1d<br>Conversion	Ctrl$_1$ 20 batches<br>Ctrl$_2$ 10 batches<br>qKO$_1$ 20 batches<br>qKO$_2$ 11 batches	Two-tailed Mann-Whitney test	Ctrl$_1$ vs. qKO$_1$ p = 0.6157, ns<br><br>Ctrl$_2$ vs. qKO$_2$ p = 0.6047, ns
Ext Data Figure 1e<br>AP properties	Ctrl$_1$ 24 Cells/2 batches<br>qKO$_1$ 23 Cells/2 batches<br>Ctrl$_2$ 22 Cells/2 batches<br>qKO$_2$ 23 Cells/2 batches	Two-tailed Mann-Whitney test	AP amplitude<br>Ctrl$_1$ vs. qKO$_1$ p = 0.1835<br>Ctrl$_2$ vs. qKO$_2$ p = 0.1507<br>AP half-width<br>Ctrl$_1$ vs. qKO$_1$ p = 0.8043<br>Ctrl$_2$ vs. qKO$_2$ p = 0.2016<br>AP max speed<br>Ctrl$_1$ vs. qKO$_1$ p = 0.1632<br>Ctrl$_2$ vs. qKO$_2$ p = 0.7401
Ext Data Figure 1g<br>Rheobase and Capacitance	Rheobase:<br>Ctrl$_1$ 40 cells/3 batches<br>qKO$_1$ 37 cells/3 batches<br>Ctrl$_2$ 31 cells/3 batches<br>qKO$_2$ 40 cells/3 batches<br><br>Capacitance:<br>Ctrl$_1$ 45 cells/3 batches<br>qKO$_1$ 40 cells/3 batches<br>Ctrl$_2$ 30 cells/3 batches<br>qKO$_2$ 31 cells/3 batches	Unpaired two-tailed Student's t-test with Welch's correction	Rheobase:<br>Ctrl$_1$ vs. qKO$_1$ p<0.0001<br><br>Ctrl$_2$ vs. qKO$_2$ p<0.0028<br><br>Capacitance:<br>Ctrl$_1$ vs. qKO$_1$ p<0.0001<br><br>Ctrl$_2$ vs. qKO$_2$ p<0.0001
Ext Data Figure 2b	Ctrl$_1$ 1035 vesicles / 50 active zones<br>qKO1 202 vesicles / 50 active zones<br>qKO$_1$+L3 505 vesicles / 43 active zones	(no statistical comparisons made)	

Ext Data Figure 2c	Vesicle size:<br>$Ctrl_1$ 3124 vesicles<br>qKO_1 732 vesicles<br>qKO_1+L3 1460 vesicles<br><br>PSD length:<br>$Ctrl_1$ 49 PSDs / 2 batches<br>qKO1 53 PDSs / 2 batches<br>qKO_1+L3 41 PSDs / 1 batch	Kruskal-Wallis test followed by Dunn's multiple comparisons test	Vesicle size:<br>qKO_1 vs. $Ctrl_1$ adj. p = 0.8129, ns<br>qKO_1 vs. qKO_1+L3 adj. p > 0.9999, ns<br><br>PSD length:<br>qKO_1 vs. $Ctrl_1$ adj. p = 0.7933, ns<br>qKO_1 vs. qKO_1+L3 adj. p > 0.9999, ns
Ext Data Figure 2d Sucrose response	$Ctrl_1$ 48 cells/3 batches<br>qKO_1 57 cells/3 batches	Two-tailed Mann-Whitney test	qKO_1 vs. $Ctrl_1$, p<0.0001
Ext Data Figure 3a Artificial synapse formation	GFP:<br>$Ctrl_1$ 70 HEK cells/1 batch<br>qKO_1 59 HEK cells/1 batch<br>NLGN1:<br>$Ctrl_1$ 76 HEK cells/1 batch<br>qKO_1 77 HEK cells/1 batch<br>LRRTM2:<br>$Ctrl_1$ 86 HEK cells/1 batch<br>qKO_1 80 HEK cells/1 batch<br>TrkC:<br>$Ctrl_1$ 93 HEK cells/1 batch<br>qKO_1 69 HEK cells/1 batch<br>ILRAPL1:<br>$Ctrl_1$ 130 HEK cells/1 batch<br>qKO_1 122 HEK cells/1 batch<br>NGL3:<br>$Ctrl_1$ 84 HEK cells/1 batch<br>qKO_1 44 HEK cells/1 batch	Kruskal-Wallis test followed by Dunn's multiple comparisons test	GFP:<br>qKO_1 vs. $Ctrl_1$ ns, adj. p > 0.999<br><br>NLGN1:<br>qKO1 vs. $Ctrl_1$, adj. p < 0.0001<br><br>LRRTM2<br>qKO_1 vs. $Ctrl_1$, adj. p < 0.0001<br><br>TrkC<br>qKO_1 vs. $Ctrl_1$, adj. p < 0.0001<br><br>ILRAPL1<br>qKO_1 vs. $Ctrl_1$, adj. p < 0.0001<br><br>NGL3<br>qKO_1 vs. $Ctrl_1$, adj. p < 0.0001
Ext Data Figure 3b Nrxn1α-HA overexpressed	Nrxn-HA Nlgn1:<br>$Ctrl_1$ 164 HEK cells/4 batches<br>qKO_1 45 HEK cells/2 batches<br>Nrxn-HA TrkC:<br>$Ctrl_1$ 43 HEK cells /2 batches<br>qKO_1 23 HEK cells /2 batches	Kruskal-Wallis test followed by Dunn's multiple comparisons test	qKO_1 + $Nlgn_1$ vs. qKO_1+TrkC p < 0.0001
Ext Data Figure 3c PTPσ-HA overexpressed	PTPRS-HA Nlgn1:<br>$Ctrl_1$ 161 HEK cells /4 batches<br>qKO_1 85 HEK cells /4 batches<br>PTPRS-HA TrkC:<br>$Ctrl_1$ 80 HEK cells /3 batches<br>qKO_1 90 HEK cells /3 batches	Kruskal-Wallis test followed by Dunn's multiple comparisons test	qKO_1+TrkC vs. qKO_1+Nlgn1 p < 0.0001
Ext Data Figure 3f	$Ctrl_{1\ Nrxn\text{-}HA}$ +Cre 281 dendrites/2 batches<br>$qKO_{1\ Nrxn\text{-}HA}$ +Cre 240 dendrites/2 batches<br>$Ctrl_{1\ Nrxn\text{-}HA}$ -Cre 126 dendrites/2 batches<br>$qKO_{1\ Nrxn\text{-}HA}$ -Cre 86 dendrites/2 batches	(no statistical comparisons made)	
Ext Data Figure 3g NRXN1-HA endogenous	Nlgn1:<br>$Ctrl_1$ 239 HEK cells /3 batches<br>qKO_1 233 HEK cells /3 batches<br>TrkC:<br>$Ctrl_1$ 107 HEK cells /2 batches<br>qKO_1 90 HEK cells /2 batches	Kruskal-Wallis test followed by Dunn's multiple comparisons test	qKO_1 NrxnHA+Nlgn1 vs.<br>qKO_1 NrxnHA+TrkC adj. p < 0.0001
Ext Data Figure 4a mEPSCs (4 mM Ca)	$Ctrl_1$ 51 cells/3 batches<br>qKO_1 55 cells/3 batches<br>$Ctrl_2$ 40 cells/3 batches<br>qKO_2 37 cells/3 batches	Two-tailed Mann-Whitney test	$Ctrl_1$ vs. qKO_1 p < 0.0001<br>$Ctrl_2$ vs. qKO_2 p < 0.0001
Ext Data Figure 4c iGABA Synapsin puncta	$Ctrl_1$ 25 cells/1 batch<br>qKO_1 26 cells/1 batch<br>$Ctrl_2$ 36 cells/2 batches<br>qKO_2 35 cells/2 batches	Unpaired two-tailed Student's t-tests	$Ctrl_1$ vs. qKO_1 p < 0.0001<br><br>$Ctrl_2$ vs. qKO_2 p < 0.0001
Ext Data Figure 5a Munc13 recruitment	$Ctrl_1$ 101 HEK cells/3 batches<br>qKO_1 80 HEK cells/3 batches	Two-tailed Mann-Whitney test	$Ctrl_1$ vs. qKO_1 p < 0.0001
Ext Data Figure 5b RIMBP recruitment	$Ctrl_1$ 78 HEK cells/3 batches<br>qKO_1 69 HEK cells/3 batches	Two-tailed Mann-Whitney test	$Ctrl_1$ vs. qKO_1 p < 0.0001
Ext Data Figure 5c Bassoon recruitment	$Ctrl_1$ 108 HEK cells/3 batches<br>qKO_1 99 HEK cells/3 batches	Two-tailed Mann-Whitney test	$Ctrl_1$ vs. qKO_1 p < 0.0001

Ext Data Figure 6b<br>Pearson co-localization	GFP-Liprin-α3 + Scarlet-ELKS 56 cells/4 batches<br>GFP-ΔELKSBD + Scarlet-ELKS 46 cells/4 batches	Two-tailed Mann-Whitney test	L3 vs ΔELKSBD, $p < 0.001$
Ext Data Figure 6c<br>Active zone markers	ELKS signal:<br>qKO_1 26 ROIs/2 batches<br>qKO_1 + L3 33 ROIs/2 batches<br>qKO_1 + L3 ΔELKSBD 31 ROIs/2 batches<br><br>RIM1 signal:<br>qKO_1 26 ROIs/2 batches<br>qKO_1 + L3 40 ROIs/2 batches<br>qKO_1 + L3 ΔELKSBD 33 ROIs/2 batches<br><br>Piccolo signal:<br>qKO_1 22 ROIs/2 batches<br>qKO_1 + L3 29 ROIs/2 batches<br>qKO_1 + L3 ΔELKSBD 26 ROIs/2 batches	One-way ANOVA followed by a Tukey's multiple comparison test	ELKS signal:<br>qKO_1 vs. qKO_1+L3 ΔELKSBD,<br>$p = 0.2357$<br>qKO_1+L3 vs. qKO_1+L3 ΔELKSBD,<br>$p < 0.0001$<br>RIM1 signal:<br>qKO_1 vs. qKO_1+L3 ΔELKSBD,<br>$p = 0.0417$<br>qKO_1+L3 vs. qKO_1+L3 ΔELKSBD,<br>$p < 0.0001$<br>Piccolo signal:<br>qKO_1 vs. qKO_1+L3 ΔELKSBD,<br>$p = 0.3073$<br>qKO_1+L3 vs. qKO_1+L3 ΔELKSBD,<br>$p < 0.0001$
Ext Data Figure 7a	L3 + PTPσ 30 cells/3 batches<br>L3 W856Q + PTPσ 30 cells/3 batches	Unpaired two-tailed Student t-test	L3 vs. L3 Q856Q $p < 0.0001$
Ext Data Figure 7b	L3 + Nrxn1α 30 cells/3 batches<br>L3 + CASK + Nrxn1α 30 cells/3 batches<br>L3 W921A + CASK + Nrxn1α 30 cells/3 batches	One-way ANOVA with Holm-Šídák test for multiple comparisons	L3 + Nrxn1α vs.<br>L3 + CASK + Nrxn1α $p = 0.0014$<br>L3 + CASK + Nrxn1α vs.<br>L3 W921A + CASK + Nrxn1α $p = 0.0040$

Supplementary Table 2 – Plasmids

PLASMID	SOURCE
pSpCas9(BB)-2A-GFP (PX458)	RRID: Addgene_48138
LentiCRISPR-v2	RRID: Addgene_52961
PX458-sgRNA-PPFIA1_exon17	This paper
PX458-sgRNA-PPFIA2_exon20	This paper
LentiCRISPR-v2-sgRNA-PPFIA3_exon11	This paper
LentiCRISPR-v2-sgRNA-PPFIA4_exon16	This paper
pFU-EGFP-Liprin-α1	This paper
pFU-EGFP-Liprin-α2	This paper
pFU-EGFP-Liprin-α3	This paper
pFU-EGFP-Liprin-α4	This paper
pFU-EGFP-PPFIA3 (W921A)	This paper
pFU-EGFP-Liprin-α3 (ΔLoop)	This paper
pFU-EGFP-Liprin-α3 (ΔSAM123)	This paper
pFU-EGFP-Liprin-α3 (W856Q)	This paper
pFU-EGFP-Liprin-α3 (W856Q/W921A) 'QA'	This paper
pFU-EGFP-Liprin-α3 (ΔRIMBD)	This paper
pFU-EGFP-Liprin-α3 (ΔELKSBD)	This paper
pFU-EGFP- Liprin-α3 (ΔCoiled-coil)	This paper
pFS-Nrx1α-HA (based on NM_001403321)	Ref [2]
pCMV-Nrx1α-HA (based on NM_001403321)	Ref [2]
pFS-HA-PTPRS (based on NM_130853)	This paper
pCMV-FLAG-PTPRS (based on NM_130853)	This paper
pFU-EGFP-SV2	Ref [3]
pFU-Venus-ELKS1	This paper
pFU-mScarlet-ELKS1	This paper
pmCherry-N1	Clontech Laboratories # 632523
pCMV-Nlgn1[-A -B]-Cherry	This paper
pCMV-TrkC-Cherry	This paper
pEGFP-N1	Clontech Laboratories #6085-1
pCMV-Nlgn1[-A -B]-Venus	Ref [4]
pCMV-TrkC-YFP	Ref [2]
pCMV-LRRTM2-GFP	This paper
pCMV-IL1RAPL1-GFP	This paper
pCMV-NGL3-GFP	This paper
pFU-NLS-GFP-Cre	This paper

pAAV-NRXN1-cTr	Ref [5]
pRC-DJ for AAV	Ref [6]
pHelper for AAV	Ref [6]
pAAV-MCS	RRID: Addgene_46954
pMDLg/pRRE	RRID: Addgene_12251
pRSV-REV	RRID: Addgene_12253
pVSVG	RRID: Addgene_35616
pFU-M2rtTA	RRID: Addgene_20342
pTet-O-Ngn2-puromycin	RRID: Addgene_52047
pTet-O-AScl1-puromycin	RRID: Addgene_97329
pTet-O-Dlx2-hygromycin	RRID: Addgene_97330
pTet-O-Sox9-puromycin	RRID: Addgene_117269
pTet-O-Nfib-hygromycin	RRID: Addgene_117271
pFU-oChIEF-tdTomato	This paper
pFU-NLS-EGFP	This paper

Supplementary Table 3 – Antibodies

ANTIBODY	SOURCE	IDENTIFIER / RRID	USE/DILUTION<br>WB, western blot<br>ICC, immunocytochemistry
Mouse anti-β-actin	Sigma	Cat #: A5441<br>RRID: AB_476744	WB: 1:1000
Rabbit anti-Bassoon	Sigma	Cat #: SAB5200101	ICC: 1:200
Mouse anti-CASK	NeuroMab	Cat #: 75-000<br>RRID: AB_2068730	WB: 1:1000<br>ICC: 1:200
Rabbit anti-Ca^{2+} channel P/Q-type alpha-1A	Synaptic Systems	Cat #: 152 203<br>RRID: AB_2619841	ICC: 1:200
Rabbit anti-ERC1/2	Synaptic Systems	Cat #: 143003<br>RRID: AB_887715	WB: 1:1000<br>ICC: 1:200
Mouse anti-GFP	DSHB	Cat #: DSHB-GFP-4C9-b<br>RRID: AB_2617422	ICC: 1:500
Rabbit anti-GFP	Thermo Fisher Scientific	Cat #: A11122<br>RRID: AB_221569	WB: 1:1000
Mouse anti-FLAG	Sigma	Cat #: F1804<br>RRID: AB_262044	ICC: 1:200
Mouse anti-HA (HA.11)	BioLegend	Cat #: 901513<br>RRID: AB_2565335	ICC: 1:200
Mouse anti-HA (HA.11) Alexa-488-conjugated	BioLegend	Cat #: 901509<br>RRID: AB_2565072	ICC: 1:200
Rabbit anti-HA (C29F4)	Cell Signalling	Cat #: 3724<br>RRID: AB_1549585	ICC: 1:200
Rabbit anti-Homer1	Synaptic Systems	Cat #: 160003<br>RRID: AB_887730	WB: 1:1000
Rabbit anti-Liprin-α1	Gift from S. Schoch [1]	A121	WB: 1:200
Rabbit anti-Liprin-α2	Gift from S. Schoch [1]	A13	WB: 1:200
Rabbit anti-Liprin-α3	Gift from S. Schoch [1]	A115	WB: 1:200
Rabbit anti-Liprin-α4	Gift from S. Schoch [1]	A2	WB: 1:200
Chicken anti-MAP2	Encor	Cat #: CPCA-MAP2<br>RRID: AB_2138173	ICC: 1:1000
Rabbit anti-Mint1	Synaptic Systems	Cat #: 144103<br>RRID: AB_10635158	WB: 1:1000<br>ICC: 1:200
Rabbit anti-Munc13-1	Synaptic Systems	Cat #: 126103<br>RRID: AB_887733	WB: 1:1000<br>ICC: 1:200
Rabbit anti-Neuroligin-1	NeuroMab	Cat #: 75-160<br>RRID: AB_2235964	WB: 1:500
Rabbit anti-panNeurexin-1	Millipore	Cat #: ABN161-I<br>RRID: AB_10917110	WB: 1:1000
Rabbit anti-Piccolo	Synaptic Systems	Cat # 142 003<br>RRID: AB_2160182	ICC: 1:200
Mouse anti-PSD95	Thermo Fisher Scientific	Cat #: MA1-046<br>RRID: AB_2092361	WB: 1:500

Mouse anti-PSD95	NeuroMab	Cat #: 75-028(K28/43)<br>RRID: AB_2877189	ICC: 1:100
Rabbit anti-PSD95	Addgene	Cat #: 196561(K28/43)<br>RRID: AB_2928071	ICC: 1:100
Mouse anti-PTPRS	MediMabs	Cat #: MM-0020<br>RRID: AB_1808357	WB: 1:1000
Rabbit anti-RIM1	Synaptic Systems	Cat #: 140003<br>RRID: AB_887774	WB: 1:1000
Rabbit anti-RIM1/2	Synaptic Systems	Cat #: 140213<br>RRID: AB_2832237	ICC: 1:200
Rabbit anti-RIM-BP2	Synaptic Systems	Cat #: 316103<br>RRID: AB_2619739	WB: 1:1000<br>ICC: 1:200
Rabbit anti-Rab3a	Synaptic Systems	Cat #: 107 111<br>RRID: AB_887770	WB: 1:1000
Rabbit anti-SNAP25	Sigma	Cat #: S9684<br>RRID: AB_261576	WB: 1:2000
Rabbit anti-Synapsin	This paper	nc30-1<br>Custom-made by	WB: 1:1000<br>ICC: 1:1000
Rabbit anti-Synapsin	Gift from T. Südhof	E028<br>RRID: AB_2315400	ICC: 1:1000
Mouse anti-Synapsin 2	Sigma	Cat #: MABN1584	WB: 1:1000
Mouse anti-Synaptophysin1	Synaptic Systems	Cat #: 101 011<br>RRID: AB_887824	ICC: 1:200
Chicken anti-Synaptotagmin1	Aves Labs	Cat #: STG<br>RRID: AB_2313562	WB: 1:1000
Mouse anti-Syntaxin1A	Synaptic Systems	Cat #: 110 111<br>RRID: AB_887848	WB: 1:1000
Mouse anti-SV2	DSHB	Cat #: SV2-c<br>RRID: AB_2315387	ICC: 1:500
Mouse anti-Tuj1 (α-βIII-Tubulin)	BioLegend	Cat #: 801201<br>RRID: AB_2313773	WB: 1:5000<br>ICC: 1:1000
Rabbit anti-Veli1/2/3	Synaptic Systems	Cat #: 184002<br>RRID: AB_2281173	WB: 1:1000
Goat anti-Mouse Alexa Fluor 488	Thermo Fisher Scientific	Cat #: A-11001<br>RRID: AB_2534069	ICC: 1:1000
Goat anti-Mouse Alexa Fluor 568	Thermo Fisher Scientific	Cat #: A-11004<br>RRID: AB_2534072	ICC: 1:1000
Goat anti-Mouse Alexa Fluor 633	Thermo Fisher Scientific	Cat #: A-21052<br>RRID: AB_2535719	ICC: 1:600
Goat anti-Rabbit Alexa Fluor 405	Thermo Fisher Scientific	Cat #: A-31556<br>RRID: AB_221605	ICC: 1:1000
Goat anti-Rabbit Alexa Fluor 488	Thermo Fisher Scientific	Cat #: A-32731<br>RRID: AB_2633280	ICC: 1:1000
Goat anti-Rabbit Alexa Fluor 568	Thermo Fisher Scientific	Cat #: A-11011<br>RRID: AB_143157	ICC: 1:1000
Goat anti-Mouse Alexa Fluor 594	Thermo Fisher Scientific	Cat #: A-11032<br>RRID: AB_2534091	ICC: 1:1000
Goat anti-Rabbit Alexa Fluor 633	Thermo Fisher Scientific	Cat #: A-21071<br>RRID: AB_2535732	ICC: 1:600

Goat anti-Rabbit Alexa Fluor 647	Thermo Fisher Scientific	Cat #: A-21245<br>RRID: AB_2535813	ICC: 1:600
Goat anti-Chicken-CF405M	Sigma	Cat #: SAB4600466	ICC: 1:1000
Goat anti-Chicken Alexa Fluor 633	Thermo Fisher Scientific	Cat #: A-21103<br>RRID: AB_2535756	ICC: 1:600
Goat anti-Mouse 680RD	LI-COR	Cat #: 925-68070<br>RRID: AB_2651128	WB: 1:10,000
Goat anti-Mouse 800CW	LI-COR	Cat #: 925-32210<br>RRID: AB_2687825	WB: 1:10,000
Goat anti-Rabbit 680RD	LI-COR	Cat #: 925-68071<br>RRID: AB_2721181	WB: 1:10,000
Goat anti-Rabbit 800CW	LI-COR	Cat #: 925-32211<br>RRID: AB_621843	WB: 1:10,000
Donkey anti-Chicken 680RD	LI-COR	Cat #: 926-68075<br>RRID: AB_10974977	WB: 1:10,000

SUPPLEMENTARY INFORMATION REFERENCES

1. Zürner, M., Mittelstaedt, T., tom Dieck, S., Becker, A. & Schoch, S. Analyses of the spatiotemporal expression and subcellular localization of liprin-α proteins. *The Journal of Comparative Neurology* **519**, 3019-3039 (2011).
2. Montoliu-Gaya, L.*, et al.* CA10 regulates neurexin heparan sulfate addition via a direct binding in the secretory pathway. *EMBO Rep* **22**, e51349 (2021).
3. Maas, C.*, et al.* Formation of Golgi-Derived Active Zone Precursor Vesicles. *Journal of Neuroscience* **32**, 11095-11108 (2012).
4. Gokce, O. & Südhof, T.C. Membrane-tethered monomeric neurexin LNS-domain triggers synapse formation. *The Journal of neuroscience : the official journal of the Society for Neuroscience* **33**, 14617-14628 (2013).
5. Pak, C.*, et al.* Human Neuropsychiatric Disease Modeling using Conditional Deletion Reveals Synaptic Transmission Defects Caused by Heterozygous Mutations in NRXN1. *Cell Stem Cell* **17**, 316-328 (2015).
6. Grimm, D.*, et al.* In vitro and in vivo gene therapy vector evolution via multispecies interbreeding and retargeting of adeno-associated viruses. *J Virol* **82**, 5887-5911 (2008).
