# Supplementary material for: Liprin-α proteins are master regulators of human presynapse assembly
Source: Nat Neurosci. 2024 Mar 12;27(4):629–42. doi: 10.1038/s41593-024-01592-9 (PMC11001580; doi:10.1038/s41593-024-01592-9)
Supplement: Supplementary file 2 — Reporting Summary [file 41593_2024_1592_MOESM2_ESM.pdf]

nature portfolio

Corresponding author(s): Fredrik H. Sterky

Last updated by author(s): Jan 8, 2024

Reporting Summary

Nature Portfolio wishes to improve the reproducibility of the work that we publish. This form provides structure for consistency and transparency in reporting. For further information on Nature Portfolio policies, see our Editorial Policies and the Editorial Policy Checklist.

Statistics

For all statistical analyses, confirm that the following items are present in the figure legend, table legend, main text, or Methods section.

n/a	Confirmed	
☐	☒	The exact sample size (*n*) for each experimental group/condition, given as a discrete number and unit of measurement
☐	☒	A statement on whether measurements were taken from distinct samples or whether the same sample was measured repeatedly
☐	☒	The statistical test(s) used AND whether they are one- or two-sided<br>*Only common tests should be described solely by name; describe more complex techniques in the Methods section.*
☐	☒	A description of all covariates tested
☐	☒	A description of any assumptions or corrections, such as tests of normality and adjustment for multiple comparisons
☐	☒	A full description of the statistical parameters including central tendency (e.g. means) or other basic estimates (e.g. regression coefficient) AND variation (e.g. standard deviation) or associated estimates of uncertainty (e.g. confidence intervals)
☐	☒	For null hypothesis testing, the test statistic (e.g. *F*, *t*, *r*) with confidence intervals, effect sizes, degrees of freedom and *P* value noted<br>*Give P values as exact values whenever suitable.*
☒	☐	For Bayesian analysis, information on the choice of priors and Markov chain Monte Carlo settings
☒	☐	For hierarchical and complex designs, identification of the appropriate level for tests and full reporting of outcomes
☒	☐	Estimates of effect sizes (e.g. Cohen's *d*, Pearson's *r*), indicating how they were calculated

Our web collection on statistics for biologists contains articles on many of the points above.

Software and code

Policy information about availability of computer code

Data collection	NIS elements AR software v. 5.21.01 (Nikon Instruments), LASX (Leica), Clampex, Zen Black 3.0 SR, v16.0.17.306 (Zeiss) and Image Studio 5.2 (Li-COR)
Data analysis	Clampfit v. 10.2 (Molecular Devices), Igor Pro v. 4.07 (WaveMetrics, Lake Oswego, OR), NIS elements AR v. 5.21.01 (Nikon Instruments), LASX (Leica), ImageJ/Fiji v. 2.3.0/1.53f, Excel v. 16 (Microsoft), Image Studio v. 5.2 (Li-COR), Geneious Prime v. 11.0.15 (BioMatters), TIDE 3.3.0 (https://tide.nki.nl), R 4.2.2 (R project consortium), MATLAB R2022a (MathWorks) and Prism 9 (GraphPad). Design of sgRNA sequences was aided by CHOPCHOP v. 3 (http://chopchop.cbu.uib.no).

For manuscripts utilizing custom algorithms or software that are central to the research but not yet described in published literature, software must be made available to editors and reviewers. We strongly encourage code deposition in a community repository (e.g. GitHub). See the Nature Portfolio guidelines for submitting code & software for further information.

Data

Policy information about availability of data

All manuscripts must include a data availability statement. This statement should provide the following information, where applicable:

- Accession codes, unique identifiers, or web links for publicly available datasets
- A description of any restrictions on data availability
- For clinical datasets or third party data, please ensure that the statement adheres to our policy

Numerical source data are provided within this paper. Additional data that support the findings of this study are available from the corresponding author upon reasonable request.

Research involving human participants, their data, or biological material

Policy information about studies with human participants or human data. See also policy information about sex, gender (identity/presentation), and sexual orientation and race, ethnicity and racism.

Reporting on sex and gender	n/a
Reporting on race, ethnicity, or other socially relevant groupings	n/a
Population characteristics	n/a
Recruitment	n/a
Ethics oversight	n/a

Note that full information on the approval of the study protocol must also be provided in the manuscript.

Field-specific reporting

Please select the one below that is the best fit for your research. If you are not sure, read the appropriate sections before making your selection.

☒ Life sciences ☐ Behavioural & social sciences ☐ Ecological, evolutionary & environmental sciences

For a reference copy of the document with all sections, see nature.com/documents/nr-reporting-summary-flat.pdf

Life sciences study design

All studies must disclose on these points even when the disclosure is negative.

Sample size	We did not use power analysis or other statistical method to determine the sample size. The number of datapoints and repetitions (independent batches of cultured neurons) was determined in accordance with our previous studies (Acuna et al., 2016; Sterky et al., 2017), as well as based on previous landmark papers in the field (Patzke et al., 2019; Pak et al, 2015).
Data exclusions	No data points were excluded from statistical analysis.
Replication	Typically 3 independent replicates were performed, as indicated in figure legends and Table S1, and results merged. All experiments were repeated at least once, except for screening PCRs, the western blot confirming liprin-alpha1 deletion in human astrocytes (Extended data fig 1c) and the liprin-alpha3 rescue condition for electron microscopy.
Randomization	Allocation (e.g. distribution of different experimental lentiviruses on separate cover slips, order of analysis etc.) was random.
Blinding	Microscopy and electrophysiology experiments were performed on coded samples to blind the experimenter from the genotype. However, in some experiments strong differences in phenotypic readouts made the genotype obvious to the experimenter.

Reporting for specific materials, systems and methods

We require information from authors about some types of materials, experimental systems and methods used in many studies. Here, indicate whether each material, system or method listed is relevant to your study. If you are not sure if a list item applies to your research, read the appropriate section before selecting a response.

Materials & experimental systems

n/a	Involved in the study
☐	☒ Antibodies
☐	☒ Eukaryotic cell lines
☒	☐ Palaeontology and archaeology
☐	☒ Animals and other organisms
☒	☐ Clinical data
☒	☐ Dual use research of concern
☒	☐ Plants

Methods

n/a	Involved in the study
☒	☐ ChIP-seq
☒	☐ Flow cytometry
☒	☐ MRI-based neuroimaging

Antibodies

Antibodies used

1. Mouse anti-β-actin Sigma Cat #: A5441; RRID: AB_476744
2. Rabbit anti-Bassoon Sigma Cat #: SAB5200101
3. Mouse anti-CASK NeuroMab Cat #: 75-000; RRID: AB_2068730
4. Rabbit anti-Ca2+ channel P/Q-type alpha-1A Synaptic Systems Cat #: 152 203; RRID: AB_2619841
5. Rabbit anti-ERC1/2 Synaptic Systems Cat #: 143003; RRID: AB_887715
6. Mouse anti-GFP DSHB Cat #: DSHB-GFP-4C9-b; RRID: AB_2617422
7. Rabbit anti-GFP Thermo Fisher Scientific Cat #: A11122; RRID: AB_221569
8. Mouse anti-FLAG Sigma Cat #: F1804; RRID: AB_262044
9. Mouse anti-HA (HA.11) Alexa-488-conjugated BioLegend Cat #: 901509; RRID: AB_2565072
10. Rabbit anti-HA (C29F4) Cell Signalling Cat #: 3724; RRID: AB_1549585
11. Rabbit anti-Homer1 Synaptic Systems Cat #: 160003; RRID: AB_887730
12. Rabbit anti-Liprin-α1 Gift from S. Schoch A121
13. Rabbit anti-Liprin-α2 Gift from S. Schoch A13
14. Rabbit anti-Liprin-α3 Gift from S. Schoch A115
15. Rabbit anti-Liprin-α4 Gift from S. Schoch A2
16. Chicken anti-MAP2 Encor Cat #: CPCA-MAP2; RRID: AB_2138173
17. Rabbit anti-Mint1 Synaptic Systems Cat #: 144103; RRID: AB_10635158
18. Rabbit anti-Munc13-1 Synaptic Systems Cat #: 126103; RRID: AB_887733
19. Rabbit anti-Neuroligin-1 NeuroMab Cat #: 75-160; RRID: AB_2235964
20. Rabbit anti-panNeurexin-1 Millipore Cat #: ABN161-I; RRID: AB_10917110
21. Rabbit anti-Piccolo Synaptic Systems Cat # 142003; RRID: AB_2160182
22. Mouse anti-PSD95 Thermo Fisher Scientific Cat #: MA1-046; RRID: AB_2092361
23. Mouse anti-PSD95 NeuroMab Cat #: 75-028(K28/43); RRID: AB_2877189
24. Rabbit anti-PSD95 Addgene Cat #: 196561(K28/43); RRID: AB_2928071
25. Mouse anti-PTPRS MediMabs Cat #: MM-0020; RRID: AB_1808357
26. Rabbit anti-RIM1 Synaptic Systems Cat #: 140003; RRID: AB_887774
27. Rabbit anti-RIM1/2 Synaptic Systems Cat #: 140213; RRID: AB_2832237
28. Rabbit anti-RIM-BP2 Synaptic Systems Cat #: 316103; RRID: AB_2619739
29. Rabbit anti-Rab3a Synaptic Systems Cat #: 107111; RRID: AB_887770
30. Rabbit anti-SNAP25 Sigma Cat #: S9684; RRID: AB_261576
31. Rabbit anti-Synapsin This paper nc30-1; custom-made by Proteogenix using same antigen as E028
32. Rabbit anti-Synapsin Gift from T. Südhof E028; RRID: AB_2315400
33. Mouse anti-Synapsin 2 Sigma Cat #: MABN1584
34. Mouse anti-Synaptophysin1 Synaptic Systems Cat #: 101011; RRID: AB_887824
35. Chicken anti-Synaptotagmin1 Aves Labs Cat #: STG; RRID: AB_2313562
36. Mouse anti-Syntaxin1A Synaptic Systems Cat #: 110111; RRID: AB_887848
37. Mouse anti-SV2 DSHB Cat #: SV2-c; RRID: AB_2315387
38. Mouse anti-Tuj1 (α-βIII-Tubulin) BioLegend Cat #: 801201; RRID: AB_2313773
39. Rabbit anti-Veli1/2/3 Synaptic Systems Cat #: 184002; RRID: AB_2281173
40. Goat anti-Mouse Alexa Fluor 488 Thermo Fisher Scientific Cat #: A-11001; RRID: AB_2534069
41. Goat anti-Mouse Alexa Fluor 568 Thermo Fisher Scientific Cat #: A-11004; RRID: AB_2534072
42. Goat anti-Mouse Alexa Fluor 633 Thermo Fisher Scientific Cat #: A-21052; RRID: AB_2535719
43. Goat anti-Rabbit Alexa Fluor 405 Thermo Fisher Scientific Cat #: A-31556; RRID: AB_221605
44. Goat anti-Rabbit Alexa Fluor 488 Thermo Fisher Scientific Cat #: A-32731; RRID: AB_2633280
45. Goat anti-Rabbit Alexa Fluor 568 Thermo Fisher Scientific Cat #: A-11011; RRID: AB_143157
46. Goat anti-Mouse Alexa Fluor 594 Thermo Fisher Scientific Cat #: A-11032; RRID: AB_2534091
47. Goat anti-Rabbit Alexa Fluor 633 Thermo Fisher Scientific Cat #: A-21071; RRID: AB_2535732
48. Goat anti-Rabbit Alexa Fluor 647 Thermo Fisher Scientific Cat #: A-21245; RRID: AB_2535813
49. Goat anti-Chicken-CF405M Sigma Cat #: SAB4600466
50. Goat anti-Chicken Alexa Fluor 633 Thermo Fisher Scientific Cat #: A-21103; RRID: AB_2535756
51. Goat anti-Mouse 680RD LI-COR Cat #: 925-68070; RRID: AB_2651128
52. Goat anti-Mouse 800CW LI-COR Cat #: 925-32210; RRID: AB_2687825
53. Goat anti-Rabbit 680RD LI-COR Cat #: 925-68071; RRID: AB_2721181
54. Goat anti-Rabbit 800CW LI-COR Cat #: 925-32211; RRID: AB_621843
55. Donkey anti-Chicken 680 RD LI-COR Cat #: 926-68075; RRID: AB_10974977

Validation	1. Western blot, dilution 1:1000. Validated for WB by supplier (sigmaaldrich.com) and 10,000+ previous publications<br>2. ICC, dilution 1:200. Validated for ICC by supplier (sigmaaldrich.com) and previous publications (e.g. PMID: 31585084)<br>3. Western blot, dilution 1:1000; ICC, dilution 1:200. Validated for WB by supplier (neuromab.ucdavis.edu) and previous publications (e.g. PMID: 33037075) and for ICC by previous publications (e.g. PMID: 29983322, 29610457)<br>4. ICC, dilution 1:200. Validated for ICC by supplier (sysy.com) and previous publications (e.g. PMID: 27537483)<br>5. Western blot, dilution 1:1000; ICC, dilution 1:200. Validated for WB and ICC by supplier (sysy.com) and previous publications (e.g. PMID: 35443170, 23751498)<br>6. ICC, dilution 1:500. Validated for ICC by supplier (dshb.biology.uiowa.edu), previous publications (e.g. PMID: 33037075) and negative controls<br>7. Western blot, dilution 1:1000. Validated for WB by supplier (thermofisher.com) and 800+ previous publications<br>8. ICC, dilution 1:200. Validated for ICC by supplier (sigmaaldrich.com), 200+ previous publications and negative controls<br>9. ICC, dilution 1:200. Validated for ICC by previous publications (e.g. PMID: 26279266, 31262725) and negative controls<br>10. ICC, dilution 1:200. Validated for ICC by 98 previous publications and negative controls<br>11. Western blot, dilution 1:1000. Validated for WB by supplier (sysy.com) and previous publications (e.g. PMID: 35035429)<br>12-15. Western blot, dilution 1:200. Validated for WB by (PMID: 21618221) and this paper (KO-verified)<br>16. ICC, dilution 1:1000. Validated for ICC by supplier (encorebio.com) and by previous publications (e.g. PMID: 33646123)<br>17. Western blot, dilution 1:1000. Validated for WB by supplier (sysy.com) and previous publications (e.g. PMID: 34158621)<br>18. Western blot, dilution 1:1000; ICC, dilution 1:200. Validated for WB and ICC by supplier (sysy.com) and previous publications (e.g. PMID: 36398873)<br>19. Western blot, dilution 1:500. Validated for WB by supplier (neuromab.ucdavis.edu) and previous publications (PMID: 27869801)<br>20. Western blot, dilution 1:1000. Validated for WB by previous publications (e.g., PMID: 32706374, 30100184)<br>21. ICC, dilution 1:200. Validated for ICC by supplier (sysy.com) and previous publications (e.g. PMID: 31585084)<br>22. Western blot, dilution 1:500. Validated for WB by supplier (thermofisher.com) and by previous publications (e.g. PMID: 35532105)<br>23. ICC, dilution 1:100. Validated by supplier (neuromab.ucdavis.edu) and previous publications (e.g. PMID: 35532105)<br>24. ICC, dilution 1:100. Same clone as #22, RRID: AB_2877189<br>25. Western blot, dilution 1:1000. Validated for WB by supplier (medimabs.com) and by previous publications (e.g. PMID: 29934346)<br>26. Western blot, dilution 1:1000. Validated for WB by supplier (sysy.com) and previous publications (e.g. PMID: 32521280)<br>27. ICC, dilution 1:200. Validated for ICC by supplier (sysy.com)<br>28. Western blot, dilution 1:1000; ICC, dilution 1:200. Validated for WB and ICC by supplier (sysy.com) and previous publications (e.g. PMID: 27537484, 35443170)<br>29. Western blot, dilution 1:1000. Validated for WB by supplier (sysy.com) and previous publications (e.g. PMID: 10407024)<br>30. Western blot, dilution 1:1000. Validated for WB by supplier (sigmaaldrich.com) and previous publications (e.g. PMID: 34931070)<br>31. ICC, dilution 1:1000. Side-by-side comparisons with E028<br>32. ICC, dilution 1:1000. Validated by previous publications (e.g. PMID: 28154140, 33586859, 21241895)<br>33. Western blot, dilution 1:1000. Validated for WB by supplier (sigmaaldrich.com)<br>34. Western blot, dilution 1:1000; ICC, dilution 1:200. Validated for WB and ICC by supplier (sysy.com) and previous publications (e.g. PMID: 34031393)<br>35. Western blot, dilution 1:1000. Validated by supplier (aveslabs.com)<br>36. Western blot, dilution 1:1000. Validated for WB by supplier (sysy.com) and previous publications (e.g. PMID: 33730593)<br>37. ICC, dilution 1:500. Validated for ICC by supplier (dshb.biology.uiowa.edu) and previous publications (e.g. PMID: 32347002)<br>38. Western blot, dilution 1:1000; ICC, dilution 1:1000. Validated by supplier (biolegend.com) and previous publications (e.g. PMID: 32385372)<br>39. Western blot, dilution 1:1000. Validated for WB by supplier (sysy.com) and previous publications (e.g. PMID: 36137748)<br>40-55. All secondaries extensively validated by vendors and often in-house through omission of primary antibodies.

Eukaryotic cell lines

Policy information about cell lines and Sex and Gender in Research

Cell line source(s)	H9 (WiCell; RRID: CVCL_9773; hPSCreg: WAe009-A), HEK293T/17 (ATCC, CRL-11268), HeLa (RRID:CVCL_0030, ATCC-CCL-2)
Authentication	Cell lines used were not authenticated beyond origin, expected morphology and growth.
Mycoplasma contamination	All cell lines were tested negative for mycoplasma contamination by PCR.
Commonly misidentified lines (See ICLAC register)	No commonly misidentified lines were used.

Animals and other research organisms

Policy information about studies involving animals; ARRIVE guidelines recommended for reporting animal research, and Sex and Gender in Research

Laboratory animals	Pups (0.5-2.5 days old) from C57Bl/6 mice were used to establish primary glia cell cultures
Wild animals	No wild animals were used in the study
Reporting on sex	Pups of both sexes were used, and pooled.
Field-collected samples	No field-collected samples were used in the study

Ethics oversight	Animal procedures were approved by the Swedish Board of Agriculture, the Robert Koch Institute (Germany), and the 'Regierungsprasidium' Karlsrhue (Germany)

Note that full information on the approval of the study protocol must also be provided in the manuscript.
