# Supplementary figures and images for: Liprin-α proteins are master regulators of human presynapse assembly
Source: Nat Neurosci. 2024 Mar 12;27(4):629–42. doi: 10.1038/s41593-024-01592-9 (PMC11001580; doi:10.1038/s41593-024-01592-9)

Figure 1C

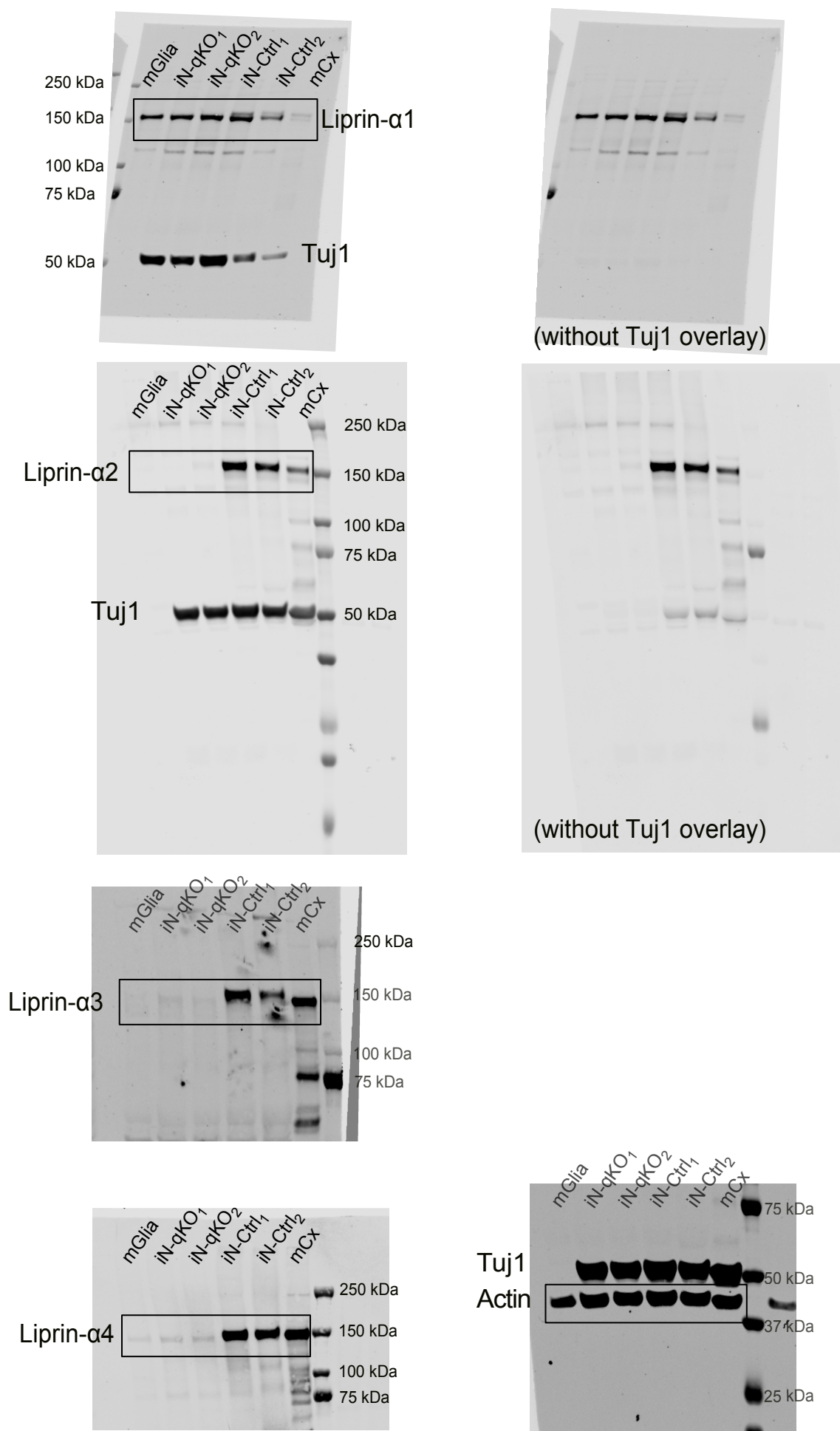

Figure 1F

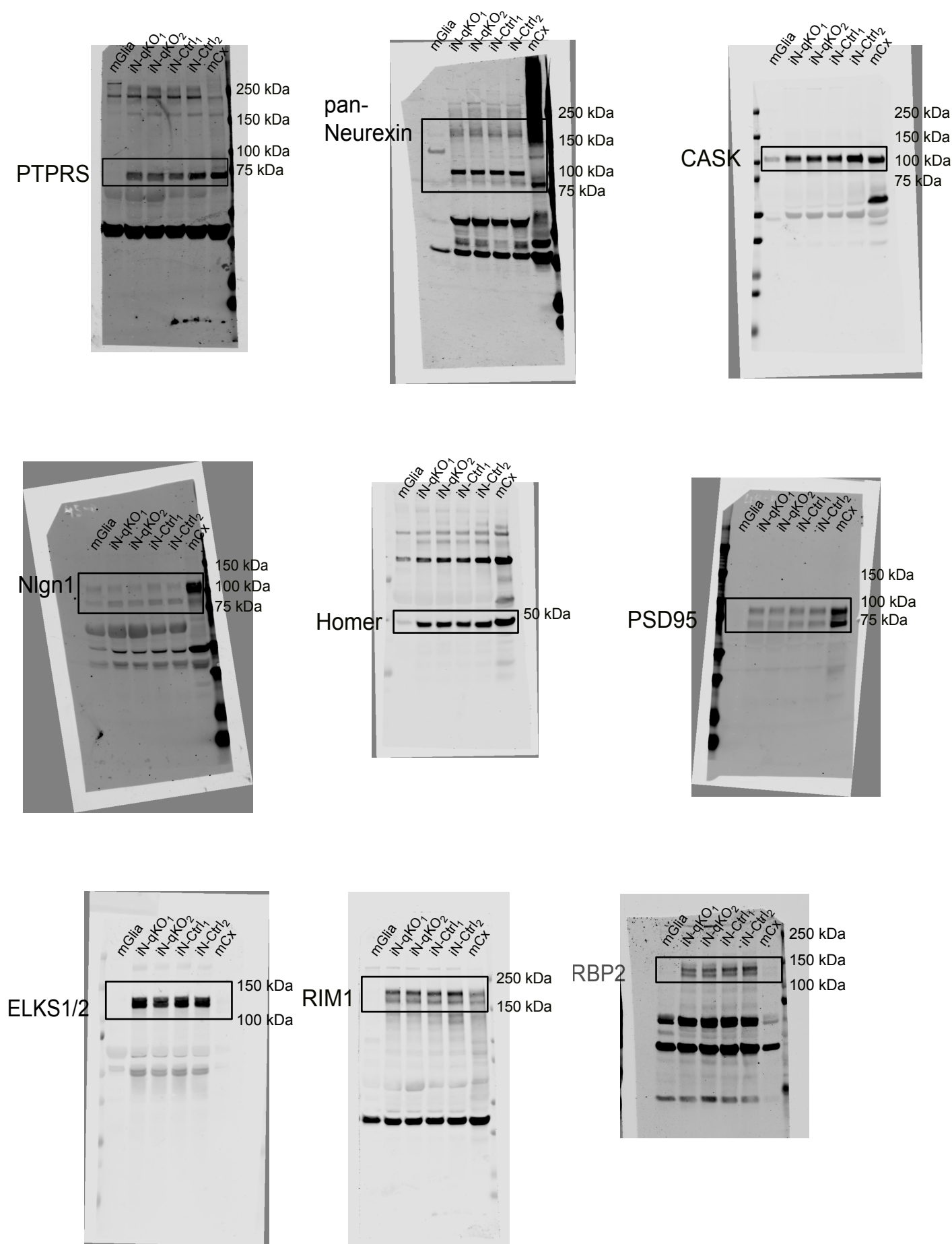

**Figure 1F (cont.)**

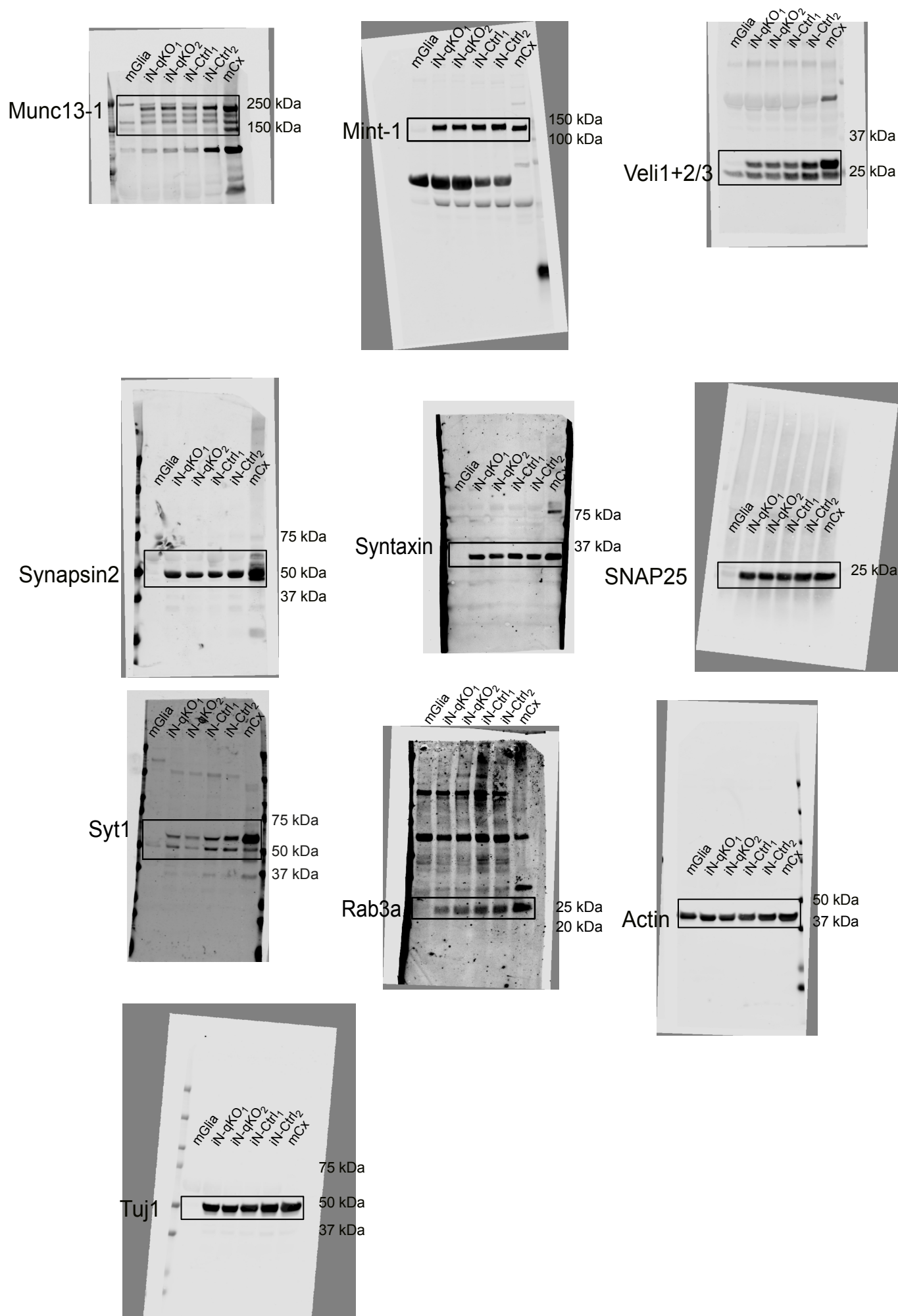

**Figure 1F (replicates for quantifications)**

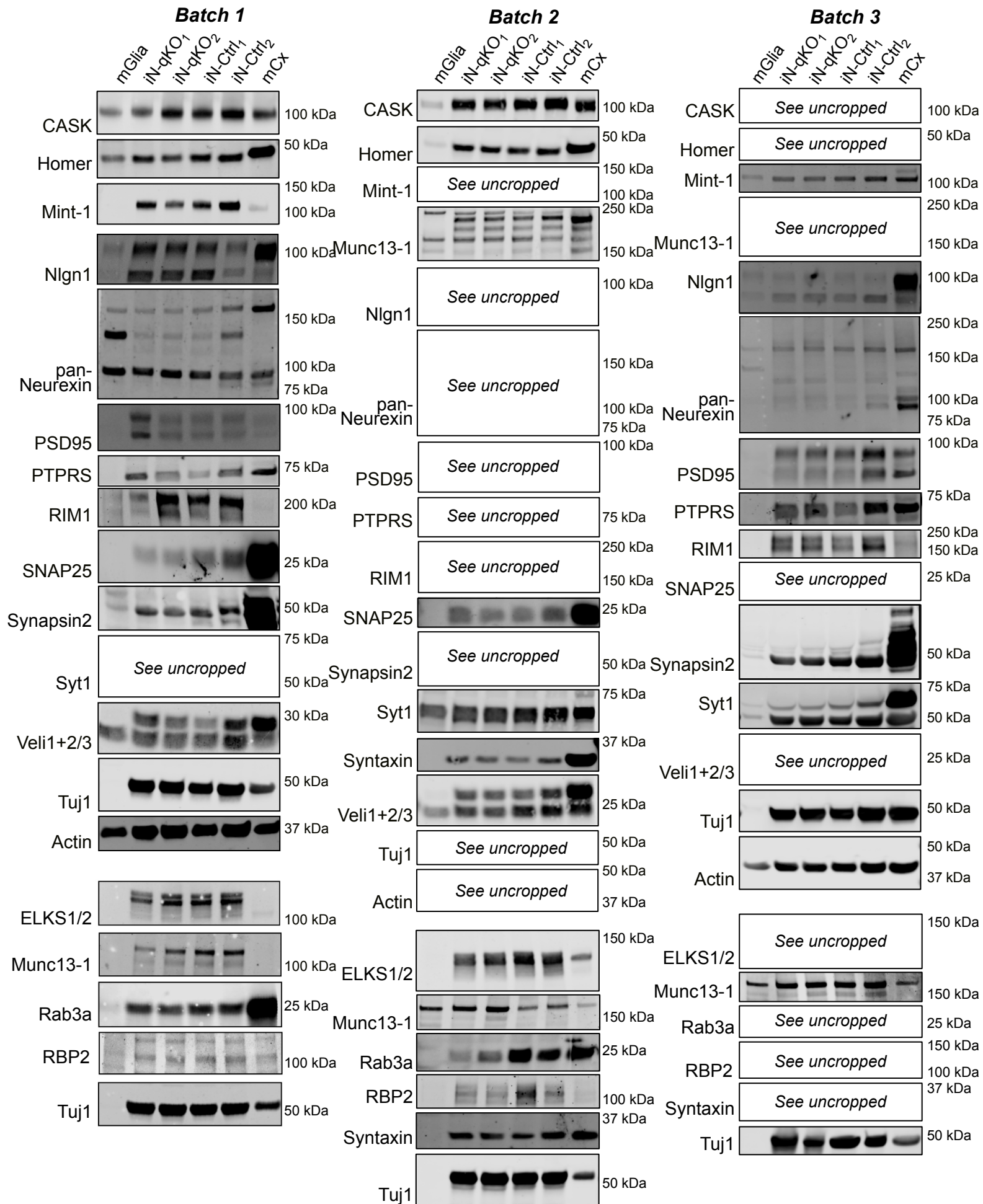

Figure 1F cont.

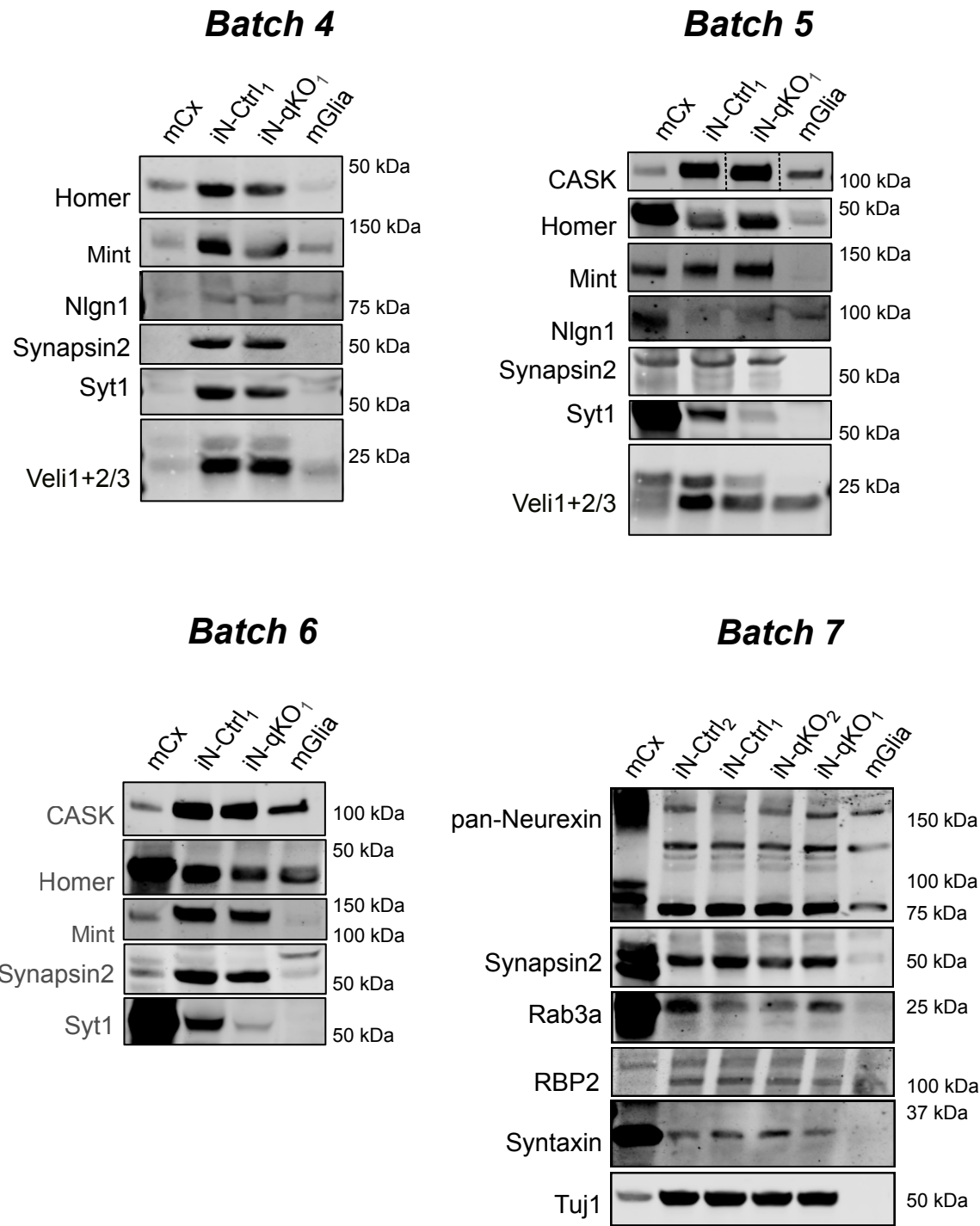

Supplement: Supplementary file 4 — Unprocessed western blots. [file 41593_2024_1592_MOESM4_ESM.pdf]

Figure 5A

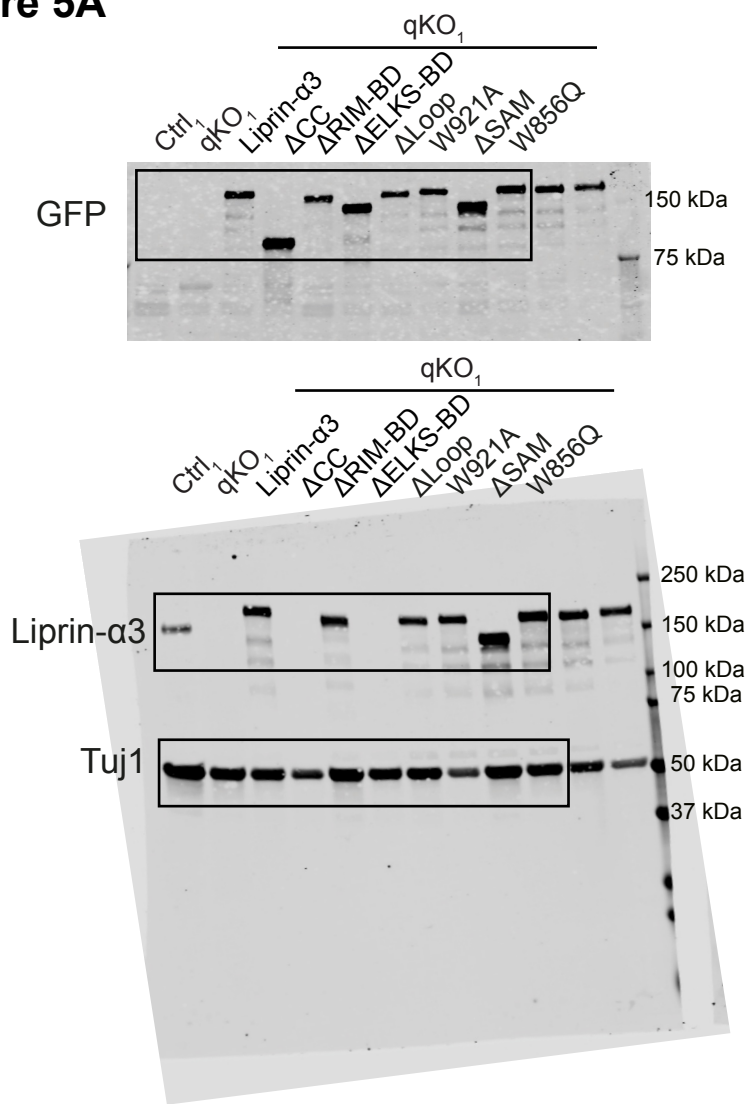

Supplement: Supplementary file 9 — Unprocessed western blots. [file 41593_2024_1592_MOESM9_ESM.pdf]

**Ext Data Figure 1C**

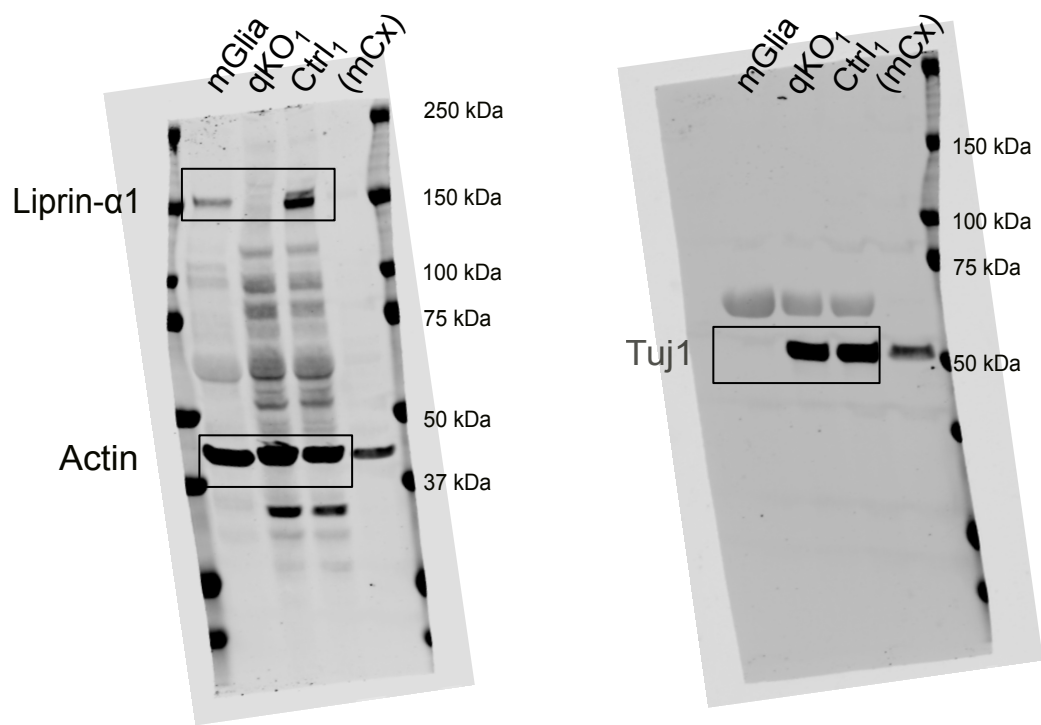

**Ext Data Figure 1H**

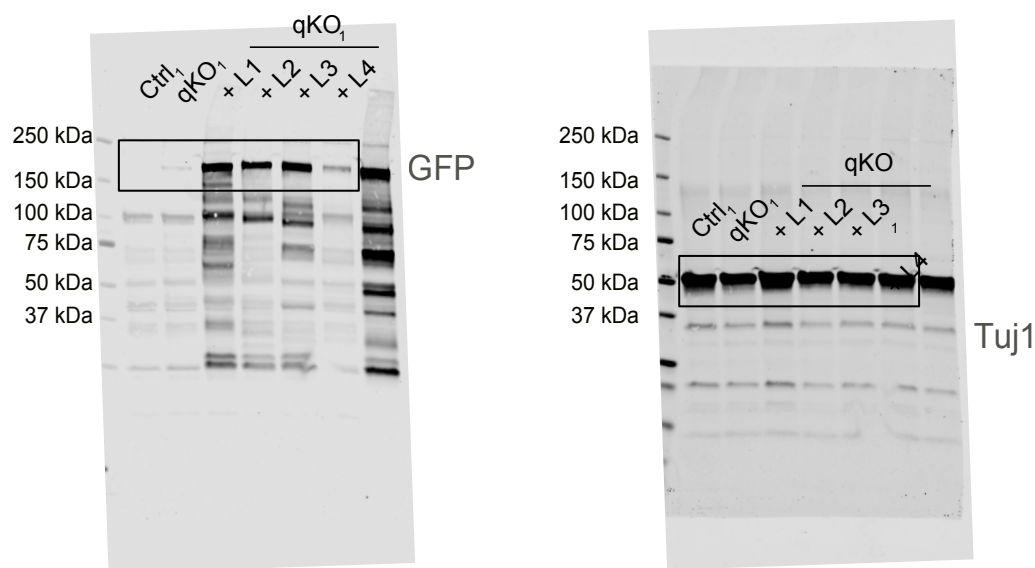

Supplement: Supplementary file 12 — Unprocessed western blots. [file 41593_2024_1592_MOESM12_ESM.pdf]
